# Control of impulsivity by G_i_-protein signalling in layer-5 pyramidal neurons of the anterior cingulate cortex

**DOI:** 10.1038/s42003-021-02188-w

**Published:** 2021-06-02

**Authors:** Bastiaan van der Veen, Sampath K. T. Kapanaiah, Kasyoka Kilonzo, Peter Steele-Perkins, Martin M. Jendryka, Stefanie Schulz, Bosiljka Tasic, Zizhen Yao, Hongkui Zeng, Thomas Akam, Janet R. Nicholson, Birgit Liss, Wiebke Nissen, Anton Pekcec, Dennis Kätzel

**Affiliations:** 1 Institute of Applied Physiology, Ulm University, Ulm, Germany; 2 Allen Institute for Brain Science, Seattle, WA USA; 3 Department of Experimental Psychology, University of Oxford, Oxford, UK; 4 Boehringer Ingelheim Pharma GmbH & Co. KG, Div. Research Germany, Biberach an der Riss, Germany; 5 Linacre College and New College, University of Oxford, Oxford, UK

**Keywords:** Diseases of the nervous system, Neural circuits, Cognitive control, Target identification

## Abstract

Pathological impulsivity is a debilitating symptom of multiple psychiatric diseases with few effective treatment options. To identify druggable receptors with anti-impulsive action we developed a systematic target discovery approach combining behavioural chemogenetics and gene expression analysis. Spatially restricted inhibition of three subdivisions of the prefrontal cortex of mice revealed that the anterior cingulate cortex (ACC) regulates premature responding, a form of motor impulsivity. Probing three G-protein cascades with designer receptors, we found that the activation of G_i_-signalling in layer-5 pyramidal cells (L5-PCs) of the ACC strongly, reproducibly, and selectively decreased challenge-induced impulsivity. Differential gene expression analysis across murine ACC cell-types and 402 GPCRs revealed that - among G_i_-coupled receptor-encoding genes - *Grm2* is the most selectively expressed in L5-PCs while alternative targets were scarce. Validating our approach, we confirmed that mGluR2 activation reduced premature responding. These results suggest G_i_-coupled receptors in ACC L5-PCs as therapeutic targets for impulse control disorders.

## Abstract (web summary)

In order to identify receptors that may serve as therapeutic targets in the treatment of impulse control disorders, van der Veen et al developed a systematic target discovery approach combining behavioural chemogenetics and gene expression analysis. In doing so, they identified Gi-coupled receptors in layer-5 pyramidal cells of the anterior cingulate cortex as potential therapeutic targets, which could pave the way for the development of treatments.

## Introduction

Impulsivity is the inclination to act rapidly at the expense of adequate foresight, action planning or execution^1,2^, and may involve a failure of either motor control (motor impulsivity) or decision making (decision impulsivity), or both^3^. One large-scale study found that 17% of the general adult US population would be diagnosed as *impulsive* according to self-reports registered through clinical questionnaires used for diagnoses of impulse control disorders^4^. Impulsivity may constitute a personality trait entailing a higher risk to conduct harmful behaviours^4^, including the abuse of various substances like cocaine, opioids and alcohol^5,6^. Thereby, high trait impulsivity represents a key risk factor determining development, severity and relapse vulnerability in addiction^1,7–9^. However, pathologically high impulsivity also constitutes a key symptom of many psychiatric and neurological diseases including, again, drug addiction, pathological gambling, affective disorders, borderline personality disorder, Parkinson’s disease and especially childhood impulse control disorders like ADHD^1^.

Effective and selective treatment options for impaired impulse control are scarce^1^, despite the high clinical need and relatively well-established behavioural assays to measure impulsivity. Moreover, pre-clinical studies mostly focused on the dissection of the mechanisms of *known* molecular modulators of impulsivity such as dopamine or serotonin^6^, that have so far not led to novel treatments.

In contrast, we here develop a systematic, unbiased approach to discover novel druggable targets to improve impulse control with potentially higher specificity. We combined (1) a well-validated behavioural assay—the 5-Choice-Serial-Reaction-Time Task (5-CSRTT)^3,10^—that measures *premature responding*, a form of motor impulsivity with high clinical relevance for addiction and ADHD^1,6^, with (2) the chemogenetic modulation of intracellular signalling in distinct brain regions and cell types^11^ and (3) the identification of differentially expressed genes in putative target cell-types using single-cell transcriptomic data analysis^12–14^.

**Fig. 1:**
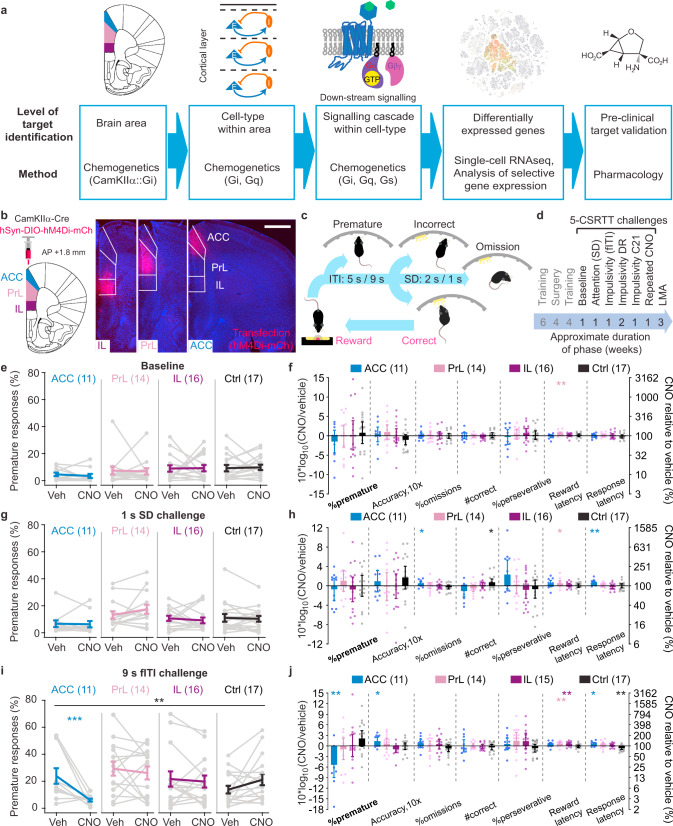
Activation of the G_i_-cascade in pyramidal cells of the ACC reduces premature responding. **a** Approach of narrowing down a pharmacological target, which starts with genetically targeted manipulation of neural activity of a brain area (left), a specific cell-type within that area (mid left), intracellular signalling within that cell-type (middle), and is followed by the identification of activity-regulating genes (especially receptors) within that cell-type whose therapeutic action is finally validated pharmacologically. G_i_, G_q_ and G_s_ refer to G-protein cascades. **b** Selective transfection of the infralimbic (IL, purple), prelimbic (PrL, lilac) and anterior cingulate cortex (ACC, blue) subregions of the PFC with the fusion protein hM4Di-mCherry in CamKIIα-Cre mice, as indicated by native mCherry-fluorescence in a brain slice that contains all three subregions (approximate anterior-posterior, AP, distance from Bregma: 1.8 mm; scale bar, 1 mm). **c** Operant cycle of the 5-CSRTT (see Methods; ITI, inter-trial interval; SD, stimulus duration). **d** Temporal order of surgery and chemogenetic/behavioural testing that each mouse of the cohort underwent, including the duration for each test phase; all tests were conducted within-subject with vehicle and 10 mg/kg CNO applications separated by 3–7 d. **e**, **g**, **i** Absolute values of premature responses (as % of the number of trials; indicator of impulsivity) after injection of vehicle (Veh) or CNO for the four subgroups of the cohort [ACC (blue), PrL (lilac), IL (purple), controls (Ctrl; black)] and 5-CSRTT protocols named above the panels. Black stars indicate significant drug-group interaction (RM-ANOVA); coloured stars indicate Sidak paired post-hoc test between vehicle and CNO conditions in the colour-coded group. CNO-induced changes in the 5-CSRTT baseline protocol (**f**), attention challenge (**h**) and impulsivity challenge (**j**), respectively, measured as log_10_-transform of the within-subject ratio (value after CNO/value after vehicle) for relevant behavioural performance parameters on the 5-CSRTT are shown for the four groups as indicated in the legend; asterisks indicate one-sample *t* test against 0 (identity of parameter under both conditions); accuracy is multiplied by 10 as this parameter shows comparatively small variations. *N*-numbers stated in each panel. See Supplementary Tables 3–6 for reasons for varying *N*-numbers across experiments, further statistical analysis, and absolute response numbers. *P < 0.05; **P < 0.01; ***P ≤ 0.001; error bars, s.e.m. in **e**, **g**, **i**, otherwise C.I.; individual dots or dot-lines represent subjects.

Applying this top-down approach, a target can be narrowed down from a specific *brain region*, to a *cell-type* within that brain region, and to a *molecular cascade* within that cell-type whose modulation causes a therapeutic effect at the preclinical level (Fig. 1a)^11^. Combining this knowledge with single-cell genomics data allows candidate druggable targets to be identified, in form of receptors that are preferentially expressed in the target cell population and that activate the signalling cascade found to have a therapeutic effect. Development or repurposing of drugs towards this molecular target allow confirmation of this approach pre-clinically (Fig. 1a), or even clinically.

In our search for drug targets with anti-impulsive action, we assumed the following constraints: (1) We focused on G-protein-coupled receptors (GPCRs). Impulse control is sensitive to several common GPCRs^6,15^, but this druggable target class contains several hundred further members that might be exploited for specific modulation of neuronal circuits^16–18^. (2) We investigated the prefrontal cortex (PFC), as it has been repeatedly implicated in impulse control in rodents (see literature summary in Supplementary Table 1). As a first step, we conducted a systematic comparison of transient manipulations of three subdivisions of the murine PFC and then narrowed down a cellular and molecular target as outlined above (Fig. 1a).

## Results

### Chemogenetic inhibition of ACC pyramidal cells reduces challenge-evoked impulsivity and improves attention

To identify the PFC subdivision that most consistently affects premature responding in mice, we selectively and separately transduced the inhibitory DREADD hM4Di^19^ to excitatory cells of the infralimibic (IL), prelimbic (PrL), and anterior cingulate cortex (ACC) in CamKIIα-Cre mice pre-trained in the 5-CSRTT (Fig. 1b–d; see Methods, Supplementary Tables 2, 3). Mice were initially tested under vehicle and 10 mg/kg CNO^20^ in three conditions—the baseline protocol, an attention challenge with stimulus duration (SD) shortened from 2 s to 1 s (SD-challenge), and an impulsivity challenge with the inter-trial-interval (ITI) extended from 5 s to 9 s (fITI-challenge). The primary read-outs for impulsivity (% premature responses), attention (accuracy) and other 5-CSRTT parameters were assessed in absolute terms (Fig. 1e, g, i) and also log-normalised to the within-subject value under vehicle (Fig. 1f, h, j; see Supplementary Tables 4–6 for statistical details of experiments in this cohort).

Under baseline conditions or in the attention-challenge, there was no effect of chemogenetic silencing of any of the three subregions on attention or impulsivity (Fig. 1e–h). But once impulse control was challenged by extension of the ITI, we observed a significant reduction of premature responding induced by chemogenetic inhibition of the ACC (*P* = 0.006, drug-group interaction, RM-ANOVA across all four groups and two within-subject conditions; *P* = 0.001, Sidak-test for CNO vs. vehicle within the ACC group; Fig. 1i) down to 29% of the vehicle value on average (*P* = 0.008, one-sample *t* test). This effect clearly dominated the response profile across groups and assessed 5-CSRTT parameters (Fig. 1j). Further, premature responding did not correlate with any of the other behavioural variables in the ACC group indicating a high specificity of this effect (Supplementary Table 6). Response latency and attentional accuracy were slightly, but significantly, increased by the same manipulation of the ACC, while omissions and the number of correct responses remained unaltered (Fig. 1j). This profile suggests that the anti-impulsive effect observed in the ACC-hM4Di group was not merely a side-effect of general sedation or blunting of responsiveness. We further established that none of the described effects reached significance at 3 mg/kg CNO or lower, that the anti-impulsive effect in the ACC-hM4Di group was reproducible with the alternative DREADD-agonist compound 21, and that repeated CNO-exposure during the task did not change later task performance (Supplementary Fig. 1).

Notably, chemogenetic inhibition of the PrL consistently increased the reward latency in all three paradigms (Fig. 1f, h, j). Furthermore, we found in the open-field LMA test that hM4Di-activation in all three subregions reduced novelty-induced locomotion (Supplementary Fig. 1). These findings confirm the efficacy of the chemogenetic modulation in all three regions.

### Anti-impulsive effect of G_i_-signalling in layer 5 cells of the ACC

**Fig. 2:**
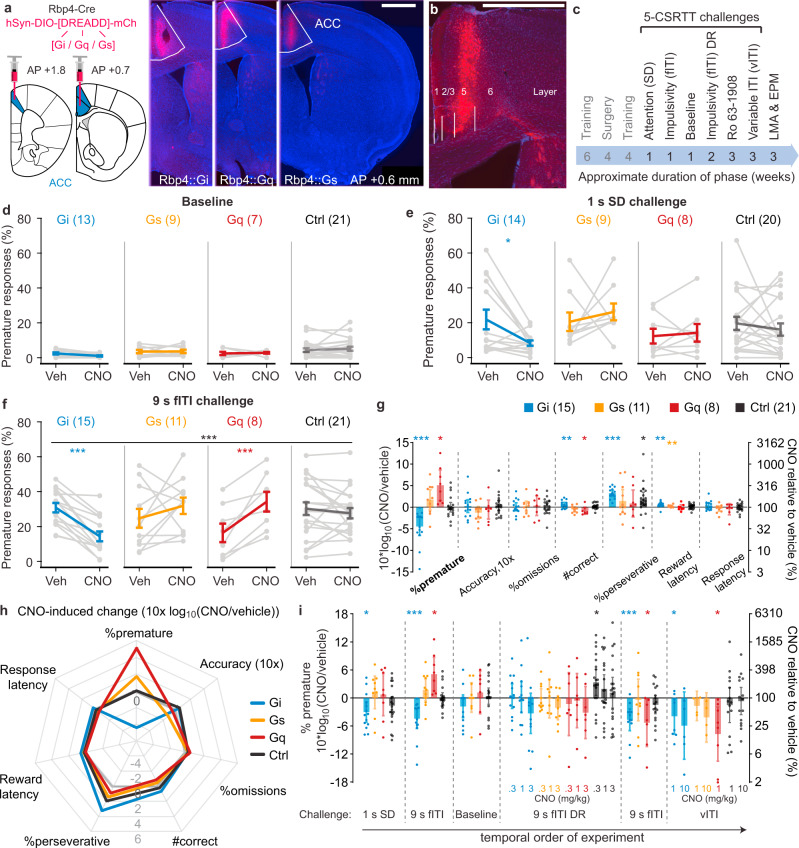
Activation of the G_i_-cascade in ACC L5-PCs reduces premature responding in parametric challenges of impulsivity. **a** Transfection (left) and Cre-dependent expression (right) of hM4Di-mCherry (G_i_), hM3Dq (G_q_) and rM3Ds (G_s_) in ACC L5-PCs in coronal slices collected at ~0.6 mm anterior of bregma; scale bar, 1 mm. **b** Laminar expression of hM4Di-mCherry (red); scale bar, 1 mm. **c** Temporal order of surgery and chemogenetic/behavioural testing, including the duration for each test phase; all tests except for the EPM were conducted within-subject with vehicle and CNO applications separated by 3-7 d. **d**–**f** Premature responses after injection of vehicle (Veh) or CNO for the four subgroups of the cohort [G_i_ (blue), G_s_ (orange), G_q_ (red), mCherry-controls (Ctrl; black), *N*-numbers in brackets] and 5-CSRTT challenge condition indicated above. Black stars indicate significant CNO-group interaction (RM-ANOVA); coloured stars indicate Sidak paired post-hoc test between vehicle and CNO conditions in the colour-coded group. **g** CNO-induced changes in the 9s fITI-challenge measured as log_10_-transform of the within-subject ratio (value after CNO/value after vehicle) for relevant behavioural parameters on the 5-CSRTT are shown for the 4 groups indicated in the legend with *N*-numbers stated in brackets. **h** Same data and *N*-numbers as in **g** depicting the parametric response profile of individual groups; grey line indicates 0-lattitude (no CNO-induced change). **i** CNO-induced changes of premature responses (as in **g**) shown for the different parametric challenges in order of their execution, including dose-response (DR) experiments in the fITI- and vITI-challenges as indicated. *N*-numbers for the first three protocols are stated in **d**–**f**, for the other three experiments *N*-numbers are as follows in chronological order; fITI-DR: G_i_ (13), G_q_ (7), G_s_ (9), Ctrl (20); repeated 9s-fITI: G_i_ (12), G_q_ (7), G_s_ (10), Ctrl (18); vITI: G_i_ (8 for 1 mg/kg, 6 for 10 mg/kg), G_q_ (8), G_s_ (4), Ctrl (15). Asterisks in **g** and **i** indicate one-sample *t*-tests against 0. See Supplementary Fig. 2 for related accuracy data and Supplementary Tables 3 and 7–9 for reasons for varying *N*-numbers across experiments, further statistical analysis, and absolute response numbers. 1 mg/kg CNO was used for the G_q_-group and a subset of Ctrl mice, 10 mg/kg was used for the remainder, except for DR experiments for which doses are indicated in **i**. *P < 0.05; **P < 0.01; ***P ≤ 0.001; error bars, s.e.m. in **d**–**f**, otherwise C.I.; individual dots or dot-lines represent subjects.

While the potential therapeutic effect of hM4Di-mediated modulation of *all* excitatory cell types in the ACC is encouraging, this cannot easily be translated into a molecular target given the diverse composition of this population. To narrow down the relevant cell-type, we specifically probed layer-5 pyramidal cells (L5-PCs), as they constitute the main output channel to other impulsivity-regulating brain regions (especially the striatum^13^) and are known to be modulated by many GPCRs^13,21^. To broadly assess the impact of GPCR-signalling in these ACC L5-PCs, we generated subgroups of Rbp4-Cre mice transduced either with mCherry or DREADDs for one of three major GPCR cascades, G_i_ (hM4Di), G_q_ (hM3Dq) and G_s_ (rM3Ds), each of which yielded the predicted laminar expression (Fig. 2a, b).

Following a similar experimental schedule as in the CamKIIα-Cre cohort (Fig. 2c), we first conducted the chemogenetic manipulation in the SD- and fITI-challenges, and the baseline condition. The results (Fig. 2d–g) were remarkably similar to those seen in the CamKIIα-Cre cohort: activating the G_i_-cascade in L5-PCs strongly reduced impulsivity induced by the fITI-challenge compared to both the vehicle condition within the same group and the CNO-condition in any of the other groups (*P* < 0.0005, drug-group interaction, RM-ANOVA; *P* < 0.0005 paired Sidak-test of vehicle vs. CNO within the ACC-G_i_ group; see Supplementary Tables 7–9 for statistics in the Rbp4-Cre cohort; Fig. 2f–h). Similar trends in the baseline-condition (affected by a floor effect, Fig. 2d) and the SD-challenge (Fig. 2e) did not reach significance at the level of an interaction (RM-ANOVA) but were apparent in the SD-challenge when analysing the ACC-G_i_ group alone (Fig. 2e, i). Acute activation of the G_i_-cascade in ACC L5-PCs did not alter impulsivity in the longer-term as animals that received CNO on their first challenge day showed a similar level of premature responding under vehicle as those animals that received vehicle first and CNO on the second test day (Supplementary Fig. 2).

Intriguingly—again in the fITI-challenge—the activation of the G_q_-cascade in L5-PCs had the opposite effect, namely a sharp increase in impulsivity (*P* = 0.001, paired post-hoc test of vehicle vs. CNO; Fig. 2f–h). The overall profile of CNO-induced effects diverged by far most sharply at the parameter of %prematures between the G_i_- and the G_q_-group: %prematures more than tripled within the G_q_-group, but were reduced to almost one third within the G_i_-group under CNO (Fig. 2 g, h). A similar antagonistic effect was seen for the total number of correct responses, which significantly increased in the G_i_-group (again, excluding the possibility of a sedative effect in this group) but decreased in the G_q_-group (Fig. 2g). The G_s_-group showed a qualitatively similar CNO-response profile as the G_q_-group, but did not reach significance (Fig. 2g, h). These observations suggested that L5-PCs of the ACC exert impulse control in a bidirectional manner, whereby a molecular antagonism between the activity of their G_q_- and G_i_-coupled receptors sets the level of waiting impulsivity of an animal.

However, further experiments to validate these effects revealed a more complex picture: already at a first repetition of the fITI-challenge within a dose-response experiment the pro-impulsive CNO-effect in the G_q_-group was no longer evident, and during subsequent fITI- and vITI-challenges, the activation of the G_q_-cascade in L5-PCs caused a significant *reduction* of premature responding, similar in extent as the activation of the G_i_-cascade evoked (Fig. 2i). This suggests that the repeated chemogenetic G_q_-activation in these cells during the challenges may lead to an adaptation process that fully inverts the behavioural consequence of this signalling mechanism. The activation of the G_i_-cascade in contrast, repeatedly produced an anti-impulsive effect across the tests, provided sufficient CNO doses (Fig. 2i).

**Fig. 3:**
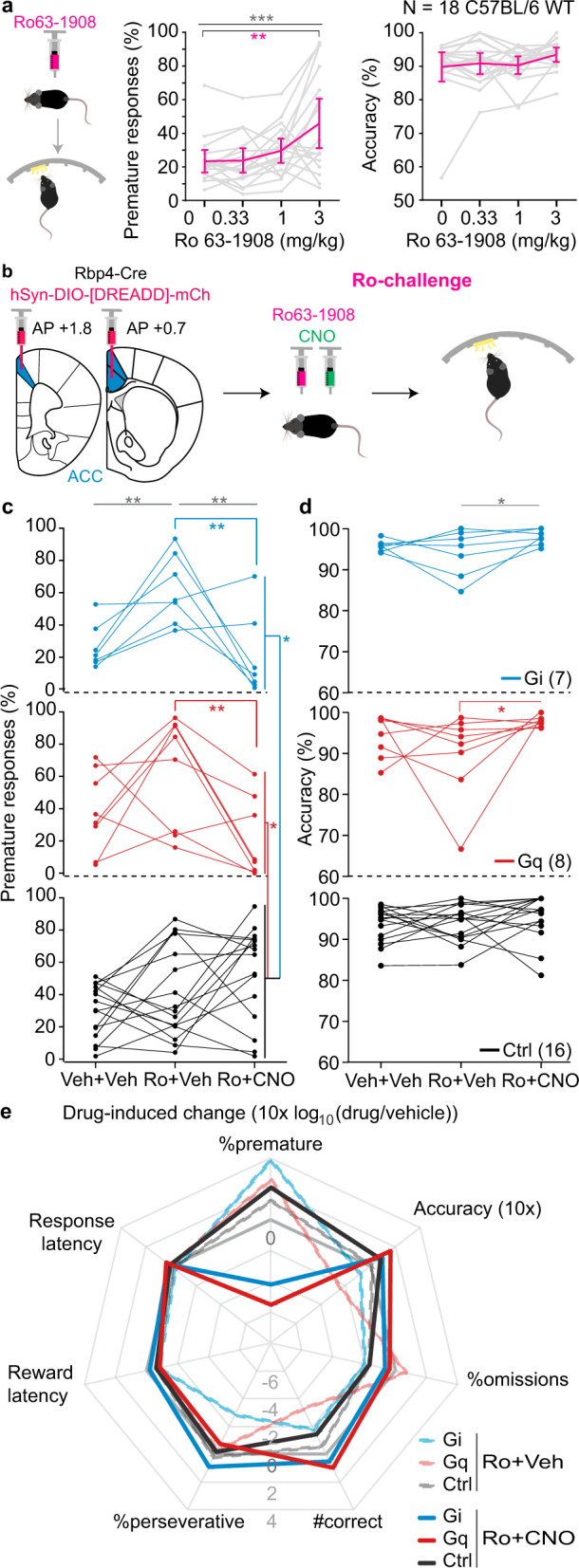
Activation of the G_i_-cascade in ACC L5-PCs reduces premature responding in a pharmacological challenge of impulsivity. **a** Premature responding and accuracy after injection of vehicle (0) or indicated doses of Ro 63-1903 (Ro) in a separate cohort of 18 wildtype mice. Error bars, C.I. **b** Experimental schedule applying Ro as impulsivity challenge and CNO  before testing in the 5-CSRTT in a subset of the DREADD-transduced Rbp4-Cre cohort evaluated in Fig. 2. %premature responses (**c**) and attentional accuracy (**d**) observed after application of 3 mg/kg Ro 63-1908 (Ro) and additional application of CNO (Ro+CNO). 1 mg/kg CNO was used for the G_q_-group and a subset of Ctrl mice, 10 mg/kg CNO was used for the remainder. In **a**, **c**, **d**, grey asterisks indicate significant main effect of drug condition across all groups (RM-ANOVA), coloured asterisks below horizontal lines indicate significant paired post-hoc comparisons between the indicated drug condition (vehicle and 3 mg/kg Ro in **a**, Ro+Veh and Ro+CNO in **c**, **d**) in the groups indicated by the colour (Sidak); coloured asterisks on the right indicate significant pairwise post-hoc comparisons between the group indicated by the colour (G_i_, blue; G_q_, red; Ctrl, black) at the Ro+CNO condition (Sidak). **e** Response profiles of Ro- (dashed lines of reduced opacity) or Ro+CNO-induced (solid lines) changes relative to the vehicle/vehicle condition measured as log_10_-transform of the within-subject ratio (value after drug/value after vehicle) for relevant behavioural performance parameters on the 5-CSRTT are shown for the three groups as indicated in the legend; grey solid line indicates 0-lattitude (no CNO-induced change). *N*-numbers for **c**–**e** stated in **d**. See Supplementary Tables 3 and 7–10 for reasons for varying *N*-numbers across experiments, further statistical analysis, and absolute response numbers, respectively. *P < 0.05; **P < 0.01; ***P ≤ 0.001; individual dot-lines represent subjects.

To further explore the possibility of chemogenetic anti-impulsive treatment, we established a *pharmacological* model of motor impulsivity. We found in a separate cohort of wildtype mice that 3 mg/kg of Ro 63-1908 (Ro), an antagonist of GluN2B-containing NMDA-type glutamate receptors (NMDARs), in combination with an extended 9 s fITI strongly and reliably increased premature responding (Fig. 3a, Supplementary Table 10), as previously shown in rats^22,23^. This model is further supported by the finding that the GluN2B-subunit, specifically, of NMDARs is over-expressed in the PFC of high-impulsive rats^24^. When assessing the anti-impulsive potential of G_i_- and G_q_-modulation in ACC L5-PCs in this pharmacological challenge of systemic GluN2B-antagonism, the same pattern emerged as seen with the previous parametric challenges (Fig. 3b). Analysing only the vehicle/vehicle and vehicle/Ro conditions, we found that Ro increased premature responding irrespective of group (*P* = 0.001 for effect of dose; *P* = 0.275 for dose-group interaction, RM-ANOVA). However, additional CNO (Ro/CNO condition) fully normalised this pharmacologically induced impulsivity in both the G_i_- and the G_q_-group, but not in mCherry-transduced controls (Ro/vehicle vs. Ro/CNO comparisons: *P*(G_i_) = 0.017, *P*(G_q_) = 0.010, *P*(Ctrl) = 0.454; vehicle/vehicle vs. Ro/CNO comparisons: *P*(G_i_) = 0.936, *P*(G_q_) = 0.313, *P*(Ctrl) = 0.013; Sidak-tests; Fig. 3c). The overall response profile suggested that neither of the chemogenetic modulations caused adverse effects in other variables of this task, as latencies remained similar or even decreased, and accuracy, omission rates, and number correct responses qualitatively—or even significantly—improved with CNO compared to the vehicle/vehicle condition (Fig. 3d, e; Supplementary Tables 7–9). In line with this interpretation, %premature within the G_i_-group was neither correlated to nor confounded by other 5-CSRTT behavioural parameters (Supplementary Table 9).

**Fig. 4:**
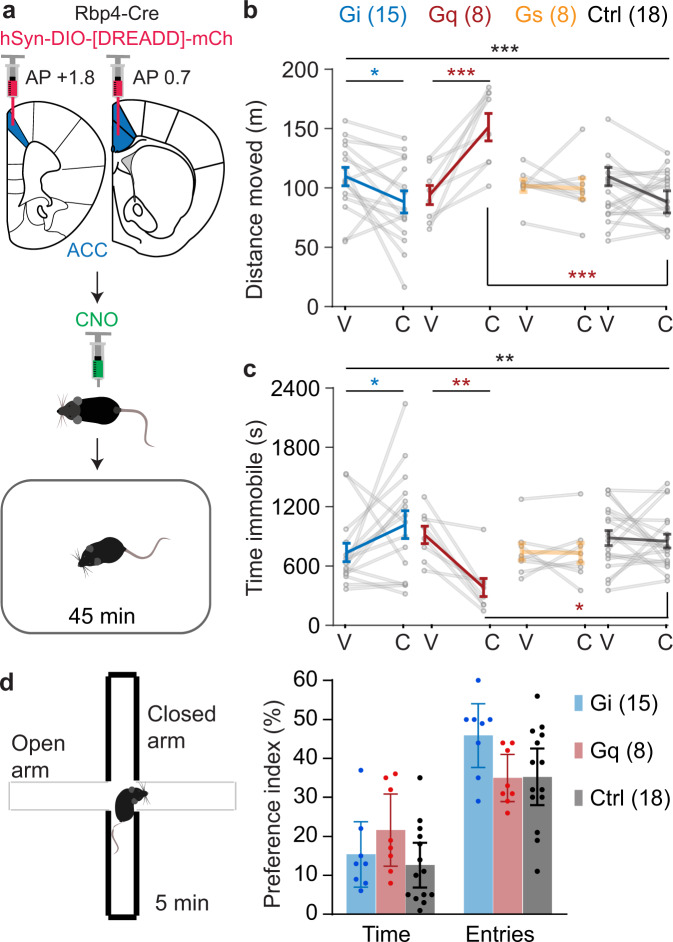
The G_q_- and the G_i_-cascades in ACC L5-PCs have antagonistic effects on locomotor activity. **a** Experimental set-up. Locomotor activity in a novel open-field assessed by total distance moved (**b**) and total amount of time spent immobile (**c**) during the 45 min test after injection of vehicle or CNO. Group identity and *N*-numbers stated in **b**. Black stars on top line indicate significant drug-group interaction (RM-ANOVA); coloured stars on horizontal lines below indicate a significant difference between vehicle and CNO conditions in the group coded by the colour (Sidak paired post-hoc test); coloured stars below data points indicate significant difference to the corresponding value in the Ctrl group (Sidak post-hoc test). **d** Exploration/anxiety ratio assessed by the preference to visit the open arm of an elevated plus-maze after injection of CNO. Group identity and *N*-numbers stated in colour legend. See Supplementary Tables 3 and 7 for statistics and reasons for varying *N*-numbers across experiments, respectively. 1 mg/kg CNO was used for the G_q_-group and a subset of Ctrl mice, 10 mg/kg was used for the remainder. *P < 0.05; **P < 0.01; ***P ≤ 0.001; error bars, C.I.; individual dots or dot-lines represent subjects.

Interestingly, during subsequent testing of novelty-induced LMA, the original CNO-induced divergence between the G_i_ and G_q_ groups re-appeared: CNO mildly, but significantly, reduced locomotion in the G_i_-group, but caused a comparatively strong increase of locomotor activity in the G_q_-group (Fig. 4a–c). Both effects were likely not mediated by increased exploratory drive (Fig. 4d). This re-confirms that only the G_i_- - not the G_q_- - cascade activation in ACC L5-PCs would consistently evoke the therapeutic profile needed in impulse control disorders.

### *Grm2/Grm3* are the most selectively expressed Gi-coupled GPCRs in murine ACC L5-PCs

**Fig. 5:**
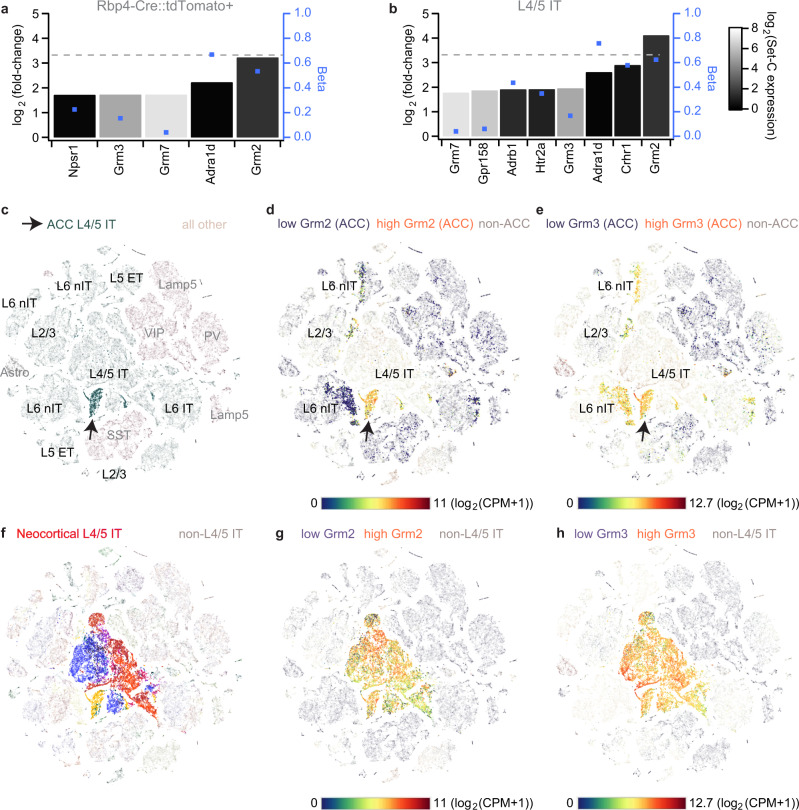
Differential gene expression analysis across 402 GPCR-encoding genes within the mouse ACC. **a, b** Differential gene expression values (log_2_-transformed ratios of gene expression in target set of ACC L5-cells relative to a non-target set of GABAergic, L2/3/6 excitatory and non-neuronal ACC cells, *N* = 3514; left axis), difference of share of cells *with* expression in target set and non-target set (Beta, blue dots, right axis), and average expression level in non-target set (log_2_(CPM + 1); grey scale) are displayed for all GPCR-encoding genes that are among differentially expressed transcripts in target set (Bonferroni-corrected *P* < 0.05) and are expressed at least threefold higher in target set compared to the non-target set. Horizontal dotted line indicates a 10fold higher expression in the target set (referring to left axis). The calculation has been performed by either selecting tdTomato-positive excitatory cells extracted from an Rbp4-Cre::Ai14 line (i.e. using the same selection as for the chemogenetic experiments in Figs. 2–4; *N* = 676; **a**), or by selecting clusters of L4/5 intertelencephalic-projecting (IT) cells according to the metadata (*N* = 1238; **b**). **c** t-SNE maps of cell-type clusters of the complete dataset^12^ involving 20 cortical regions with the cells from the ACC target (green, arrow) sets indicated; overlaid black and grey names indicate clusters of excitatory and non-excitatory cells, respectively. **d**, **e** Expression levels—colour-coded according to the continuous scale below—of *Grm2* (d) and *Grm3* (e) projected onto the ACC cells of the t-SNE map shown in **c** indicate high expression of those genes majorly in excitatory cells, with *Grm2* (compared to *Grm3*) showing a higher specificity for L4/5 IT cells. **f** Same plot as (c) but for displaying L4/5-IT cells across all analysed cortical areas (shown in different colours). Expression of *Grm2* (**g**) and *Grm3* (**h**) across L4/5–IT cells of all analysed regions. Gene expression analysis and creation of t-SNE maps have been performed in CytosploreViewer. See Supplementary Data 2 for details of sets, results and analysed GPCR genes, and Supplementary Fig. 3 for the same analyses as in **a**, **b**) using a reduced number of cell types for the non-target set.

The above data imply that G_i_-coupled receptors that are *endogenously* expressed in ACC L5-PCs with reasonable selectivity would present promising drug targets to reduce impulsivity in psychiatric diseases. Therefore, we sought to identify all GPCR-encoding genes that are strongly and selectively expressed in those cells using the largest available single-cell gene expression dataset for mouse ACC which also includes several hundred L5-PCs that have been collected by using the Rbp4-Cre driver line^12^. Calculating differential gene expression between ACC L5-PCs (either defined as Rbp4-Cre::tdTomato-positive or as L4/5-IT cells, Set-T) and all other non-L5 ACC cell types (Set-C, see ‘Methods’) we found five selectively expressed GPCR genes for the Rbp4 target-cell set and eight for the L4/5-IT Set-T, out of 402 GPCR genes surveyed (Fig. 5a, b). Of these, only the metabotropic glutamate receptor genes *Grm2/3* showed a low expression in Set-C, a large separation (Beta) between the two sets in *both* comparisons (Fig. 5a, b), *and* encode G_i_-coupled GPCRs^25^. Although not the most strongly expressed in absolute terms, *Grm2* was the only GPCR gene that was expressed around 10fold higher in Set-T than in Set-C across comparisons (Fig. 5a–e; Supplementary Fig. 3). Even though it is also expressed widely in L5-PCs of other cortical areas (Fig. 5f–h), *Grm2* represents the comparatively most promising GPCR-target to activate G_i_-signalling in L5-PCs of the ACC, according to this analysis.

In order to verify its suitability in humans, we used a large-scale dataset of 5939 single-cell transcriptomes of the human cingulate cortex to analyse differential expression of 399 non-sensory GPCR-encoding genes^12^. We found that *GRM2* and *GRM3* were still expressed at least three times higher in L5-PCs compared to a Set-C comprising GABAergic, non-neuronal and L6-cells. However, also other genes encoding G_i_-coupled receptors emerged that had not been identified by the analysis of the murine dataset but could constitute potential targets in humans, most notably HTR1F (Supplementary Fig. 4).

### Anti-impulsive action of an mGluR2/3 agonist and an mGluR2 potentiator

**Fig. 6:**
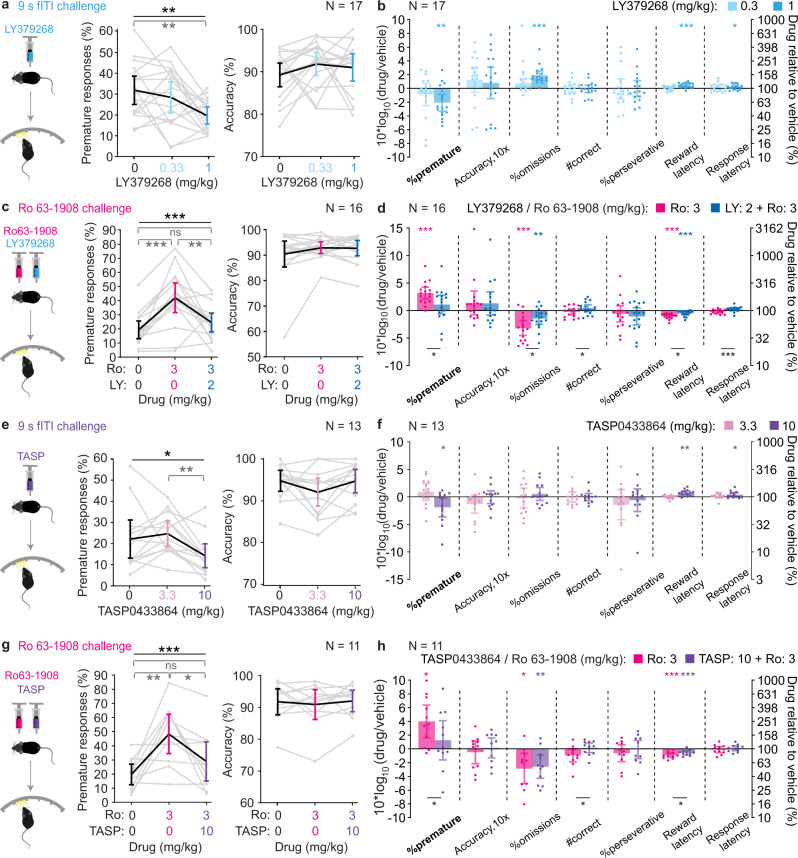
Reduction of impulsivity by the mGluR2/3 agonist LY379268 and the mGluR2-PAM TASP0433864. Absolute values of %premature responses (left) and attentional accuracy (right) after injection of vehicle (0) or various doses of LY379268 (LY; **a**, **c**) or TASP0433864 (TASP; **e**, **g**), as indicated, in the parametric 9s fITI (**a**, **e**) or pharmacological Ro 63-1908 (Ro, **c**, **g**) challenge conditions. The same group of 18 male C57BL/6 mice was used for every LY-experiment and the same group of 13 male C57BL/6 wildtype mice was used for every TASP-experiment with contributing *N*-numbers indicated in the accuracy panels. Black stars indicate significant effects of dose; grey stars indicate significant paired post-hoc comparisons between dose-levels (Sidak). (**b**, **d**, **f**, **h**) Drug-induced changes in the corresponding experiment identified in **a**, **c**, **e**, **g** measured as log_10_-transform of the within-subject ratio (value after drug(s)/value after vehicle) for relevant behavioural performance parameters on the 5-CSRTT are shown for the tested doses and drug combinations as indicated in the colour legend. Asterisks indicate one-sample *t*-tests against 0, colour-coded according to the dose. For the Ro-challenges (**d**, **h**), the results of paired *t*-tests between the two drug conditions are also indicated by black asterisks below bars. See Supplementary Tables 10, 11 (for LY) and 13-14 (TASP) for statistics, further analysis, and reasons for varying *N*-numbers across experiments. *N*-numbers are stated in each panel. *P < 0.05; **P < 0.01; ***P ≤ 0.001; error bars, C.I.; individual dots or dot-lines represent subjects.

The above analysis predicts that an agonist of the G_i_-coupled GPCRs mGluR2 and/or mGluR3 (encoded by *Grm2* and *Grm3*, respectively) reduces waiting impulsivity in mice. To confirm this prediction, we repeated our challenge protocols after acute pre-treatment with the mGluR2/3 agonist LY379268 in a new cohort of C57BL/6 wildtype mice trained in the 5-CSRTT. We found that 1 mg/kg LY379268 effectively reduced premature responding in both the fITI-challenge (Fig. 6a, b) and the SD-challenge, while 2 mg/kg was necessary in a combined attention/impulsivity challenge (Supplementary Fig. 5). Although LY379268 also increased omissions in these challenges, at a dose of 2 mg/kg it improved attentional accuracy suggesting that attention is not negatively affected (Supplementary Fig. 5).

To further benchmark this response profile, we assessed the effect of guanfacine—an agonist of the only cortically expressed G_i_-couped receptor that is currently used as direct target in the treatment of ADHD (α2A-adrenoreceptor)^26^—in the same challenge. Across doses, the guanfacine-induced increase of omissions and latencies was much more profound than what we obtained with LY379268, scaling proportionally with a decrease of premature responding (Supplementary Fig. 6). Moreover, bivariate correlations across individual mice revealed that increases of omissions and reward latency were strongly associated with guanfacine-induced decreases of premature responding, but not with LY379268-induced reductions of premature responding (1 mg/kg for each drug; Supplementary Fig. 6; Supplementary Tables 11, 12). Further, the number of correct responses decreased with increasing doses of guanfacine, but not LY, and across mice a decrease in premature responses even correlated with higher number of correct responses under LY, but not guanfacine (1 mg/kg; Supplementary Fig. 6; Supplementary Tables 11, 12). These overall patterns suggested a more specific anti-impulsive effect of LY379268 compared to guanfacine. LY379268 (2 mg/kg) also normalised Ro-induced impulsivity without adverse effects on other performance variables—in fact, here omission rates remained even significantly below the vehicle/vehicle condition (Fig. 6c, d, Supplementary Table 10).

Given that mGluR2 was considerably more confined to L5-PCs than mGluR3 (Fig. 5d, e), we wondered if a positive allosteric modulator (PAM) of this receptor^27^, like TASP0433864 (TASP)^28^, is sufficient to achieve an anti-impulsive effect. Using a separate cohort of 13 C57BL/6 mice (wildtype controls of the *Tacr1*-cohort used after the chemogenetic experiments described below), we found that 10 mg/kg TASP exerted a marginal reduction of %prematures in the fITI-challenge (*P* = 0.035, one-sample *t*-test) without increasing omissions (Fig. 6e, f). Furthermore, TASP pre-treatment significantly reduced Ro-induced waiting impulsivity without any unwanted side-effects (Fig. 6 g,h; Supplementary Tables 13, 14). These data confirm the prediction of our target discovery approach and suggest that mGluR2-PAMs could be potent anti-impulsive compounds.

### Chemogenetic gene therapy of increased impulsivity

**Fig. 7:**
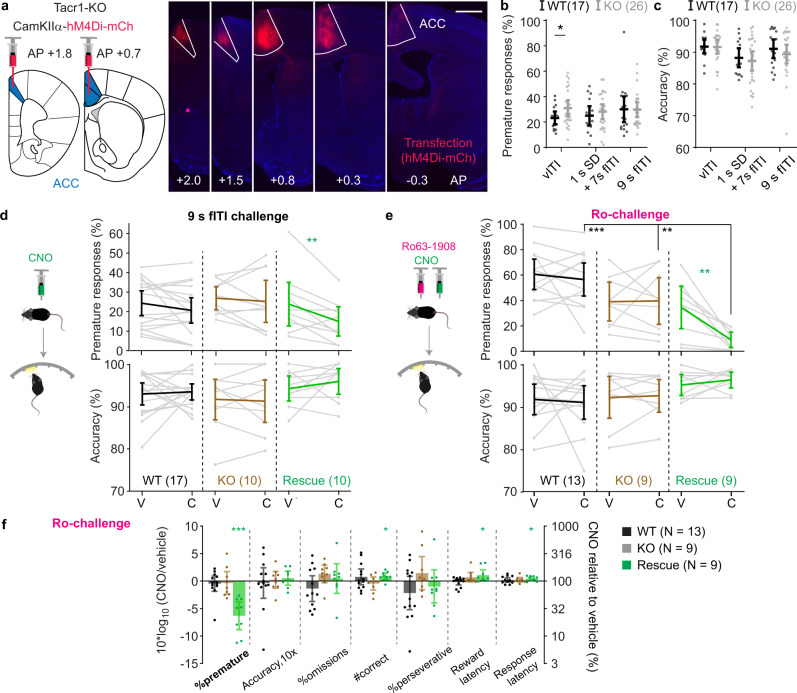
Activation of the G_i_-cascade in pyramidal cells of the ACC reduces premature responding in a pharmacological model of impulsivity. **a** Transfection of the ACC with a CamKIIα-hM4Di-mCherry vector (red) shown in different slices along the AP-axis (as indicated); scale bar, 1 mm. **b** Premature responding and **c** attentional accuracy in *Tacr1*-knockouts (KO, grey) and littermate wildtype controls (WT, black) in impulsivity-promoting challenges, as indicated: variable ITI (vITI), combined reduction of stimulus duration (1 s) and fixed-duration increase of ITI (7 s), and fixed ITI increase (9 s fITI). Note that only 15 WT participated in the vITI and combined challenge, only 25 KO participated in vITI and 9s-fITI challenge. **d**, **e** Premature responding and accuracy after injection of vehicle (V) or CNO (C) (**d**), and double-injections of Ro/Veh (V) or Ro/CNO (C) as indicated (**f**) during the vITI protocol in KO (brown), WT (black), or KO with hM4Di-transfection in the ACC (Rescue, green). Coloured asterisks indicate significant paired post-hoc comparisons (Sidak) within the indicated group; black asterisks on horizontal lines indicate significant within-drug difference between rescue and control groups (Sidak). Note that in **e**, premature responses, there is also a significant drug-group interaction and effect of group in overall RM-ANOVA, and a significantly lower impulsivity in both KO groups compared to WT under Ro/vehicle condition (Sidak), not indicated. **f** CNO-induced changes in the 5-CSRTT Ro-challenge measured as log_10_-transform of the within-subject ratio (value after CNO/value after vehicle) for relevant behavioural performance parameters on the 5-CSRTT are shown for the three groups as indicated in the legend; asterisks indicate one-sample *t*-test against 0. See Supplementary Tables 3, and 15, 16 for reasons for varying *N*-numbers across experiments, statistics, and absolute numbers of responses. *N*-numbers stated in each panel. Doses of 10 mg/kg CNO were used throughout. *P < 0.05; **P < 0.01; ***P ≤ 0.001; error bars, C.I.; individual dots or dot-lines represent subjects.

Given that the activation of the G_i_-cascade in all CamKIIα-positive ACC neurons improved behavioural parameters that correspond to the three cardinal symptoms of ADHD—impulsivity, inattention, hyperactivity—we also evaluated the potential of a direct hM4Di-based chemogenetic treatment approach^29^. We deployed a mouse line with homozygous knockout of the tachykinin 1 receptor gene (*Tacr1*^-/-^) because of its validity to model several aspects of ADHD, including (a) the three phenotypes stated above, (b) their responsiveness to several drugs approved for ADHD treatment, (c) reduced dopamine signalling in PFC and striatum, and (d) the genetic risk - given polymorphisms of *TACR1* in a subgroup of ADHD-patients^30–35^. The ACCs of 14 knockouts of a *Tacr1*^-/-^ cohort pre-trained in the 5-CSRTT were transduced with an AAV8-CamKIIα-hM4Di-mCherry vector (rescue group; Fig. 7a), whereas a further 13 knockout and 17 wildtype pre-trained littermate controls remained untransduced. After re-training, the impulsivity of the *Tacr1*^-/-^ mice was assessed in various challenges, of which only a variable ITI (vITI) condition produced elevated %prematures in knockouts compared to littermate controls (*P* = 0.046, *t*-test; Fig. 7b), which confirms previous studies^32,36^. Attentional accuracy was normal (Fig. 7c). However, increased premature responding was not apparent when the vITI-challenge was repeated with concomitant i.p. injections of vehicle (Fig. 7d), as previously observed^37^. Nevertheless, the hM4Di-transduced rescue group showed a significant CNO-induced reduction of premature responses as predicted (*P* = 0.006, Sidak-test; Fig. 7d; Supplementary Table 15).

Applying the pharmacological Ro-challenge to our *Tacr1*^-/-^ cohort, we found that hM4Di-activation in ACC excitatory cells profoundly reduced Ro-induced %prematures (*P* = 0.036 for drug-group interaction, RM-ANOVA; *P* = 0.002 for paired Sidak-test of Ro/vehicle vs. Ro/CNO within the rescue group; *P* < 0.01 for pairwise Sidak comparisons against the other two groups at the Ro/CNO condition; note that the Ro-challenge was also significantly less effective in both knockout-groups compared to the wildtype controls; Fig. 7e). This reduction of premature responding by 75%, on average, relative to Ro/vehicle was not produced by a general blunting of behaviour: the number of achieved correct responses was even increased by CNO within the rescue group (Fig. 7f). Thus, while elevated impulsivity caused by the *Tacr1*-knockout was too unstable^37^ to assess a chemogenetic rescue, the data gained in this cohort with the pharmacological challenge nevertheless suggests the possibility of a chemogenetic gene therapy in the ACC to moderate high motor impulsivity.

## Discussion

We here demonstrated the feasibility of a rational strategy for preclinical psychiatric drug target discovery by identifying the anti-impulsive action of G_i_-coupled receptors in L5 pyramidal cells of the ACC, in general, and of mGluR2, specifically. Strikingly, alternative GPCR targets were scarce.

Notably, the finding that the ACC controls impulsive action may be regarded as surprising since several previous studies rather linked the *IL* to premature responding in the 5-CSRTT, especially in rats^38–41^ (Supplementary Table 1). Various factors may explain this discrepancy, including variations of anatomical targeting and use of challenge protocols. For example, the—to our knowledge—only other study activating hM4Di in CamKIIα-positive ACC (Cg1) cells in this task, may not have noted this effect due to absence of an ITI-challenge^42^. Importantly, discrepancies between studies are less likely explainable by species-specificity, given that one lesion study each in rats^43^ and mice^44^ associated premature responding in the 5-CSRTT with the ACC. Also, rats bred for high trait-impulsivity show decreased GABA_A_ receptor binding specifically in the ACC^45^, which is in line with our finding that decreased excitatory activity in this brain region entails decreased impulsive action. The ACC has also been linked to impulsivity in humans^46,47^, and in adolescent subjects lower GABA levels in the ACC correlate with higher motor impulsivity^48^. These studies suggest that our findings on the key role of appropriate modulation of ACC excitatory cells in impulsivity translate to rats and humans.

The activation of G_i_-signalling in excitatory ACC-cells, including in L5-cells specifically, also caused a mild decrease of locomotion (Fig. 4b, c; Supplementary Fig. 1)—as would be beneficial for an ADHD-treatment. However, this effect alone cannot explain the profound anti-impulsive effect of the same treatment in the 5-CSRTT for multiple reasons. Firstly, a similar or stronger decrease of locomotion was observed with inhibition of pyramidal cells in PrL and IL as well, but these effects did not translate into reduced premature responding in the 5-CSRTT. Secondly, the locomotor-decrease was much smaller relative to the decrease in impulsivity (compare Fig. 4b with Fig. 2f). Thirdly, and most importantly, if a blunt decrease of locomotor drive was responsible for the decreased premature responses, then this should also lead to an equivalent decrease of other actions, especially correct responses, and a concomitant increase of omission rates and reward latency (as observed with guanfacine, Supplementary Fig. 6). This was, however, not the case; partly, correct response numbers even increased (Figs. 1, 2). Further, ANCOVA and correlation analyses proved the independence of premature responding from locomotor-related variables in the G_i_-groups in the 5-CSRTT (Supplementary Tables 6, 9, 15). Also, the Rbp4-Cre::G_q_ group demonstrates the possibility of a decoupling of impulsivity-related from locomotor-related effects of ACC-manipulations (negative correlation; Fig. 4b vs. Fig. 2f). These observations suggest that the anti-impulsive efficacy of the activation of G_i_-signalling in ACC L5-PCs is not mediated by locomotion-decreasing effects.

A further concern of our chemogenetic approach is unspecific effects of CNO, especially at the high doses used for most experiments of this study (10 mg/kg). Indeed, in individual experiments from our test batteries, CNO marginally *increased* (CamKIIα-cohort, 9 s fITI challenge; *P* = 0.062, one-sample *t*-test of log-ratio against 0) or *decreased* (*Tacr1*-cohort, vITI-challenge; *P* = 0.049) premature responding in control subjects—with inverse effects on response latency (*P* < 0.05; Supplementary Fig. 8). Other 5-CSRTT parameters were also affected by CNO in control groups occasionally, namely perseverative responses (Rbp4-Cre cohort, 9 s fITI challenge), reward latency (vITI challenges in Rbp4-Cre and *Tacr1* cohorts), and the number of correct responses (CamKIIα-cohort, 1 s SD challenge; *P* < 0.05, Supplementary Fig. 8). Importantly however, none of these effects appeared *consistently* across cohorts or across challenges within the same cohort—in line with our previous study on CNO effects in the 5-CSRTT^20^. Nevertheless, complex interactions between transfected region (i.e. unspecific effects of mCherry expression), CNO dose, challenge condition, and prior behavioural or pharmacological experience could still exist, that lead to an efficacy of CNO under certain conditions. For example, in Rbp4-Cre controls, we observed a qualitative inverse dose-response relationship with a significant increase of impulsivity by 0.3 mg/kg CNO that disappeared with higher doses (Fig. 2i). Moreover, our mixed control groups – featuring an imbalance between transduced PFC sub-regions (e.g. a lack of specific PrL controls and a majority of ACC controls in the CamKIIα-cohort) or genotype (more Cre-negative than Cre-positive mice in the Rbp4-Cre cohort)—hamper a more systematic analysis of interactions between unspecific CNO effects and mCherry-transfections in specific cells or regions. There were no significant differences between the subgroups of these control groups (Supplementary Fig. 9) which renders such unspecific effects to be an unlikely scenario. However, the general variability observed across animals underscores the importance of both multiple replications of challenge experiments and the systematic incorporation of within-subject vehicle control data in addition to between-subject controls. Rather than individual observations of chemogenetic modulation effects in individual experiments, it is the consistency with which we observed specific anti-impulsive action of G_i_-modulation of ACC excitatory cells across multiple impulsivity challenge protocols and across distinct cohorts that lent validity to our results.

The identification of mGluR2/3 as anti-impulsive GPCR targets is in line with previous pharmacological studies. For example, LY379268 may reduce seeking and self-administration of methamphetamine^49^ and alcohol^50^ in rats. Similarly, systemic administration of LY379268 could improve deficits of accuracy and waiting impulsivity induced by intra-PFC infusion of an NMDAR-antagonist in the 5-CSRTT in rats^51^. Furthermore, both systemic application of 1 mg/kg LY379268^52^ and its local infusion into the PFC^53^ were shown to selectively reduce elevated premature responding in the 5-CSRTT induced by systemic^52^ or prefrontal^53^ application of the 5-HT2A/C agonist DOI in rats, respectively. The latter suggests that *prefrontal* mGlu2/3 receptors may indeed mediate the anti-impulsive effect of their agonist. However, neither these nor our data are sufficient to rule out the possibility that mGluR2/3 activation in IT-cells of other cortical areas (Fig. 5f–h) or other brain structures like the nucleus accumbens^54^ may contribute to its anti-impulsive action. Prefrontal mGluR2/3 may be sufficient, but not necessary for the anti-impulsive effect of their agonist. The clarification of this question requires cell-type and area-selective ablation of *Grm2/3* before drug application. In our current study, the replication of the anti-impulsive effect of LY379268 in our 5-CSRTT-challenges in mice served as a first validation of the introduced target-discovery approach by confirming its primary prediction. It further allowed to go beyond the previous studies by demonstrating that positive allosteric modulation of the more selectively expressed mGluR2 alone (Fig. 5d, e) is sufficient to reduce impulsivity, in line with the mGluR2-PAM-induced reduction of response rates in a *differential reinforcement at low-rate 72* task seen previously in rats^55^. The availability of two mGluR2-PAMs^56,57^, an mGluR2-agonist prodrug^58^, and an mGluR2/3 agonist prodrug^58–60^ that are safely applicable in humans can enable the direct translation of this pre-clinical evidence into clinical trials using human analogues of the 5-CSRTT. Our combined chemogenetic and gene expression data now supply a possible mechanistic explanation for the propensity of mGluR2/3-activation to reduce impulsive action and suggest a surprising absence of likely alternatives among prefrontal GPCRs. Both aspects may motivate the further clinical evaluation of mGluR2 in impulse control disorders, including ADHD and addiction.

Our study also reveals two important and rather generic caveats of attempts of pre-clinical drug target discovery in psychiatry. Firstly, as we surveyed 402 murine and 399 human GPCR genes, the limited number of identified suitable GPCRs, the absence of highly differentially expressed orphan GPCRs, and the broad expression of the *identified* GPCRs across other brain regions showcase the surprisingly narrow limits of finding novel and very specifically expressed GPCR targets in biological psychiatry, as far as the predictive power of the murine model is concerned. This finding is unrelated to our focus on impulsivity, but simply due to the rather similar GPCR expression patterns across cortical cell-types and similar gene expression patterns across related cell-types in distinct areas^12,13^, although a similar analysis of sub-cortical circuits may yield more specific targets, in future. Secondly, our comparative analysis of the same target cells of the *human* cingulate cortex demonstrated several differences in the expression of suitable target genes compared to mice, even though a very narrowly defined cell-type was analysed. This finding demonstrates that the level of the cellular signalling cascade (G_i_ in ACC L5-PCs, in our case) is the more relevant level of target discovery than the concrete receptors found in mice—e.g., according to our analysis, HTR1F might be a more suitable receptor in humans to trigger G_i_-signalling in ACC L5-PCs than mGluR2, but will likely be difficult to evaluate in rodents.

In summary, our study demonstrates not only a viable approach for rational drug target discovery for psychiatry, but also illustrates the necessity to take a top-down perspective by surveying all possible targetable modulators of a symptom domain at multiple biological levels.

## Methods

### Animals

In total, 67 male B6.Cg-Tg(Camk2a-cre)T29-1Stl/J (CamKIIα-*Cre*, obtained from The Jackson Laboratory, Maine, US)^61^, 96 male B6.FVB(Cg)-Tg(Rbp4-cre)KL100Gsat/Mmucd (Rbp4-*Cre*, obtained originally from GENSAT, US), 18 male C57BL/6J wildtype mice, as well as 27 Tacr1^tm1Sph^ mice (*Tacr1*^-/-^) and 17 wildtype littermates of both sexes—all obtained from breeding of heterozygous *Tacr1*^+/-^ parents^62^—were used for this study. Animals were group-housed in Type II-Long individually ventilated cages (Greenline, Tecniplast, G), enriched with sawdust, sizzle-nest^TM^, and cardboard houses (Datesand, UK), and maintained at a 13 h light/11 h dark cycle. Mice started training in the 5-CSRTT at 2–3 mo of age, and were kept under food-restriction at 85-95% of their average free-feeding weight which was measured over 3 days immediately prior to the start of food-restriction at the start of the behavioural training. Water was available ad libitum. CamKIIα-*Cre* mice were genotyped for *Cre* using primers 5′-GCGGTCTGGCAGTAAAAACTATC-3′ and 5′-GTGAAACAGCATTGCTGTCACTT-3′ (100 bp product), and for the wildtype locus using primers 5′-CTAGGCCACAGAATTGAAAGATCT-3′ (324 bp product). Rbp4-*Cre* mice were genotyped for *Cre* using primers 5′-CACCCTGTTACGTATAGCCG-3′ and 5-GAGTCATCCTTAGCGCCGTA-3′ (330 bp product). *Tacr1*^-/-^ mice were genotyped for the wildtype locus using primers 5′-TGCCTGCTTGCCGAATATCA-3′ (270 bp product), and for the mutated (knockout) locus using 5′-GATCTCTTCCCCAACACCTCC-3′, and 5′-GAGGCCGTAGTACCACACG-3′ (detecting the neomycin resistance cassette of the disrupting insert, 357 bp product). All experiments were performed in accordance to the German Animal Rights Law (Tierschutzgesetz) 2013 and were approved by the Federal Ethical Review Committee (Regierungsprädsidium Tübingen) of Baden-Württemberg, Germany (licence number TV1344).

### Behavioural procedures

Behavioural testing was done blind to the subgroup identity of the mice. Mice started training in the 5-CSRTT at 2–3 mo of age, and were kept under food-restriction at 85-95% of their prior average free-feeding weight. Behavioural training and testing in the 5-CSRTT, elevated-plus-maze (EPM) and open-field test of novelty-induced locomotor activity (LMA) were conducted as preciously described^63^. Some mice did not contribute data to individual experiments for various reasons stated in Supplementary Table 3.

#### 5-CSRTT

Testing was conducted in operant chambers placed individually in melamine-MDF sound-insulated and ventilated outer boxes and fitted internally with an array of five nose-poke holes on one wall and a reward receptacle on the opposite wall. All six apertures could be illuminated to instruct the entry into them, and were fitted with IR break-beams to detect entry and exist of the animal’s snout. All experiments in the CamKIIα-*Cre* and Rbp4-*Cre* animals were conducted in a set of eight rectangular commercial operant chambers (ENV-307A-CT with ENV-115C-A 5-choice wall; Med Associates, VT, US) controlled by custom-written scripts through the Med-PC control software. All experiments in the *Tacr1*-KO and C57BL/6 wildtype cohorts were conducted in a set of 20 custom-made trapezoidal chambers based on the pyControl system^64^ (https://pycontrol.readthedocs.io; electronics components obtained from the European OpenEPhys store, Portugal, https://open-ephys.org/pycontrol).

The 5CSRTT training protocol was identical for all cohorts and similar to what we previously described^63^. In brief, after initiation of food-restriction to maintain animals at 85–95% free-fed baseline weight, mice were accustomed to consume the reward (strawberry milk, Müllermilch^TM^, G) first in their home cage, and then in the operant box (2–3 exposures each). The nutritional composition of this reward was 1.4% fat (0.9% saturated), 11.6% carbohydrates (all of which is sugar), and 3.2% protein, altogether providing 303 kJ energy per 100 ml. The standard diet that the mice otherwise received contained more energy (1400 kJ/100 g), fat (4.5%), protein (22%) and carbohydrates excluding sugar (34.2%), but less sugar (5.1%). We showed in a separate cohort that the milk reward could be devalued by pre-feeding and therefore is likely not hyper-palatable or addictive (Supplementary Fig. 7). Subsequently, mice were trained in 10–16 sessions (30 min, once daily) of habituation training. In each trial, all holes of the 5-poke wall were illuminated for an unlimited time and the mouse could poke into any one of them to earn a 40 μl milk reward subsequently disposed from the illuminated receptacle. If mice attained at least 30 rewards each in two consecutive sessions or (in exceptional cases) had reached the 16th session of habituation training, they were moved to the 5-CSRTT training, during which mice transitioned through five stages of increasing difficulty, based on reaching certain performance criteria in each stage (Supplementary Table 2). The difficulty was determined by the length of time the stimulus was presented (stimulus duration, SD) and the length of waiting time between the end of the previous trial and the stimulus presentation of the next trial (inter-trial-interval, ITI). In case a reward was collected on the previous trial, the ITI was initiated by the removal of the snout of the animal from the reward receptacle. In all 5-CSRTT protocols (Fig. 1c) only one pseudo-randomly selected aperture of the 5-choice wall was lit up after the ITI, indicating that this hole needs to be poked into (correct response) in order to earn a 20 μl milk reward. Trials were not rewarded but instead terminated immediately with a 5 s time-out period during which the house light was turned off, if the animals either poked into any hole during the ITI (premature response), poked into a non-illuminated hole (incorrect response) during the SD and limited-hold time (LH, until 2 s after SD), or failed to poke throughout the trial (omission). The relative numbers of such response types were used as performance indicators measuring premature responding [%premature = 100*(number of premature responses)/(number of trials)], sustained attention [accuracy = 100*(number of correct responses)/(number of correct and incorrect responses combined)], and lack of participation [%omissions = 100*(number of omissions)/(number of trials)]. In addition, the relative number of trials in which the mouse repeated the poke into the correct hole (perseverative responses) was measured as an indicator of perseveration [%perseverative = 100*(number of correct responses with a subsequent perseverative response)/(number of correct responses)]. Note that a trial was considered to start at the beginning of a new ITI, i.e. included premature responses. Also, the time required to poke into the indicated hole after it was illuminated (response latency) and the time from the exit from the correct hole until the entry into the reward receptacle (reward latency) were measured, whereby the latter is usually used as a compound indicator of motivation and locomotor drive^10^. In all stages and tests, sessions lasted 30 min and were performed once daily at the same time of day and in the same box for each animal.

#### Novelty-induced locomotor activity (LMA)

For measurement of LMA, mice were placed into a novel clear plastic cage (425 × 266 × 185 mm; Eurostandard Typ III, Tecniplast, G) filled with clean sawdust 10 min after application of CNO or vehicle, and left to explore for 45 minutes. CCTV cameras (Sentient, UK) installed centrally above the open-field cage were used to monitor each animal. Video-recordings from eight cage-stations were assembled into a single image frame through a CCTV-system (Dahua Inc, China), digitised through an A/D converter (TheImagingSource, G), and fed into ANY-maze (San Diego Instruments, USA), for video-tracking of movement. The total distance travelled was extracted from ANY-maze for the whole time of the experiment. Three to five days of wash-out were given between the first and second run. For the second run, brown translucent plastic cages of the same dimensions and a different spatial location for the open-field cage were used to enhance the novelty of the environment.

#### Elevated plus maze (EPM)

In order to assess exploratory drive in relation to unconditioned anxiety, an EPM made from grey plastic was used at ambient light levels of 80 lux in the centre of the maze. The +-shaped maze had two opposite closed arms and two open arms (35 cm long, 7 cm wide), elevated 75 cm above the ground. After 5–6 min of habituation in a novel cage in the test room, mice were placed in the centre of the maze facing an open arm and allowed to explore freely for 5 min. The position of the animal was tracked using a CCTV camera (Sentient, UK) and ANY-maze (San Diego Instruments, CA, US). The preference for the open arms was calculated as the ratio of the total time in open arms and the time spent on all arms combined (disregarding the time spent in the centre). Zone transition was determined by the position of the centre of the animal.

### Chemogenetic modulation and pharmacology

All compounds applied during behavioural experiments were delivered by either s.c. (Ro 63-1908) or i.p. (all other) route at an injection volume of 10 μl/g mouse. For chemogenetic manipulations, the DREADD agonist clozapine-N-oxide (CNO) or saline vehicle were injected i.p. 20 min (5-CSRTT), 10 min (LMA) or 25 min (EPM) prior to the start of testing. Except for the EPM which was done in a between-subject design (all mice receiving CNO), all chemogenetic and pharmacological experiments were conducted with vehicle and one or multiple agonist doses as within-subject conditions distributed in a latin-square design counter-balanced within each subgroup across test days. Types of vehicle, route of administration, and timing of injection are listed in Supplementary Table 17 for all compounds used in this study. In line with similar prior studies in mice^20,42^, 10 mg/kg was used as the default dose for CNO for hM4Di-, rM3Ds-, and most mCherry-transduced mice. For 8 of the 21 mCherry-transduced Rbp4-Cre mice and for all hM3Dq-transduced animals 1 mg/kg CNO was used for animal welfare reasons, because we observed seizures in two mice of a small pilot-cohort of hM3Dq-transduced CamKIIα-Cre mice with 10 mg/kg, likely due to unilateral off-site expression in secondary motor cortex (M2) adjacent to the ACC. The absence of consistent effects of CNO treatment on 5-CSRTT parameters in the control groups of the cohorts used for chemogenetic experiments in this study is documented in Supplementary Fig. 8. Chemogenetic experiments were usually followed by 1–2 wash-out days without training and 1–2 days with training before the next test (mostly conducted on Tuesdays and Fridays). Pharmacological experiments, in contrast, were usually conducted once per week. Given their large sizes the CamKIIα-Cre and Rbp4-Cre cohorts were split into four batches that were run separately.

#### Surgical procedures

After all mice reached at least stage 4 of the 5-CSRTT, they were assigned to one of the respective groups based on their performance over the first 3 days of this stage as a measure of counter-balancing. Animals were anaesthetised using isoflurane (AbbVie, G), received s.c. injections of analgesics (0.08 mg/kg buprenorphine, Bayer, G; 1 mg/kg meloxicam, Boehringer Ingelheim, G), and local scalp anaesthesia (200 μl of 0.025% bupivacaine, AstraZeneca, UK) before placement in a stereotaxic frame (motorised and atlas-integrated frame, Neurostar, G and Kopf, US; manual digital frame, World Precision Instruments, US) with non-rupture mouse ear bars. The body temperature was stabilised using a feedback-controlled heating blanket (Harvard Apparatus, US) and the anaesthesia was maintained with 1.5% isoflurane. The following stereotaxic coordinates (from bregma) and volumes were used for bilateral transfection of the stated areas; ACC: posterior injection at AP + 0.7, ML 0.3, DV 1.65 (200 nl) and 1.1 (300 nl), anterior injection at AP 1.8, ML 0.25, DV 1.25 (80 nl). PrL: posterior injection at AP + 1.9, ML 0.3, DV 2.25 (60 nl) and anterior injection at AP 2.4, ML 0.3, DV 1.95 (60 nl). IL: AP + 1.8, ML 0.3, DV 2.8 (50 nl). All viral vectors were of serotype AAV8 and were obtained from the University of North Carolina vector core (UNC, NC, US) or the University of Zürich viral vector facility (UZH-VVF, CH). Suspensions were diluted down to a final titre of 2.9*10^12^ vg/ml in 5% sorbitol/PBS (Sigma, G), wherever the original titre was higher. All *Cre*-dependent vectors were equal in backbone sequence and differed only with respect to its DREADD-mCherry fusion insert and were based on the pAAV2-hSyn-DIO-DREADD-mCherry-WPRE-hGHpA Addgene constructs 44361 (hM3Dq), 50458 (rM3Ds), 44362 (hM4Di), and 50459 (mCherry) from the Bryan Roth laboratory (https://www.addgene.org/Bryan_Roth/); 16 of the 21 Rbp4-Cre::mCherry control mice were actually *Cre*-negative but used anyway as a measure of *reduction* of animal usage (3R), and were therefore infused with a CamKIIα-mCherry-WPRE-hGHpA vector (v199-8, UZH-VVF), instead of the corresponding DIO-vector. Impulsivity of *Cre*-positive and *Cre*-negative animals did not differ (Supplementary Fig. 9). Likewise, for the CamKIIα-Cre cohort, a merged group of mCherry-transduced controls was used containing majorly mice with mCherry-transduced ACC and a minority of mice with mCherry-transduced IL; these groups were not different in premature responding (Supplementary Fig. 9). For the chemogenetic rescue approach in the *Tacr1*-knockout cohort a CamKIIα-hM4Di-mCherry-WPRE-hGHpA (Addgene construct 50477; v102-8, UZH-VVF) was used at the lower titre of 1.3*10^12^ vg/ml, as we observed very strong expression at its original titre 2.6*10^12^ vg/ml in pilot experiments. A pouch to absorb the virus was created by moving the needle 0.05 mm further down and then up again to the actual DV position before infusion. Infusions were made using a glass 10 μl precision syringe (WPI) at an injection rate of 100 nl/min. Upon completion of infusion the needle was kept in place for 5 min, moved up 0.1 mm and kept in place for another 5 min in order to minimise backflow of the virus. Mice received post-operative monitoring for 7 d, and an s.c. injection of the analgesic meloxicam (Metacam, 1 mg/kg, Boehringer Ingelheim, G) on the first 3 d. The mice were kept on ad libitum food for a minimum of two week before training in the 5-CSRTT commenced.

#### Histology

After the behavioural test battery, animals were given an over-dose of ketamine/medetomidine (≥200 mg/kg ketamine, Zoetis, G; ≥2 mg/kg medetomidine, Pfizer, US) and perfused with 0.01 M phosphate-buffered saline (PBS) followed by 4% PFA/PBS. The brains were rapidly removed and then stored in 4% PFA/PBS overnight before placement in 20% sucrose for dehydration before sections were cut at 60 μm thickness on a vibratome (VT1000, Leica, Deerfield, IL, USA). Every second section was stained with DAPI (10^−4^% w/v) for 30 min, washed with PBS twice and mounted on glass slides. A Leica DM6B epifluorescence microscope (Leica, G) was used to scan the slides with a ×5 objective and determine virus expression offline. Animals were only included in the datasets if they showed bilateral expression in the majority of the volume of the target structure but not in any other brain region. While minor and unilateral off-site expression in a neighbouring PFC subregion was tolerated, unilateral expression in M2 or M1 also led to exclusion of the animal given that this could potentially affect motor activity rather directly. mCherry-transduced control animals were not excluded based on expression patterns.

### Gene expression analysis

Differential gene expression analysis between L5-PCs and other ACC cells was performed using the mouse *anterior cingulate area* (ACA, 5190 cells) and the human *cingulate cortex* (CgC, 5939 cells) gene expression datasets from the Allen Institute for Brain Science^12^ (available at https://portal.brain-map.org/). For the mouse dataset, to represent our target cells (Set-T) we selected either all ACC cells that were pulled based on fluorescence in the Rbp4-Cre::Ai14(tdTomato) line in the original study (Rbp4 set; *N* = 676) or those cells that were classified as L4/5 inter-telencephalic projecting (IT) cells based on gene expression (L4/5-IT set; *N* = 1238), as this fraction innervates striatal regions^12,13^ which are key regulators of impulsivity in the brain^1^. 502 cells were part of both sets. As contrast sets (Set-C), in which the expression of a potential target gene should be particularly *low*, we included either only ACC GABAergic cells (least conservative), or added non-neuronal cells and L6 glutamatergic neurons (which might also exert inhibitory influence over excitatory cells of other layers^65,66^), or even added L2/3 cells (most conservative set). More details on the composition of the cell lists are included in the description of the Supplementary Data 2.

Using the Cytosplore Viewer (https://viewer.cytosplore.org), an online tool designed to analyse the SmartSeq gene expression dataset of the Allen Institute for Brain Science^12^, the 20 area dataset was opened and the cell lists of Set-T and Set-C loaded into *Selection Sets* 1 and 2, respectively, for calculation of differential gene expression for each of the six comparisons. Using a custom-written script in IgorPro 6 (WaveMetrics Inc., OR, USA), provided as Supplementary Code, gene expression parameters for each of the 402 GPCR encoding transcripts that were assessed in the dataset (see Supplementary Data 2) were extracted from the analysis results from CytosploreViewer, including:

(1) log_2_(1+CPM(exons+introns)), the log_2_-transform of the average expression of a gene (GE) across the cells of each set (log_2_(GE_Set-T/C_)), with *counts per million* (CPM) as normalised read count to measure gene expression (GE_Set-T/C_); 1 is added so that the expression value becomes 0 if the CPM value is 0.

(2) log_2_(fold-change) = log_2_(GE_Set-T_/GE_Set-C_) = log_2_(GE_Set-T_) – log2(GE_Set-C_), as primary measure of differential gene expression (Diff_Mean).

(3) Beta = s * |pct(Set-T) – pct (Set-C) | , pct measuring the *percent of cells* in each set expressing the respective gene at a threshold value of 1 and *s* being the sign (+/-) of the difference in average gene expression (Diff_Mean).

(4) *P*-value assessing the significance of the difference in gene expression between the two sets according to the Wilcoxon test, corrected for the number of comparisons using the Bonferroni-correction.

These four measures were used to assess differential GPCR gene expression according to the following rationale: putative target genes should be expressed significantly different in Set-T (layer 5 cells) compared to Set-C (Bonferroni-corrected *P* < 0.05), the difference (Diff_Mean) should be positive and correspond to an at least threefold higher normalised read count (GE_Set-T_/GE_Set-C_ > + 3; log_2_(fold-change) > 1.58). For GPCR-encoding transcripts that fulfil these criteria the key measures log_2_(fold-change), Beta, and the absolute expression in the contrast Set-C (which should be close to 0) were evaluated to identify the most suitable candidates (Fig. 5 and Supplementary Fig. 3).

To confirm the expression of identified targets in human L5 cingulate cortex cells, we followed a similar approach, based on CytosploreViewer, although cells had to be extracted purely based on clusters derived from location and gene-expression (due to absence of fluorescent reporter genes). Used clusters are stated in the corresponding figure legend of Supplementary Fig. 4 and listed in Supplementary Data 2.

### Statistics and reproducibility

Behavioural data was analysed using SPSS 26.0 (IBM, NY, US) and only two-sided tests were used. All within-subject non-normalised data from each experiment (one challenge within one cohort) was analysed with repeated-measures ANOVAs involving the group and the drug dose as between- and within-subject independent variables and one of the behavioural parameters as independent variables. In case of a significant effect of group, dose or interactions, pairwise between-subject and/or paired within-subject Sidak-adjusted simple main-effects post-hoc tests were conducted, as appropriate, across all groups and/or doses. Data from different experiments (challenges) were not combined for any ANOVA. In addition, within-subject chemogenetic and pharmacological data was normalised to the corresponding value under vehicle condition and the resulting (drug-value/vehicle-value) ratio was log_10_-transformed to allow parametric statistical comparisons which were conducted with one-sample *t* tests against 0 and, for pharmacological data, paired sample *t*-tests. For the two parameters that could occasionally assume the value 0% (%prematures, %perseveratives) the actual value was rounded up to the next full integer (i.e. values <1% were set to 1%, values ≥1% and <2% were set to 2%, etc.) before log-transformation, throughout all analysis, to avoid distortions of the data by values <1%, which are biologically insignificant. Dependencies between variables were assessed by including individual, possibly confounding variables as covariates in the repeated-measures ANOVA (ANCOVA) and by bivariate *Pearson’s* correlations. All *N*-, *p*-, *r-* and F-values for all analysed measures in each challenge and cohort are stated in the Supplementary Tables referenced in the respective figure legend. All bar and line graphs display mean ± s.e.m. or data from individual mice, as indicated.

### Reporting summary

Further information on research design is available in the Nature Research Reporting Summary linked to this article.

## Supplementary information

Supplementary Information

Supplementary Data 1

Supplementary Data 2

Description of Supplementary Files

Reporting Summary

## Data Availability

All raw data for behavioural experiments can be obtained from the corresponding author upon reasonable request. All source data for the main figures are contained in Supplementary Data 1. Gene-expression data are available from the Allen Institute for Brain Sciences (https://portal.brain-map.org/) and through CytosploreViewer (https://viewer.cytosplore.org); the source data for the differential gene expression analysis of GPCRs are available as Supplementary Data 2.

## Code availability

Scripts of all task files applied in custom-made operant boxes^64^ can be obtained from https://github.com/KaetzelLab/Operant-Box-Code and design files for such operant boxes are deposited at https://github.com/KaetzelLab/Operant-Box-Design-Files. IgorPro code to extract differentially expressed GPCR genes from results files provided by CytosploreViewer is supplied as Supplementary Code.

## References

[1] Dalley JW, Robbins TW (2017). Fractionating impulsivity: neuropsychiatric implications. Nat. Rev. Neurosci..

[2] McHugh C, Balaratnasingam S (2018). Impulsivity in personality disorders: current views and future directions. Curr. Opin. Psychiatry.

[3] Bari A, Robbins TW (2013). Inhibition and impulsivity: behavioral and neural basis of response control. Prog. Neurobiol..

[4] Chamorro J (2012). Impulsivity in the general population: a national study. J. Psychiatr. Res..

[5] Dalley JW, Everitt BJ, Robbins TW (2011). Impulsivity, compulsivity, and top-down cognitive control. Neuron.

[6] Jupp B, Dalley JW (2014). Convergent pharmacological mechanisms in impulsivity and addiction: insights from rodent models. Br. J. Pharmacol..

[7] Moeller FG, Barratt ES, Dougherty DM, Schmitz JM, Swann AC (2001). Psychiatric aspects of impulsivity. Am. J. Psychiatry.

[8] Moeller FG (2001). The impact of impulsivity on cocaine use and retention in treatment. J. Subst. Abus. Treat..

[9] Pattij T, De Vries TJ (2013). The role of impulsivity in relapse vulnerability. Curr. Opin. Neurobiol..

[10] Bari A, Dalley JW, Robbins TW (2008). The application of the 5-choice serial reaction time task for the assessment of visual attentional processes and impulse control in rats. Nat. Protoc..

[11] Lee H-M, Giguere PM, Roth BL (2014). DREADDs: novel tools for drug discovery and development. Drug Discov. Today.

[12] Hodge RD (2019). Conserved cell types with divergent features in human versus mouse cortex. Nature.

[13] Tasic B (2018). Shared and distinct transcriptomic cell types across neocortical areas. Nature.

[14] Saunders A (2018). Molecular diversity and specializations among the cells of the adult mouse brain. Cell.

[15] Logue SF, Gould TJ (2014). The neural and genetic basis of executive function: attention, cognitive flexibility, and response inhibition. Pharmacol. Biochem. Behav..

[16] Overington JP, Al-Lazikani B, Hopkins AL (2006). How many drug targets are there?. Nat. Rev. Drug Discov..

[17] Hauser AS, Attwood MM, Rask-Andersen M, Schiöth HB, Gloriam DE (2017). Trends in GPCR drug discovery: new agents, targets and indications. Nat. Rev. Drug Discov..

[18] Sriram K, Insel PA (2018). G protein-coupled receptors as targets for approved drugs: how many targets and how many drugs?. Mol. Pharmacol..

[19] Armbruster BN, Li X, Pausch MH, Herlitze S, Roth BL (2007). Evolving the lock to fit the key to create a family of G protein-coupled receptors potently activated by an inert ligand. Proc. Natl Acad. Sci..

[20] Jendryka M (2019). Pharmacokinetic and pharmacodynamic actions of clozapine-N-oxide, clozapine, and compound 21 in DREADD-based chemogenetics in mice. Sci. Rep..

[21] Dembrow N, Johnston D (2014). Subcircuit-specific neuromodulation in the prefrontal cortex. Front. Neural Circuits.

[22] Higgins GA, Ballard TM, Huwyler J, Kemp JA, Gill R (2003). Evaluation of the NR2B-selective NMDA receptor antagonist Ro 63-1908 on rodent behaviour: evidence for an involvement of NR2B NMDA receptors in response inhibition. Neuropharmacology.

[23] Higgins GA (2016). Enhanced attention and impulsive action following NMDA receptor GluN2B-selective antagonist pretreatment. Behav. Brain Res..

[24] Davis-Reyes BD (2019). Profile of cortical N-methyl-D-aspartate receptor subunit expression associates with inherent motor impulsivity in rats. Biochem. Pharmacol..

[25] Niswender CM, Conn PJ (2010). Metabotropic glutamate receptors: physiology, pharmacology, and disease. Annu. Rev. Pharmacol. Toxicol..

[26] Arnsten AFT (2009). Toward a new understanding of attention-deficit hyperactivity disorder pathophysiology. CNS Drugs.

[27] Galici R, Echemendia NG, Rodriguez AL, Conn PJ (2005). A selective allosteric potentiator of metabotropic glutamate (mGlu) 2 receptors has effects similar to an orthosteric mGlu2/3 receptor agonist in mouse models predictive of antipsychotic activity. J. Pharmacol. Exp. Ther..

[28] Hiyoshi T (2014). Neurophysiologic and antipsychotic profiles of TASP0433864, a novel positive allosteric modulator of metabotropic glutamate 2 receptor. J. Pharmacol. Exp. Ther..

[29] Kätzel, D., Nicholson, E., Schorge, S., Walker, M. C. & Kullmann, D. M. Chemical–genetic attenuation of focal neocortical seizures. *Nat. Commun*. **5**, 3847 (2014). 10.1038/ncomms4847 PMC4050272 24866701

[30] Pillidge K, Porter AJ, Vasili T, Heal DJ, Stanford SC (2014). Atomoxetine reduces hyperactive/impulsive behaviours in neurokinin-1 receptor ‘knockout’ mice. Pharmacol. Biochem. Behav..

[31] Pillidge K (2014). The behavioural response of mice lacking NK1 receptors to guanfacine resembles its clinical profile in treatment of ADHD. Br. J. Pharmacol..

[32] Porter AJ (2015). A lack of functional NK1 receptors explains most, but not all, abnormal behaviours of NK1R-/- mice1. Genes Brain Behav..

[33] Pillidge, K., Porter, A. J., Young, J. W. & Stanford, S. C. Perseveration by NK1R-/- (‘knockout’) mice is blunted by doses of methylphenidate that affect neither other aspects of their cognitive performance nor the behaviour of wild-type mice in the 5-Choice Continuous Performance Test. *J. Psychopharmacol.* (2016) 10.1177/0269881116642541. 10.1177/0269881116642541 PMC4994704 27097734

[34] Sharp SI (2014). Genetic association of the tachykinin receptor 1 TACR1 gene in bipolar disorder, attention deficit hyperactivity disorder, and the alcohol dependence syndrome.. Am. J. Med. Genet. B Neuropsychiatr. Genet..

[35] Yan TC (2010). NK1 (TACR1) receptor gene ‘knockout’ mouse phenotype predicts genetic association with ADHD. J. Psychopharmacol..

[36] Weir RK (2014). The influence of test experience and NK1 receptor antagonists on the performance of NK1R-/- and wild type mice in the 5-Choice Serial Reaction-Time Task. J. Psychopharmacol..

[37] Yan TC (2011). Performance deficits of NK1 receptor knockout mice in the 5-Choice Serial Reaction-Time Task: effects of d-amphetamine, stress and time of day. PLoS One.

[38] Tsutsui-Kimura I (2016). Neuronal codes for the inhibitory control of impulsive actions in the rat infralimbic cortex. Behav. Brain Res..

[39] Chudasama Y (2003). Dissociable aspects of performance on the 5-choice serial reaction time task following lesions of the dorsal anterior cingulate, infralimbic and orbitofrontal cortex in the rat: differential effects on selectivity, impulsivity and compulsivity. Behav. Brain Res..

[40] Benn A, Robinson ESJ (2014). Investigating glutamatergic mechanism in attention and impulse control using rats in a modified 5-Choice Serial Reaction Time Task. PLoS One.

[41] Luchicchi, A. et al. Sustained attentional states require distinct temporal involvement of the dorsal and ventral medial prefrontal cortex. *Front. Neural Circuits* **10**, (2016). 10.3389/fncir.2016.00070 PMC5005373 27630545

[42] Koike H (2016). Chemogenetic inactivation of dorsal anterior cingulate cortex neurons disrupts attentional behavior in mouse. Neuropsychopharmacology.

[43] Chudasama Y, Muir JL (2001). Visual attention in the rat: a role for the prelimbic cortex and thalamic nuclei?. Behav. Neurosci..

[44] Hvoslef-Eide, M. et al. Effects of anterior cingulate cortex lesions on a continuous performance task for mice. *Brain Neurosci. Adv*. **2**, 1–12 (2018). 10.1177/2398212818772962 PMC6546594 31168482

[45] Jupp B (2013). Dopaminergic and GABA-ergic markers of impulsivity in rats: evidence for anatomical localisation in ventral striatum and prefrontal cortex. Eur. J. Neurosci..

[46] Pliszka SR (2006). Neuroimaging of inhibitory control areas in children with attention deficit hyperactivity disorder who were treatment naive or in long-term treatment. Am. J. Psychiatry.

[47] Golchert J (2017). In need of constraint: understanding the role of the cingulate cortex in the impulsive mind. NeuroImage.

[48] Silveri MM (2013). Frontal lobe γ-aminobutyric acid levels during adolescence: associations with impulsivity and response inhibition. Biol. Psychiatry.

[49] Crawford JT, Roberts DCS, Beveridge TJR (2013). The group II metabotropic glutamate receptor agonist, LY379268, decreases methamphetamine self-administration in rats. Drug Alcohol Depend..

[50] Bäckström P, Hyytiä P (2005). Suppression of alcohol self-administration and cue-induced reinstatement of alcohol seeking by the mGlu2/3 receptor agonist LY379268 and the mGlu8 receptor agonist (S)-3,4-DCPG. Eur. J. Pharmacol..

[51] Pozzi L (2011). Attention deficit induced by blockade of N-methyl d-aspartate receptors in the prefrontal cortex is associated with enhanced glutamate release and cAMP response element binding protein phosphorylation: role of metabotropic glutamate receptors 2/3. Neuroscience.

[52] Wischhof L, Koch M (2012). Pre-treatment with the mGlu2/3 receptor agonist LY379268 attenuates DOI-induced impulsive responding and regional c-Fos protein expression. Psychopharmacology.

[53] Wischhof L, Hollensteiner KJ, Koch M (2011). Impulsive behaviour in rats induced by intracortical DOI infusions is antagonized by co-administration of an mGlu2/3 receptor agonist. Behav. Pharmacol..

[54] Imre G (2007). The preclinical properties of a novel group II metabotropic glutamate receptor agonist LY379268. CNS Drug Rev..

[55] Nikiforuk A (2010). Effects of a positive allosteric modulator of Group II metabotropic glutamate receptors, LY487379, on cognitive flexibility and impulsive-like responding in rats. J. Pharmacol. Exp. Ther..

[56] Salih H (2015). Pharmacokinetic and pharmacodynamic characterisation of JNJ-40411813, a positive allosteric modulator of mGluR2, in two randomised, double-blind phase-I studies. J. Psychopharmacol..

[57] Litman RE (2016). AZD8529, a positive allosteric modulator at the mGluR2 receptor, does not improve symptoms in schizophrenia: a proof of principle study. Schizophr. Res..

[58] Mehta MA (2018). Group II metabotropic glutamate receptor agonist prodrugs LY2979165 and LY2140023 attenuate the functional imaging response to ketamine in healthy subjects. Psychopharmacology.

[59] Stauffer VL (2013). Pomaglumetad methionil: No significant difference as an adjunctive treatment for patients with prominent negative symptoms of schizophrenia compared to placebo. Schizophr. Res..

[60] Adams DH (2013). A long-term, phase 2, multicenter, randomized, open-label, comparative safety study of pomaglumetad methionil (LY2140023 monohydrate) versus atypical antipsychotic standard of care in patients with schizophrenia. BMC Psychiatry.

[61] Tsien JZ (1996). Subregion- and cell type-restricted gene knockout in mouse brain. Cell.

[62] Felipe CD (1998). Altered nociception, analgesia and aggression in mice lacking the receptor for substance P. Nature.

[63] Grimm CM (2018). Schizophrenia-related cognitive dysfunction in the Cyclin-D2 knockout mouse model of ventral hippocampal hyperactivity. Transl. Psychiatry.

[64] Akam, T. et al. pyControl: Open source, Python based, hardware and software for controlling behavioural neuroscience experiments. *bioRxiv* (2021) 10.1101/2021.02.22.432227. 10.7554/eLife.67846 PMC8769647 35043782

[65] Olsen SR, Bortone DS, Adesnik H, Scanziani M (2012). Gain control by layer six in cortical circuits of vision. Nature.

[66] Bortone DS, Olsen SR, Scanziani M (2014). Translaminar inhibitory cells recruited by layer 6 corticothalamic. Neurons Suppress Vis. Cortex. Neuron.

