# Supplementary material for: Control of impulsivity by Gi-protein signalling in layer-5 pyramidal neurons of the anterior cingulate cortex
Source: Commun Biol. 2021 Jun 2;4:662. doi: 10.1038/s42003-021-02188-w (PMC8172539; doi:10.1038/s42003-021-02188-w)
Supplement: Supplementary file 1 — Supplementary Information [file 42003_2021_2188_MOESM1_ESM.docx]

Supplementary Information

# Control of impulsivity by G_i_-protein signalling in layer-5 pyramidal neurons of the anterior cingulate cortex

Bastiaan van der Veen^1^, Sampath K.T. Kapanaiah^1^, Kasyoka Kilonzo^1^, Peter Steele-Perkins^1^, Martin M. Jendryka^1^, Stefanie Schulz^1^, Bosiljka Tasic^3^, Zizhen Yao^3^, Hongkui Zeng^3^, Thomas Akam^4^, Janet R. Nicholson^2^, Birgit Liss^1,5^, Wiebke Nissen^2^, Anton Pekcec^2^, Dennis Kätzel ^1,^*

^1^Institute of Applied Physiology, Ulm University, Ulm, Germany

^2^Boehringer Ingelheim Pharma GmbH & Co. KG, Div. Research Germany, Biberach an der Riss, Germany

^3^Allen Institute for Brain Science, Seattle, WA, USA

^4^Department of Experimental Psychology, University of Oxford, Oxford, UK

^5^Linacre College and New College, University of Oxford, Oxford, UK

* Correspondence: dennis.kaetzel@uni-ulm.de; +49 731 500 33770; Fax +49 731 500 33779; Institute of Applied Physiology, Ulm University, Albert-Einstein-Allee 11, 89081 Ulm, Germany

## Supplementary Figures


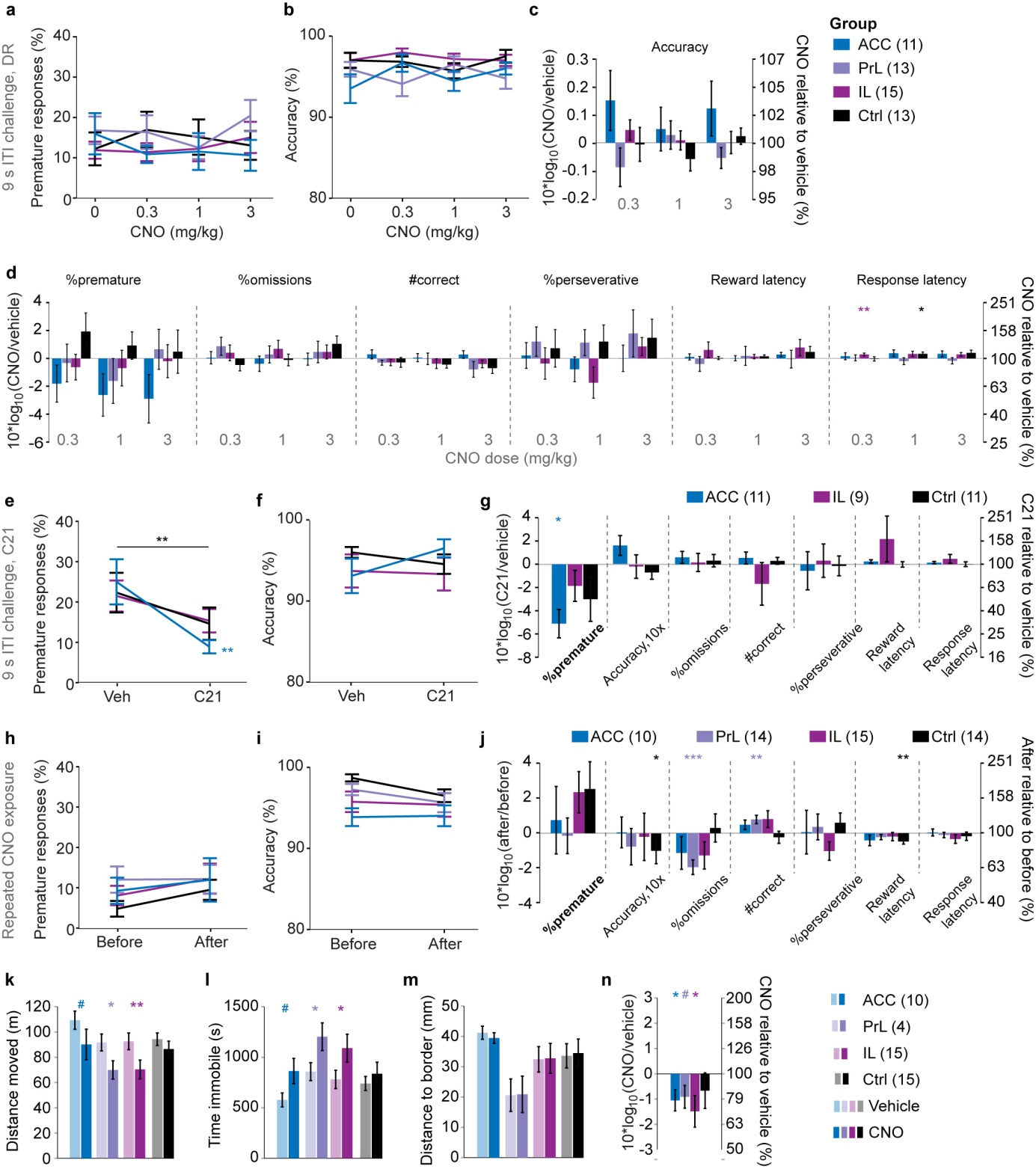


**Supplementary Figure 1 | hM4Di-mediated modulation of excitatory cells in prefrontal subdivisions.** (**a**, **b**) Premature responding (a) and accuracy (b) during the dose-response curve in the 4 subgroups (see colour legend in (c)) for the CamKIIα::hM4Di mice in the 5-CSRTT impulsivity challenge. (**c**, **d**) Data from the same challenge as (a,b) but accuracy (c) and other 5-CSRTT parameters (d) expressed as log_10_-transform of the within-subject ratio (value after CNO / value after vehicle); asterisks indicate 1-sample *t*-test against 0. (**e-g**) Premature responding (e), accuracy (f) and normalized parameters (g, as in c,d; accuracy is multiplied by 10 as this parameter shows comparatively small variations) for the test of 3.3 mg/kg compound 21 (C21) in the impulsivity challenge. Black asterisks indicate significant effect of dose (e, RM-ANOVA), coloured asterisks indicate within-group effects (f, paired post-hoc test; g, one-sample *t*-test against 0) for the group identified by the colour. (**h-j**) same analysis as the corresponding panels above (e-g) but for behaviour in the 9 s fITI challenge after saline injection before and after 3 intermediate days during which the baseline protocol was performed daily after 10 mg/kg CNO injection. (**k-n**) Parameters from locomotor-activity testing (see y-axes) after vehicle and 10 mg/kg CNO injection for the 4 subgroups (as indicated by colour) for absolute values (k-m) and log_10_-transform of the within-subject ratio (value after CNO / value after vehicle) for distance moved (n). See Supplementary Tables 3 and 4 for statistics and reasons for varying *N*-numbers across experiments, respectively. *N-*numbers for (a-d) stated in (c), for (e-g) in (g), for (h-j) in (j), and for (k-n) in (n). * *P <* 0.05; ** *P <* 0.01; *** *P* ≤ 0.001; error bars, s.e.m.


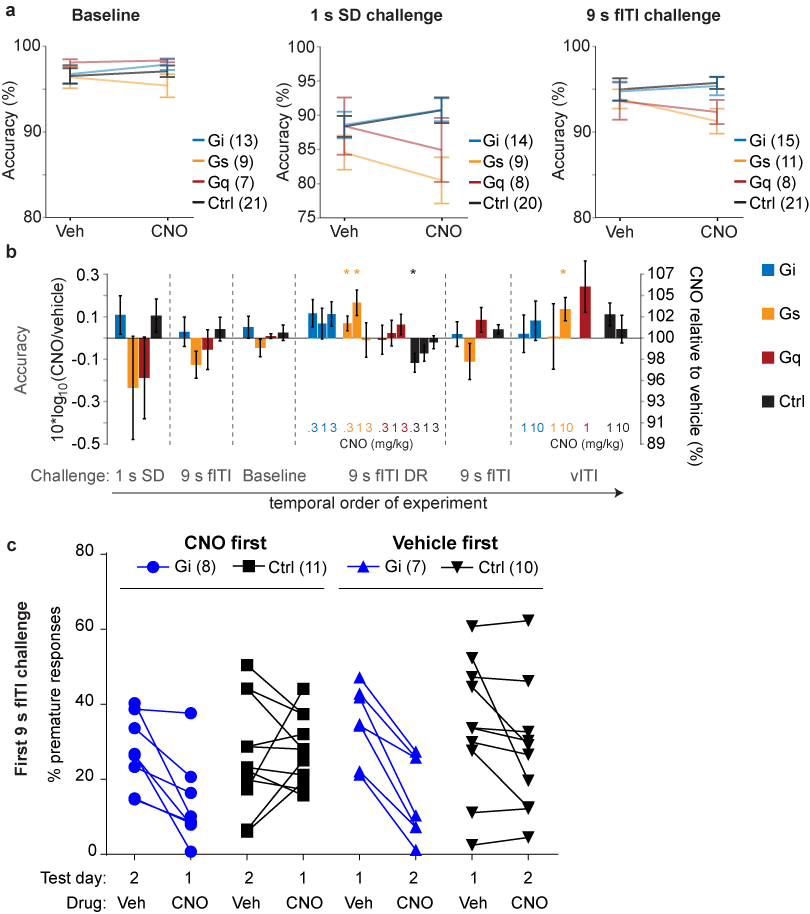


**Supplementary Figure 2 | Repeated 5-CSRTT behavioural challenges during manipulation of ACC L5-PCs.** (**a**) Attentional accuracy after injection of vehicle (Veh) or CNO for the four subgroups of the cohort [G_i_ (blue), G_s_ (orange), G_q_ (red), mCherry-controls (Ctrl; grey/black), N-numbers in brackets] and 5-CSRTT challenge condition indicated above. (**b**) CNO-induced changes of attentional accuracy expressed as log_10_-transform of the within-subject ratio (value after CNO / value after vehicle) as shown for premature responses in Fig. 2g for the different parametric challenges in order of their execution, including dose-response (DR) experiments in the 9 s-fITI- and vITI-challenges as indicated. Asterisks in (a) and (b) indicate 1-sample *t*-test against 0. *N*-numbers for the various challenges along with their detailed statistical analysis can be found in Supplementary Tables 7-9 and reasons for varying *N*-numbers in Supplementary Table 3. * *P <* 0.05; error bars, s.e.m. (**c**) Premature responding during the first 9s-fITI challenge shown for each mouse in the Gi and control groups split by the order of within-subject application of CNO and vehicle. Gi-mice that received *CNO* first have the same impulsivity-level under vehicle as those Gi-mice that received *vehicle* first and as control mice of either subgroup, demonstrating that acute CNO/Gi-modulation does not alter later behaviour in the same challenge in the longer term. *N*-numbers stated in colour legend.


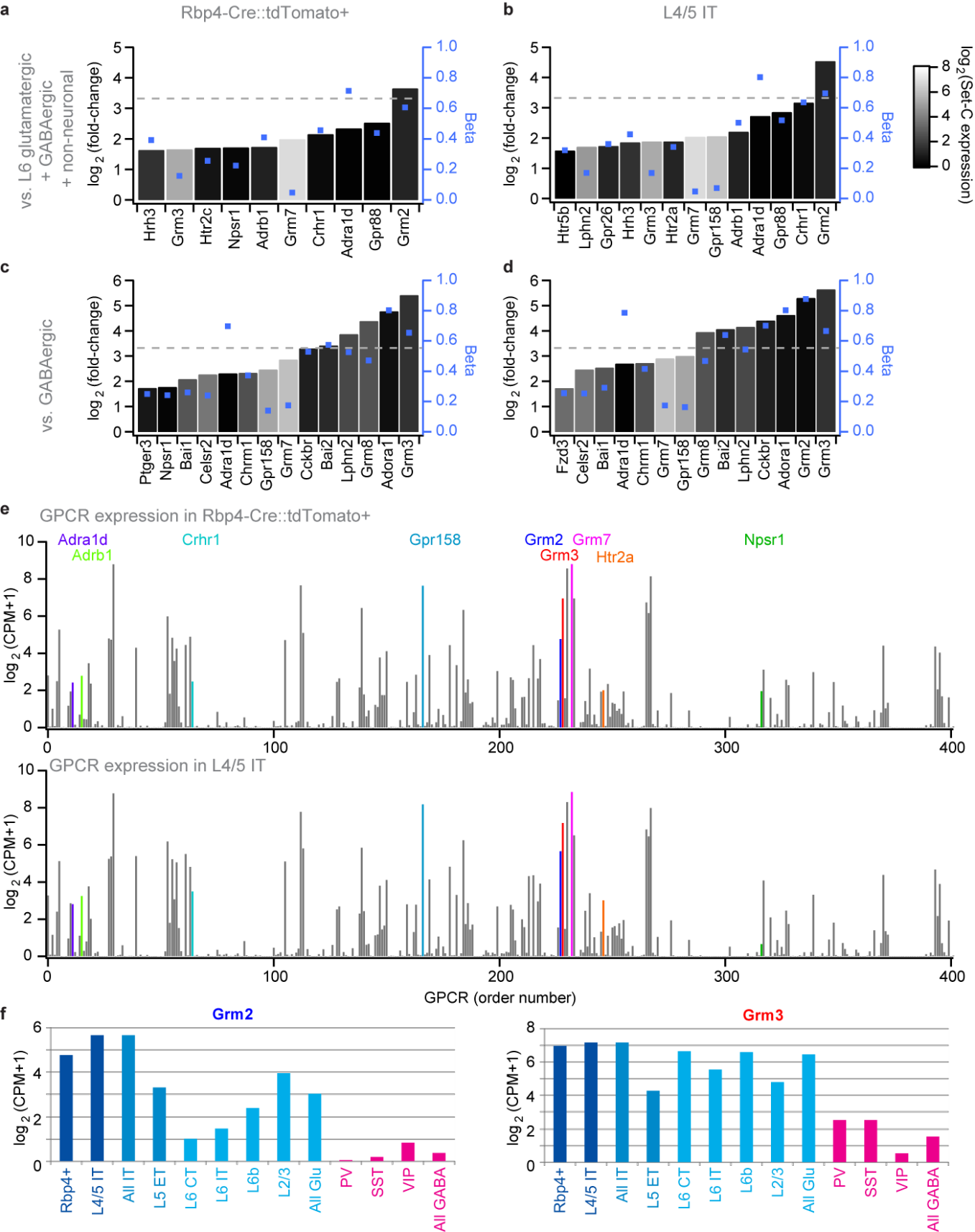


**Supplementary Figure 3 | Differential gene expression analysis of mouse ACC L5-PCs against other cortical cell-types.** (**a-d**) Display as in Fig. 5 just for smaller non-target sets of cells. Differential gene expression values (log_2_-transformed ratios of gene expression in target set of ACC L5-cells relative to the non-target sets stated on the left (grey label), difference of share of cells *with* expression in target set and non-target set (Beta, blue dots, right axis), and average expression level in non-target set (log_2_(CPM+1); grey scale) are displayed for all GPCR-encoding genes that are among differentially expressed transcripts in target set (corrected *P <* 0.05) and are expressed at least 3fold higher in the target set compared to the non-target set. Horizontal dotted line indicates a 10fold higher expression in the target set (referring to left axis). The calculation has been performed by either selecting tdTomato-positive excitatory cells extracted from an Rbp4-Cre::Ai14 line (i.e. using the same selection as for the chemogenetic experiments in Figs 3 and 4, a, c), or by selecting clusters of L4/5 intertelencephalic-projecting (IT) cells according to the metadata (b, d). (**e**) Absolute gene expression across all 402 analysed non-sensory GPCRs coded by their alphabetical order number as listed in Supplementary Table 18 in the two target sets (Set-T, as named) used for gene-expression analysis. Genes that are significantly differentially expressed in the most conservative comparison (Fig. 5a,b) are colour-coded and named. Note, that such differentially expressed genes do not necessarily show the highest expression levels in the target cells. (**f**) Absolute average expression levels in the indicated cell types and clusters, including target cell clusters of varying specificity (dark blue), other excitatory cells (light blue), and GABAergic interneurons (magenta). See Supplementary Table 18 for details on used cells, investigated GPCR-genes, and results.

**
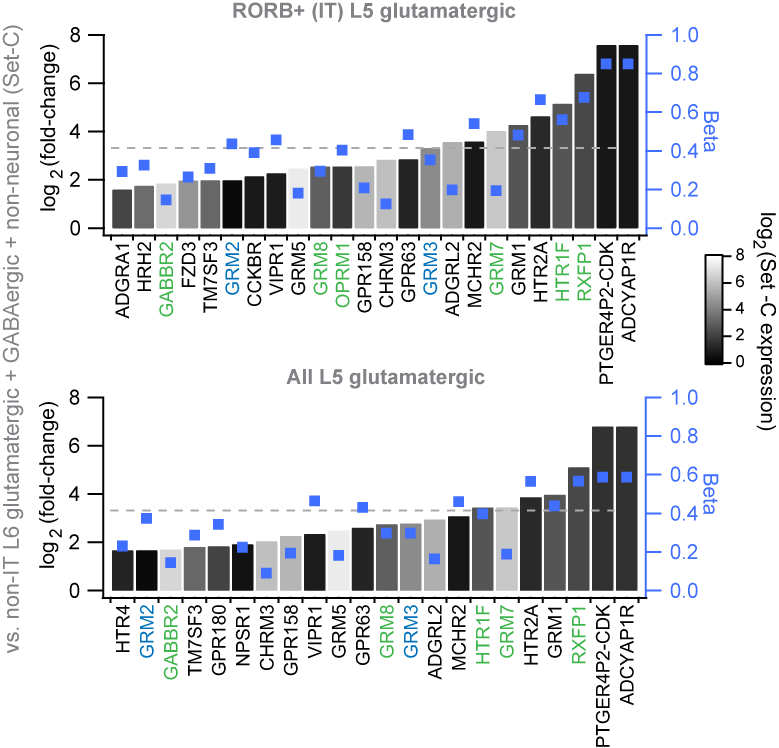
**

**Supplementary Figure 4 | Differential gene expression analysis of human cingulate L5-PCs against other cortical cell-types.** Display as in Supplementary Fig. 3a, but for an analysis of single-cell transcriptomes collected from the human cingulate cortex (including not only the anterior but also the posterior cingulate cortex). Layer 5 cells have been selected either narrowly based on expression of the IT-marker RORB (clusters IT_L3-5 RORB, IT_L4-5 RORB, IT_L5_RORB; 868 cells; top panel) or more broadly including further clusters with L5 cells (clusters IT_L3-5; IT_L3-5_THEMIS, IT_L5-6_RORB, IT_L5-6_THEMIS, ET_L5, IT_L5_6 CAR3, NP_L5_6, in addition to the narrow set; 1426 cells; bottom panel). GABAergic interneurons, non-neuronal cells and non-IT L6 / L6b pyramidal neurons were used as contrast set (Set-C) in which expression of the respective target gene should be low (clusters CT_L6, L6b, LAMP5, PAX, PVALB, SST, VIP, non-neuronal; 3254 cells). See Supplementary Table 18 for details on used cells, investigated GPCR-genes, and results. Differential gene expression values (log_2_-transformed ratios of gene expression in target set of ACC L5-cells relative to the non-target set stated on the left; grey label), difference of share of cells *with* expression in target set and non-target set (Beta, blue dots, right axis), and average expression level in non-target set (log_2_(CPM+1); Set-C; grey scale) are displayed for all human GPCR-encoding genes, out of 399, that are expressed at least 3fold higher in the target set compared to the non-target set. No *P*-value correction was applied here, given that the goal of this analysis was the confirmation of the presence of individual targets in the putative cells of interest. Horizontal dotted line indicates a 10fold higher expression in the target set (referring to left axis). Note that GRM2 and GMR3 (blue) are still expressed >3fold higher in the target set compared to the non-target set, but other GPCRs, not found with the analysis in mouse ACC, are also revealed. All identified GPCRs with known G_i_-coupling are highlighted green; note that the GPCR encoded by RXFP1 also couples to G_s_^1,2^, which is why rather 5-HTR1F^3^ can be regarded as the most selectively expressed G_i_-coupled receptor^4–6^ according to this analysis. However, some of the differences between human and mouse differential gene expression results may also be due to the necessarily imperfect match of cell clusters selected within each species; especially the presence of some L3 and L6 pyramidal cells in the human - but not the mouse – target set due to the absence of sole L4/5 clusters in the human dataset. A further source of differences may be the incorporation of the whole cingulum in the human dataset.


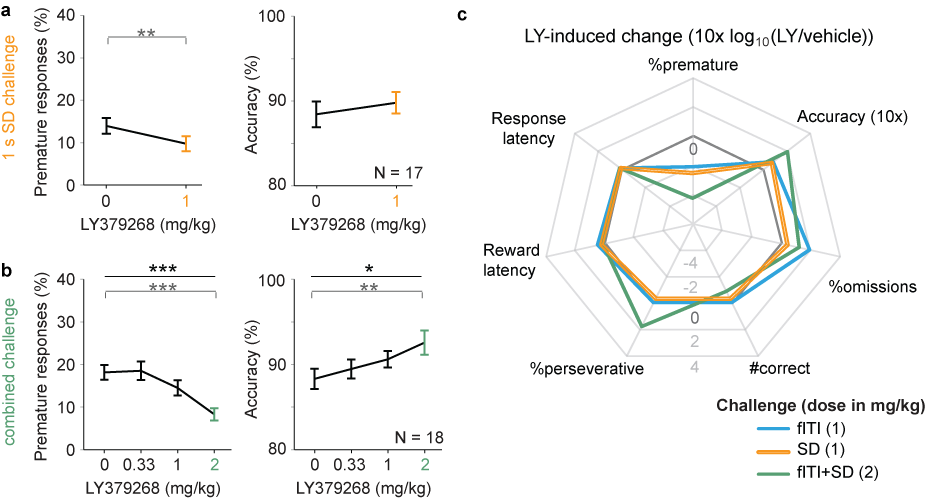


**Supplementary Figure 5 | Anti-impulsive action of LY379268 in SD and combined challenges.** (**a-b**) Absolute values of %premature responses (left) and attentional accuracy (right) after injection of vehicle (0) or various doses of LY379268 (LY), as indicated, in the challenge condition named on the left. The same group of 18 male C57BL/6 mice was used for every experiment with contributing *N*-numbers indicated in the accuracy panels. Black stars indicate significant effects of dose; grey stars indicate significant paired post-hoc comparisons between dose-levels (Sidak). (**c**) Response profiles of LY-induced changes relative to the vehicle/vehicle condition measured as average log_10_-transform of the within-subject ratio (value after drug / value after vehicle) for relevant behavioural performance parameters are shown for the challenges and dose levels indicated by the colour in the legends (e). Profiles for the lowest tested doses at which a significant reduction in premature responding was observed in each condition as displayed in (a, b and Fig. 6a) are shown. See Supplementary Table 7 for statistics and reasons for varying *N*-numbers across experiments, respectively. * *P <* 0.05; ** *P <* 0.01; *** *P* ≤ 0.001; error bars, s.e.m.

**
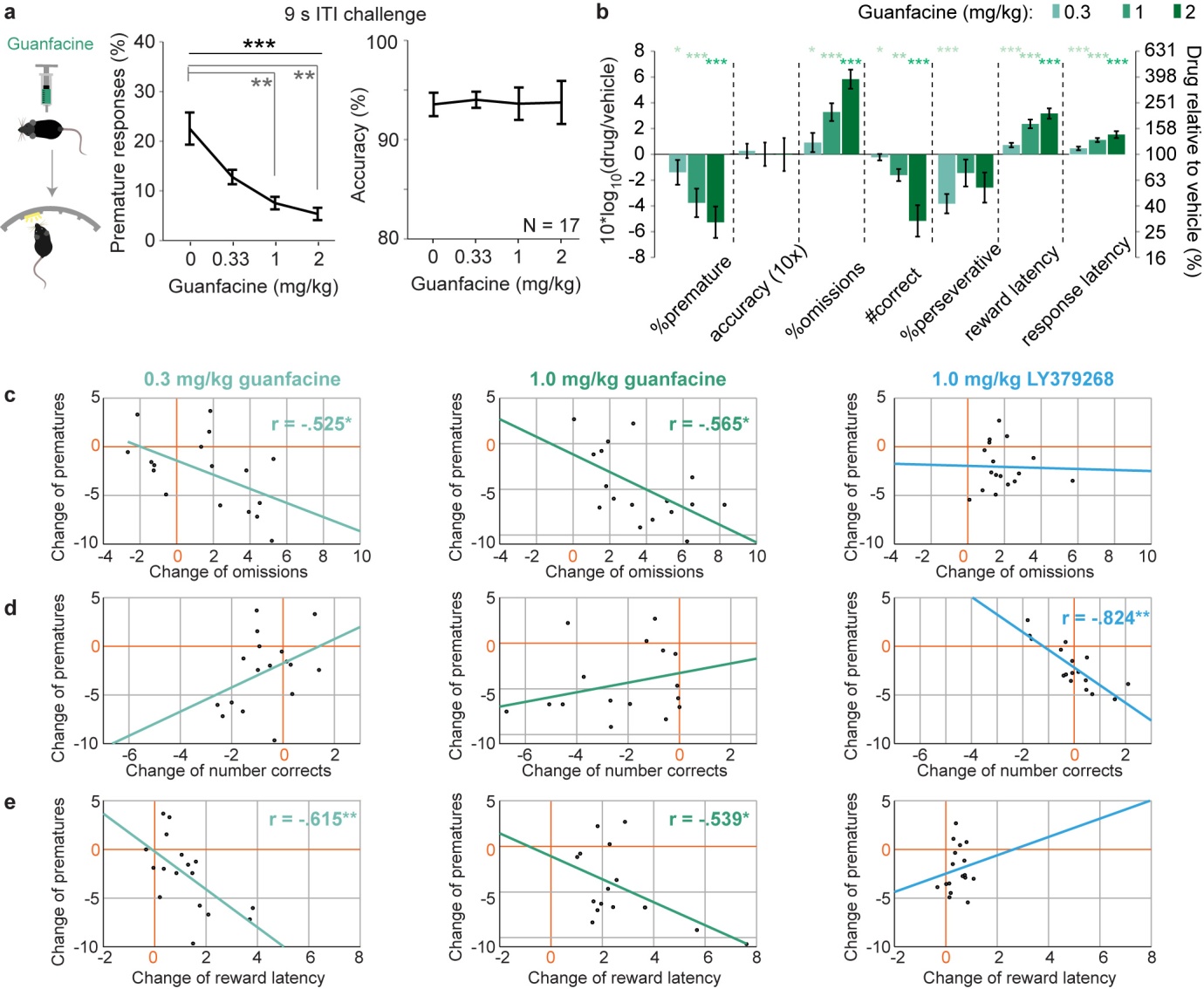
**

**Supplementary Figure 6 | Anti-impulsive and sedative action of guanfacine in the 9s-fITI challenge.** (**a**) Absolute values of %premature responses (left) and attentional accuracy (right) after injection of vehicle (0) or various doses of guanfacine, as indicated, in the 9s-fITI challenge condition. Contributing *N*-numbers indicated in the accuracy panels. Black stars indicate significant effect of dose; grey stars indicate significant paired post-hoc comparisons between dose-levels (Sidak). (**b**) Guanfacine-induced changes in the corresponding experiment shown in (a) measured as log_10_-transform of the within-subject ratio (value after drug(s) / value after vehicle) for relevant behavioural performance parameters on the 5-CSRTT are shown for the tested doses and drug combinations as indicated in the colour legend. Asterisks indicate one-sample *t*-tests against 0, colour-coded according to the dose. (**c-e**) Correlation scatter plots with linear fits plotting the drug-induced changes of %premature responding against the changes %omisisons (c), number of correct responses (d), and reward latency (e) for 0.3 mg/kg guanfacine (left), 1 mg/kg guanfacine (middle), and 1 mg/kg LY379268 (right). The change of a given parameter used for this correlation is measured as log_10_-transform of the within-subject ratio (value after drug / value after vehicle). Pearson correlation coefficients are stated in the respective panel where significant. Note that a decrease in premature responding is associated with an increase of correct responses under LY-treatment, but not under guanfacine treatment, while omissions and reward latencies increase with reduced premature responding induced by guanfacine, but not LY. *N* = 17. See Supplementary Tables 11 and 12 for statistics.

**
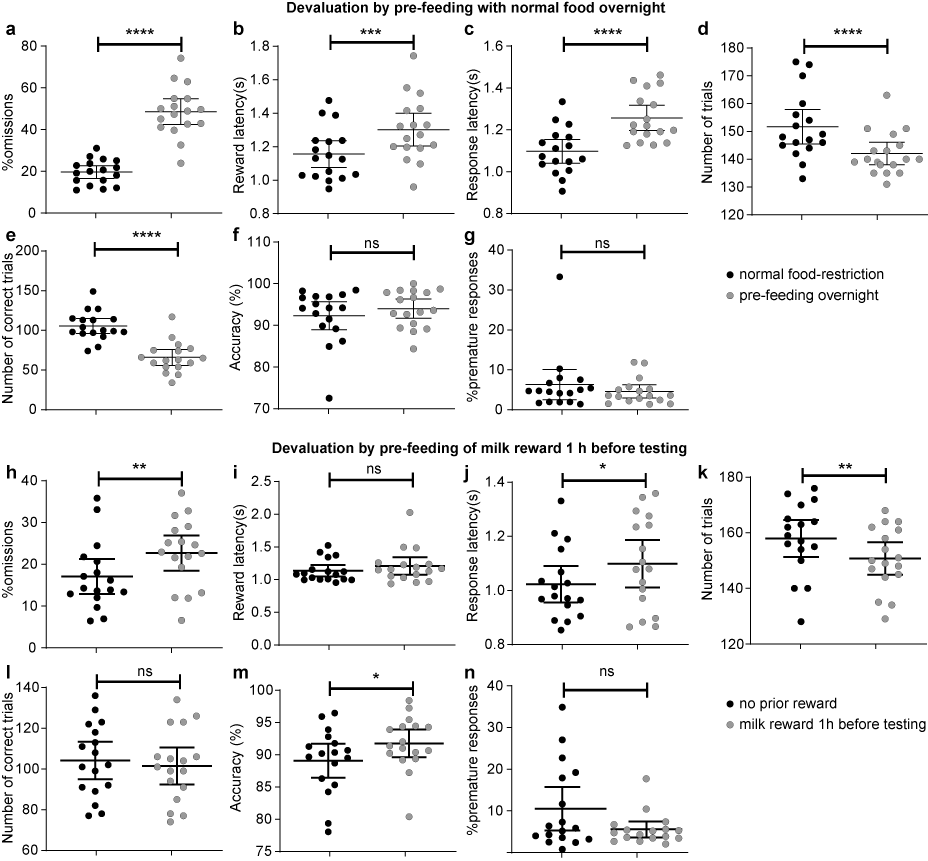
**

**Supplementary Figure 7 | Devaluation of milk reward.** (**a-g**) Data from a separate cohort of 17 male Rbp4-Cre mice which underwent devaluation tests on individual training days on the baseline stage (stage 5) in order to assess if the milk reward is hyper-palatable, possibly leading to strongly habitual task conductance or “addictive” reward seeking. Within-subject data is shown for the prior training day with normal food scheduling (black; ca. 2 g food per mouse overnight, depending on body weight) and training after pre-feeding with three times the amount of food they would normally receive (6 g in most cases; practically *ad libitum* feeding) overnight; no excess food was found in the cages in the morning, ca. 4 h before testing. Animals reached between 95-100% of their free-feeding weight as opposed to the usual 85-90% of free-feeding weight that they were kept under normal food scheduling. Task engagement (a), locomotor drive (b), processing speed (c), and the number of collected rewards (d) – and consequently the number of conducted trials (e) - all strongly decreased, while accuracy (f) and premature responding (g) remained unaffected. (**h-n**) Same display as in (a-g) and for the same cohort, but in this case for devaluation induced by prior feeding of the milk reward ca. 1 h before the start of the test (grey) relative to the prior training day without pre-feeding (black). 2 ml milk was given per mouse, which corresponds approximately to the mount that they would consume during a normal task session (100 correct responses); all mice consumed the reward within less than 10 min. Across parameters, the devaluation effect was less strong, however task engagement (h) and number of collected rewards (k) significantly decreased as well. Individual dots represent individual mice. Horizontal lines represent mean ± 95% confidence interval. *N* = 17. * *P <* 0.05; ** *P <* 0.01; *** *P* ≤ 0.001; **** *P* ≤ 0.0001

**
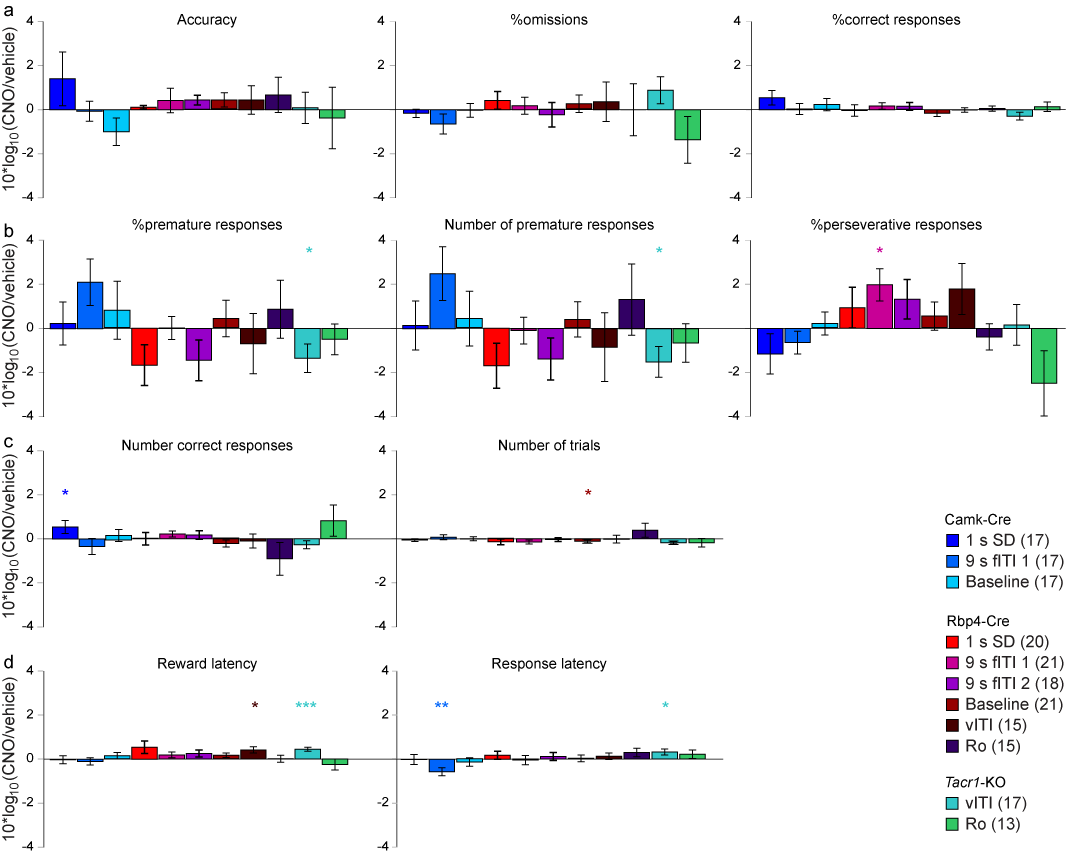
**

**Supplementary Figure 8 | Effect of CNO in control groups.** (**a-d**) Data from the control groups of the three cohorts used for chemogenetic experiments shown for all tests done with 10 mg/kg CNO (as shown in Fig. 1, 2, and 7) – cohort and challenge condition are identified by the colour legend. Data is expressed as log_10_-transform of the within-subject ratio (value after CNO / value after vehicle); asterisks indicate 1-sample *t*-test against 0. Shown are parameters (as named at the top of each panel) of sustained attention and task engagement (a), impulsivity or perseveration (b), absolute number of correct responses (obtained rewards) or trials (c), and latencies (d). No consistent effects of CNO are seen across cohorts and challenges in any parameter. *N*-numbers are stated in the colour legend. Graphs display mean±s.e.m. * *P <* 0.05; ** *P <* 0.01; *** *P* ≤ 0.001.


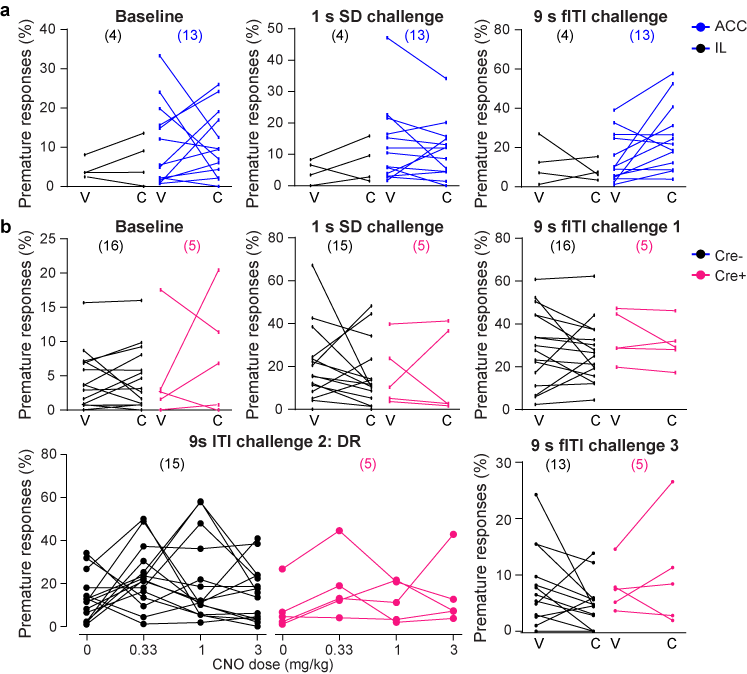


**Supplementary Figure 9 | Premature responding in subgroups of merged control groups.** (**a-b**) Premature responding in the primary challenges (named above each sub-panel; as shown in Fig. 1 and 2), under vehicle (V) and CNO (C) is shown for the two chemogenetic control groups that were merged from different controls: CamKIIα-Cre mice transfected with mCherry either in IL (black) or ACC (blue) (a), or mice from the Rbp4-Cre cohort that were either negative (black) or positive (magenta) for Cre (b). Note that Cre-negative mice have been transfected with mCherry as well, albeit driven from a viral CamKIIα-promoter (just like the controls shown in (a)), instead of through a Cre-dependent mechanism. Ro- and vITI challenges in Rbp4-Cre cohort not shown because only one Cre-positive mouse participated in those. Individual dot-lines represent individual mice. No effects of group or group-drug interactions were found in any condition (repeated-measures ANOVA within each protocol and across subgroups and drug doses). *N*-numbers are stated in respective sub-panels.

## Supplementary Tables


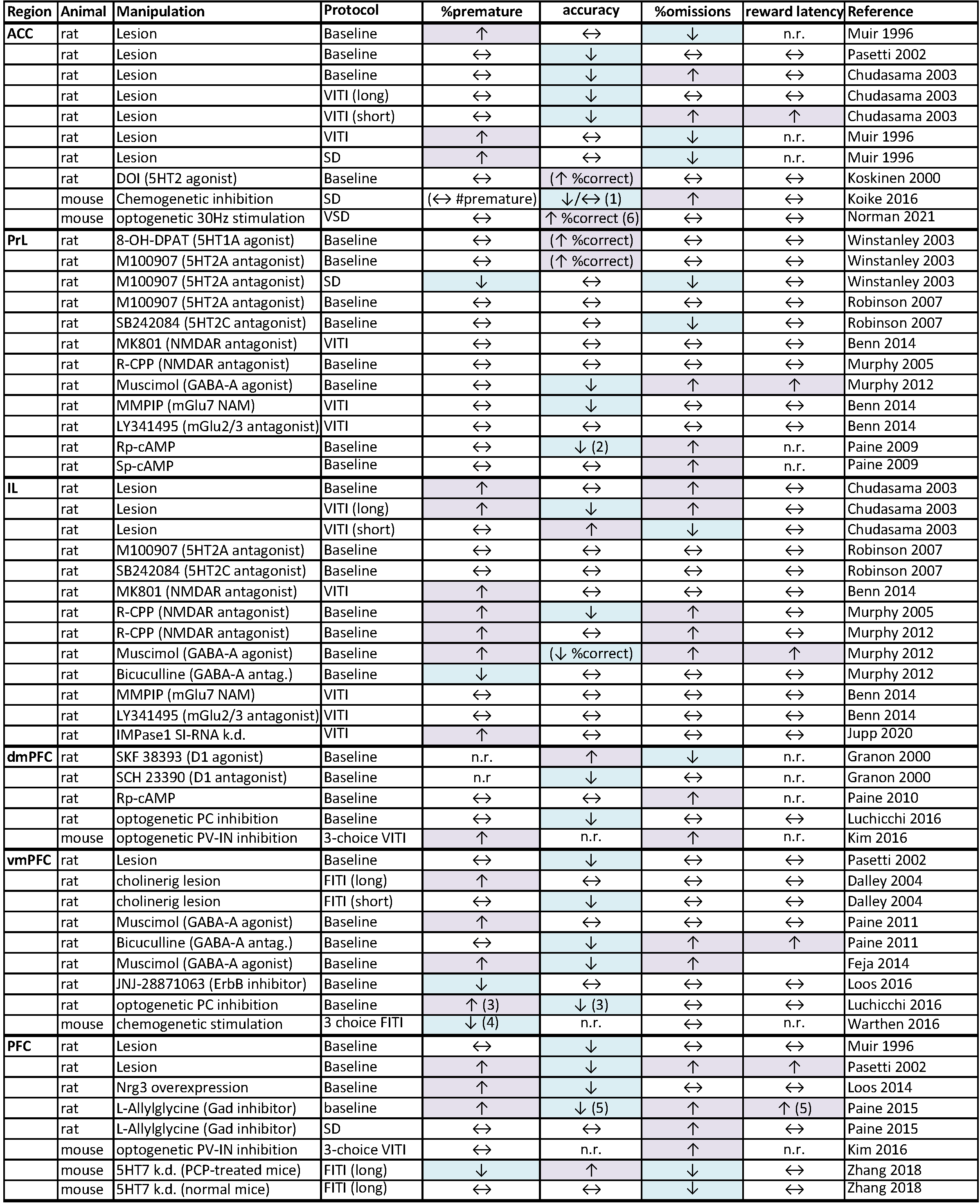


**Supplementary Table 1 | Studies with PFC manipulations in the rodent 5-CSRTT.** Studies with local lesions, inhibition, activation or drug infusions as indicated under “Manipulation” conducted in the indicated brain region during the 5-CSRTT or very similar tests like the 3-choice-serial-reaction-time task or the continuous performance test. Abbreviations: ACC, anterior cingulate cortex; IL, infralimbic cortex; PrL, prelimbic cortex; PFC, prefrontal cortex; dmPFC, dorso-medial PFC (usually Cg1 and dorsal PrL regions); vmPFC, ventromedial PFC (usually IL and ventral PrL); PC, pyramidal cell; PV-IN, parvalbumin interneuron; antag. antagonist; k.d., knock-down; NAM, negative allostric modulator; FITI, fixed-ITI (impulsivity) challenge; SD, stimulus-duration (attention) challenge; VITI variable ITI (impulsivity challenge); VSD, variable SD (attention challenge); ↑ increase of parameter; ↓ decrease of parameter; n.r., not reported; %correct, only %correct reported, not accuracy; #premature, only the number of premature responses reported, not the percent. References refer to (in alphabetical order): Benn 2014^7^, Chudasama 2003^8^, Granon 2000^9^, Jupp 2020^10^, Kim 2016^11^, Koike 2016^12^, Koskinen 2000^13^, Loos 2014^14^, Loos 2016^15^, Luchicchi 2016^16^, Muir 1996^17^, Murphy 2005^18^, Murphy 2012^18^, Norman 2021^19^, Paine 2009^20^, Paine 2011^21^, Paine 2015^22^, Pasetti 2002^23^, Robinson 2007^24^, Warthen 2016^25^, Winstanly 2003^26^, Zhang 2018^27^. Remarks: (1) Effect depending on SD; (2) Effect driven by top-performers; (3) Effects depending on timing of optogenetic inhibition; (4) Effect seen only in high-impulsive mice; (5) Effects seen only on the first day of testing; (6) Effect only seen with 30 Hz stimulation of ACC neurons that project to visual cortex after incorrect responses. %correct labels affects that were seen in the %correct parameter (number of correct responses divided by sum of omissions, correct and incorrect responses) as values for attentional accuracy values were not stated or no effect was seen in this measure.

|  | **Parameters of training** | | | **Criteria (2 consecutive days)** | | | |
| --- | --- | --- | --- | --- | --- | --- | --- |
| **Stage** | **SD (s)** | **LH (s)** | **ITI (s)** | **#correct** | **%correct** | **accuracy** | **%omissions** |
| 1 | 20 | 30 | 2 | >= 30 | >= 40 | - | - |
| 2 | 8 | 10 | 2 | >= 40 | >= 50 | - | - |
| 3 | 8 | 10 | 5 | >= 40 |  | >= 80 | <= 50 |
| 4 | 4 | 6 | 5 | >= 40 | - | >= 80 | <= 50 |
| 5/BL | 2 | 4 | 5 | >= 40 | - | >= 80 | <= 50 |
| **Challenges** | | | | | | | |
| 1 s SD | 1 | 3 | 5 | Attention challenge | | | |
| 9 s ITI | 2 | 4 | 9 | Impulsivity challenge | | | |
| Combined | 1 | 3 | 7 | Combined challenge | | | |
| vITI | 2 | 4 | 7, 9, 11, 13 | Variable ITI challenge | | | |

**Supplementary Table 2 | Training stages and challenges in the 5-CSRTT.** Stages 1-5 are the training stages that followed after the habituation training. The criteria had to be met on 2 consecutive days on each training stage in order to advance to the next stage. After the stated criteria were met on Stage 5, which also served as the baseline stage (BL) for intermittent training, chemogenetic or pharmacology experiments were started. Parameters used for the challenges are listed below. For the vITI challenge, trials with the 4 stated ITIs were mixed pseudo-randomly with equal probability of occurrence. BL, baseline protocol; SD, stimulus duration; LH, limited hold time that the animals were allowed to respond into the correct hole counted from the beginning of the SD; ITI, inter-trial interval; #correct, number of the correct responses; %correct, number of correct responses divided by the sum of trials with correct, incorrect or omitted responses.


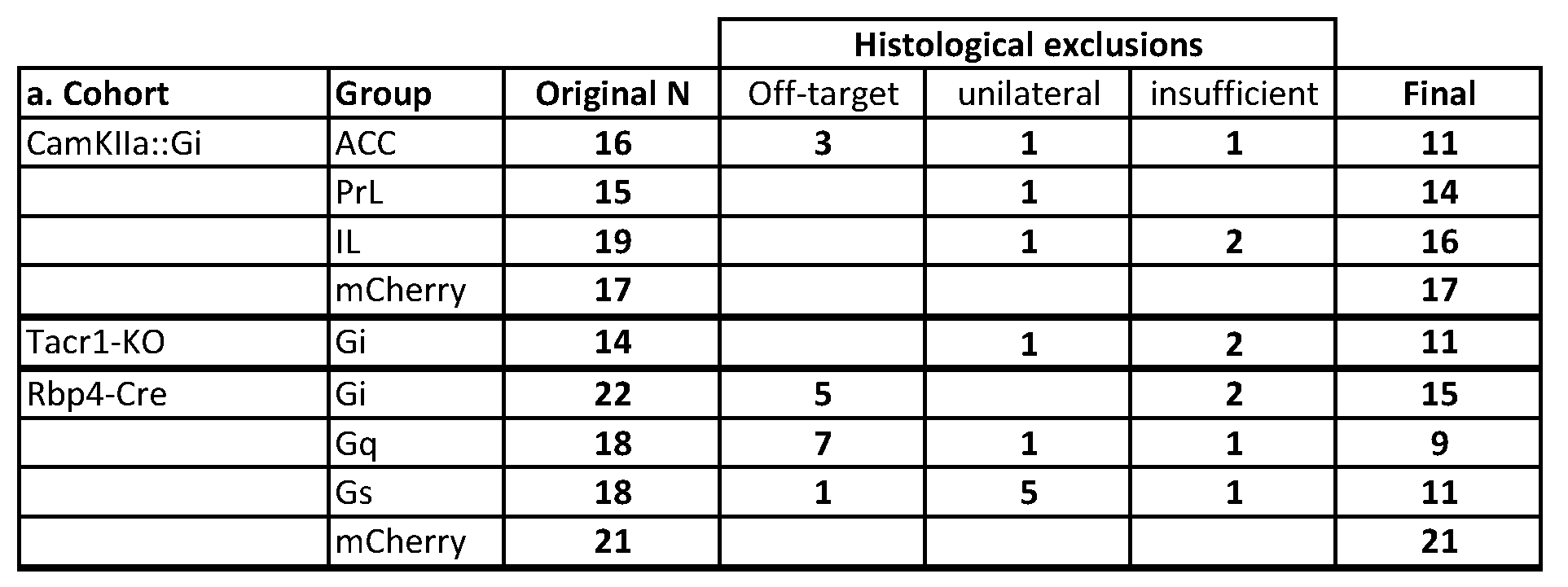

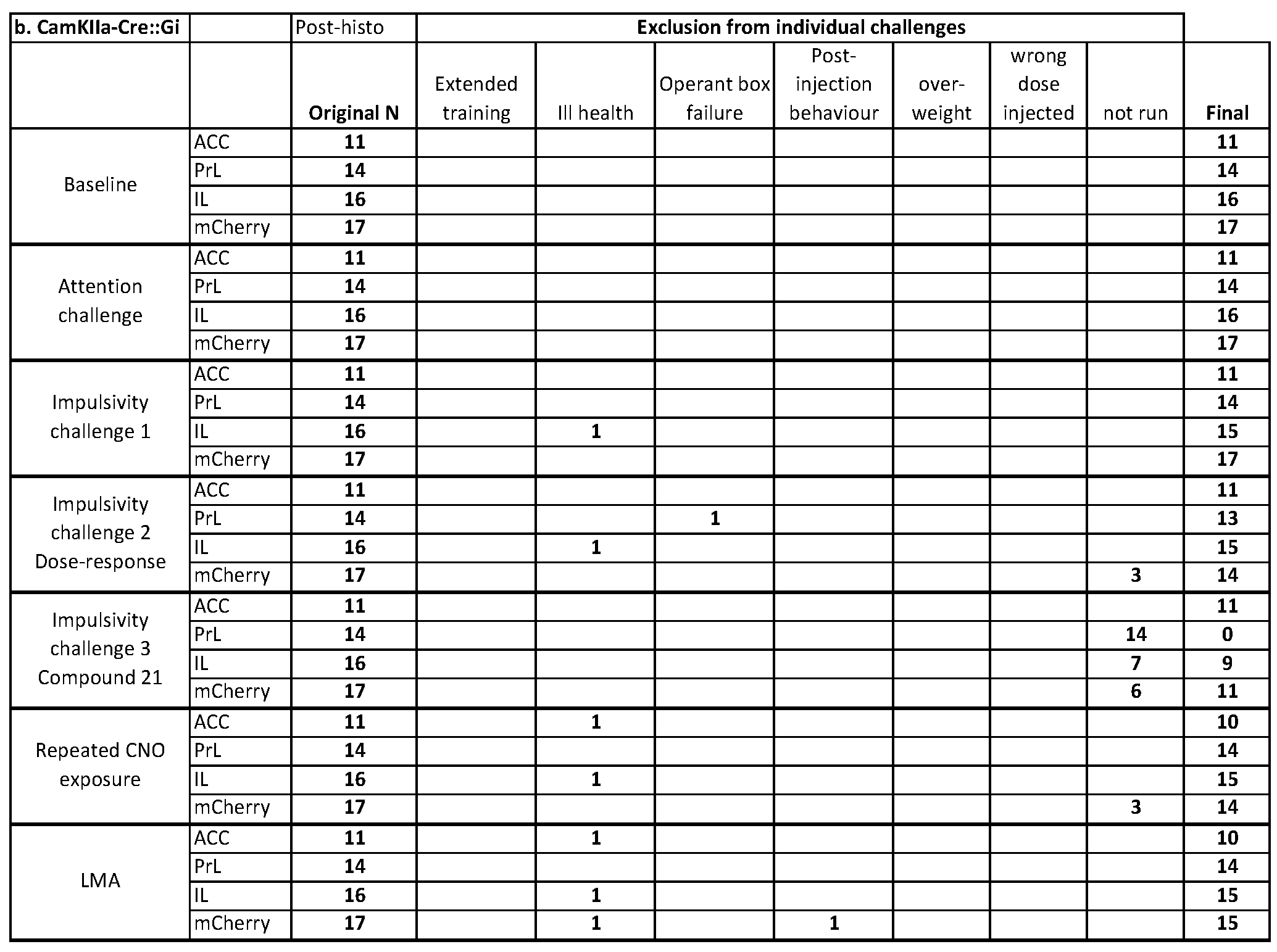

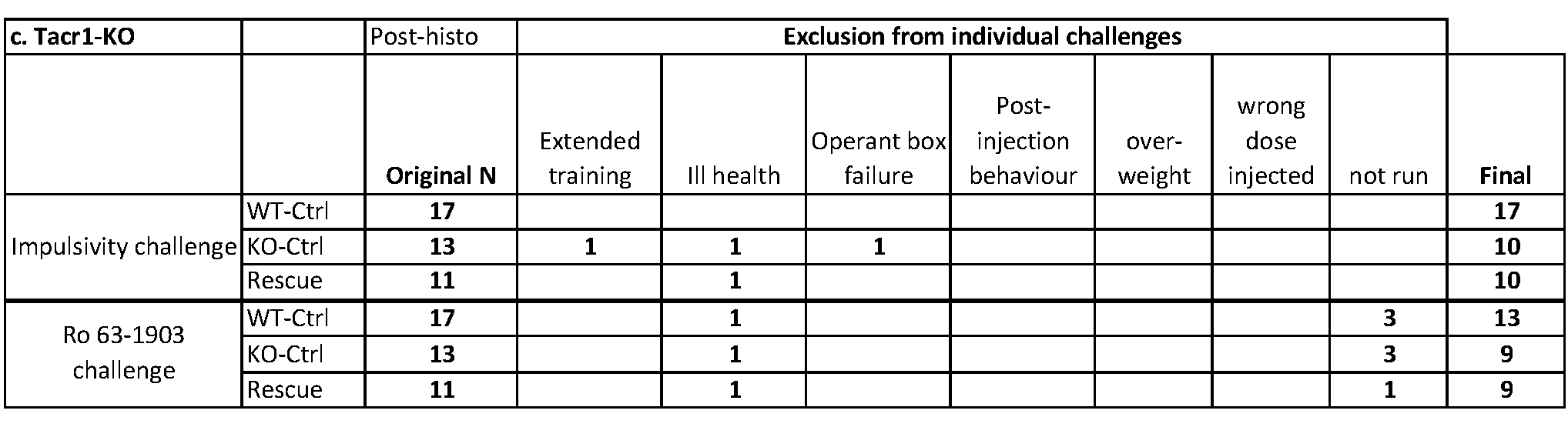


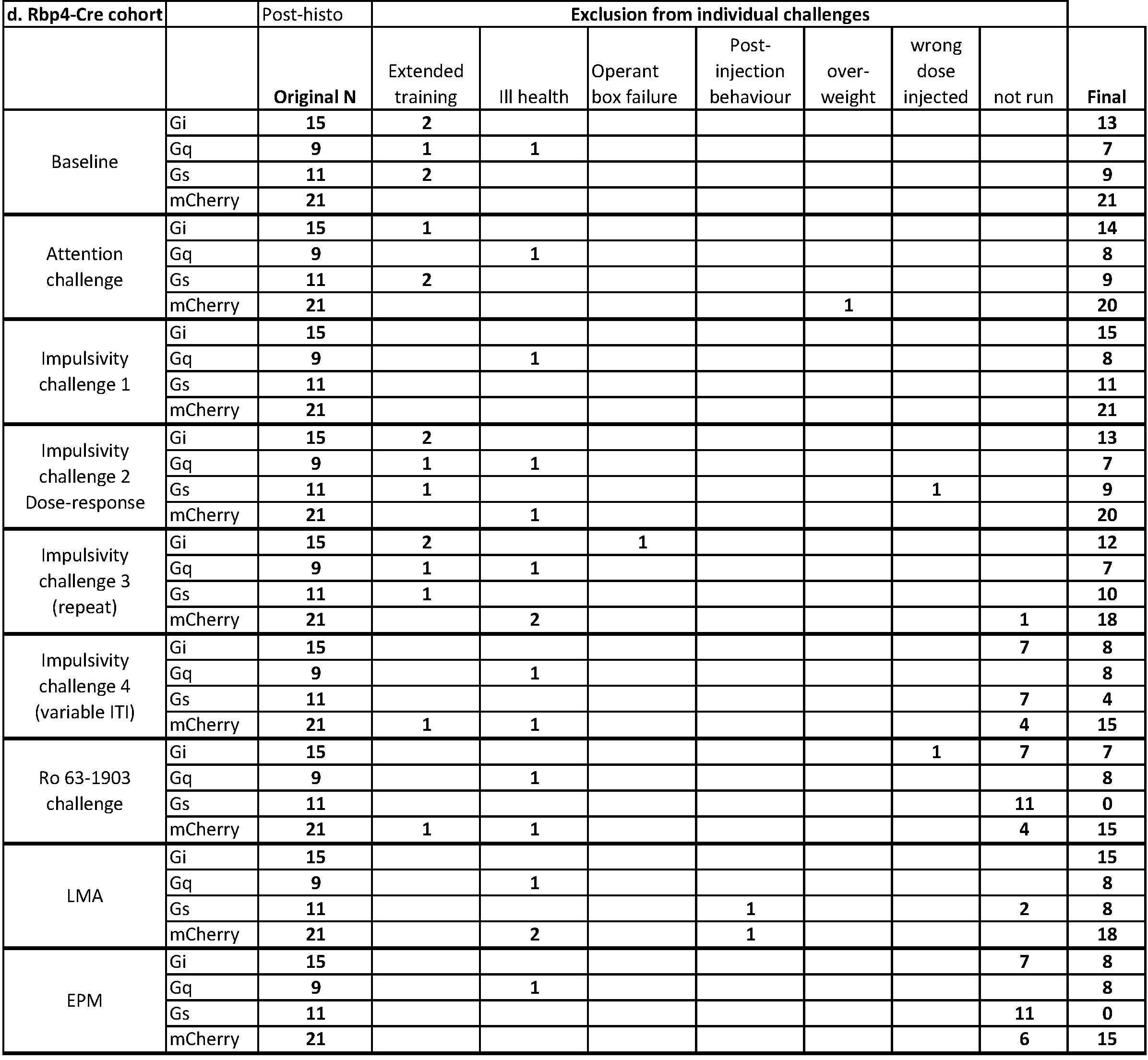


**Supplementary Table 3 | Reasons for exclusion of animals.** (**a**) Animals excluded post-hoc because of off-target expression in adjacent brain areas, or only unilateral or insufficient expression in the intended target region. (**b-c**) Animals of the CamKIIa-Cre (b), Tacr1-KO (c), or Rbp4-Cre (d) cohorts, that were part of the cohorts by histological criteria (stated as “Original N”) but did not contribute data to individual datasets for the stated reasons. Additionally, in (d), 2 Gi mice did not receive 10mg/kg in the vITI challenge.


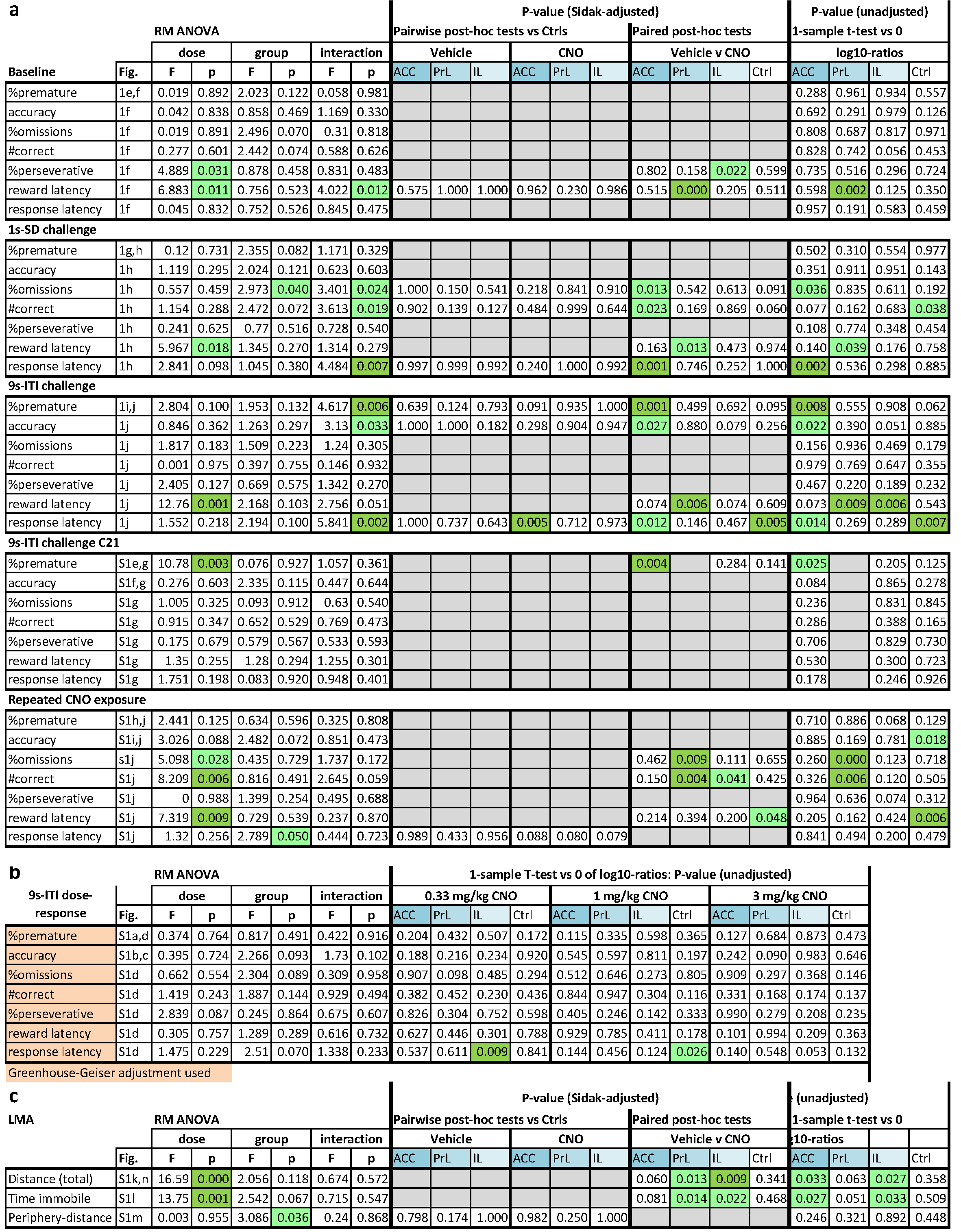


**Supplementary Table 4 | Statistics on behavioural experiments in CamKIIα-Cre mice.** (**a-c**) Results of repeated-measures ANOVA (left), pairwise between-subject and paired within-subject Sidak-adjusted simple-main effects post-hoc tests (middle) and unadjusted 1-sample *t*-tests on normalized parameter [log_10_(drug-value/vehicle-value), right] for the experiments shown in Fig. 1 and Supplementary Fig. 1 conducted in CamKIIα-Cre mice. All repeated-measures ANOVAs are two-way ANOVAs involving 1 within-subject parameter (agonist dose) and one between-subject parameter (subgroup, identified in the small tables on the left). Post-hoc tests that were not indicated to be run because of a lack of significant between-subject, within-subject or interaction effect in the overall RM-_ANOVA_ are omitted (grey cells). The figure that displays the statistically tested data is shown in the figure panel indicated in the “figure” column, and the statistically tested behavioural parameter and respective experiment (challenge protocol) are identified in the two left-most columns. The little tables on the left state the *N*-numbers for each subgroup. (**a**) 5-CSRTT tests with one dose of DREADD agonist. *N* (Baseline, 1s-SD challenge): 11 ACC, 14 PrL, 16 IL, 17 Ctrl. *N* (9s-ITI challenge): 11 ACC, 14 PrL, 15 IL, 17 Ctrl. *N* (9s-ITI challenge, C21): 11 ACC, 0 PrL, 9 IL, 11 Ctrl. *N* (repeated CNO): 10 ACC, 14 PrL, 15 IL, 14 Ctrl. (**b**) 5-CSRTT dose response test with 3 CNO doses in the 9s-ITI challenge. *N*: 11 ACC, 13 PrL, 15 IL, 13 Ctrl. (**c**) locomotor activity test. *N*: 10 ACC, 14 PrL, 15 IL, 15 Ctrl. See Supplementary Table 3 for reasons for exclusions and variations of *N*-numbers across experiments. *P*-values < 0.01 are highlighted in dark green, *P*-values < 0.05 in light green.


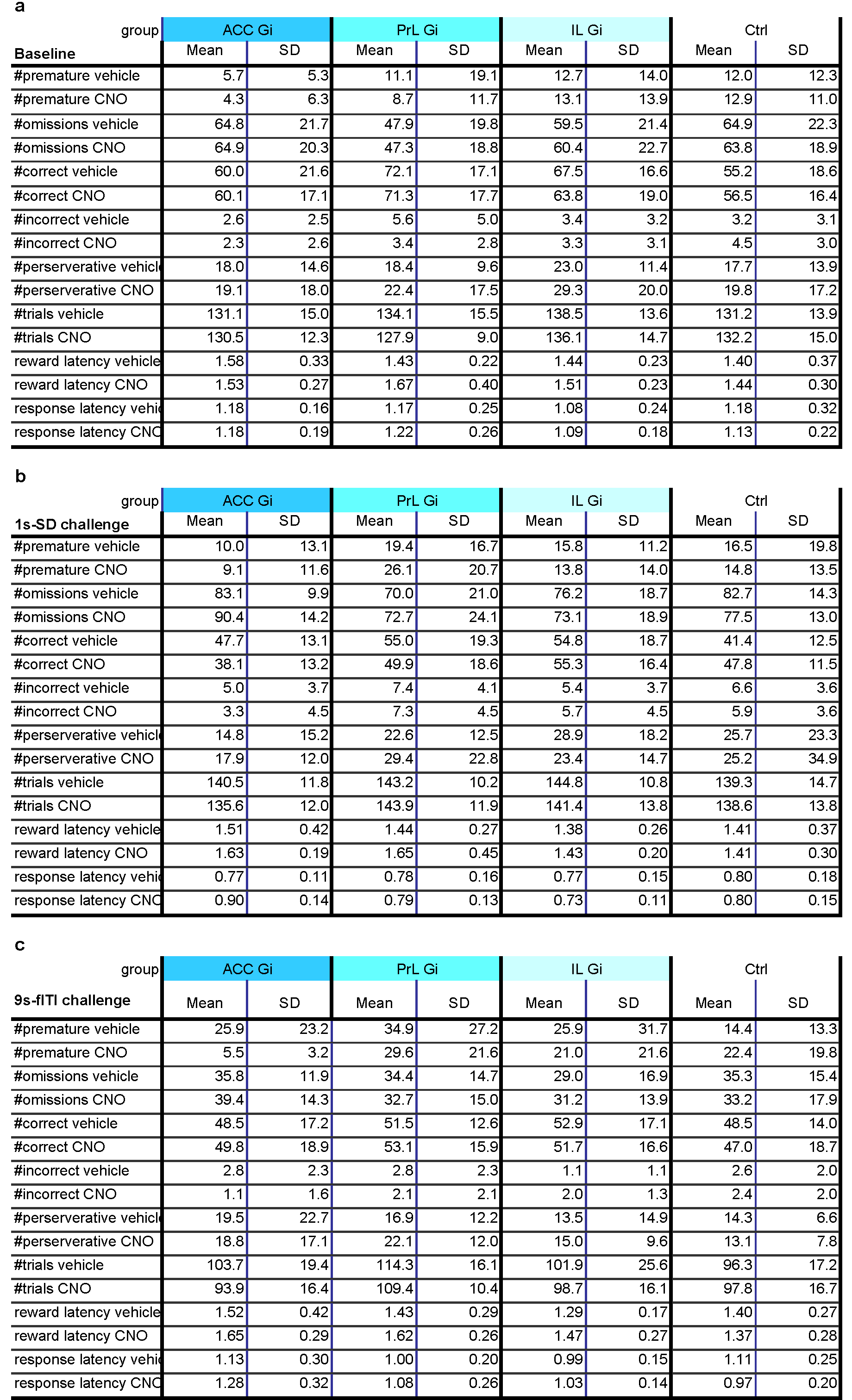


**Supplementary Table 5 | Absolute values in behavioural experiments in CamKIIα-Cre mice.** (**a-c**) The absolute number of all types of behavioural responses and latencies are shown for the three main testing conditions as displayed in Fig. 1, identified in the top left corner of each panel. SD, standard deviation.

**
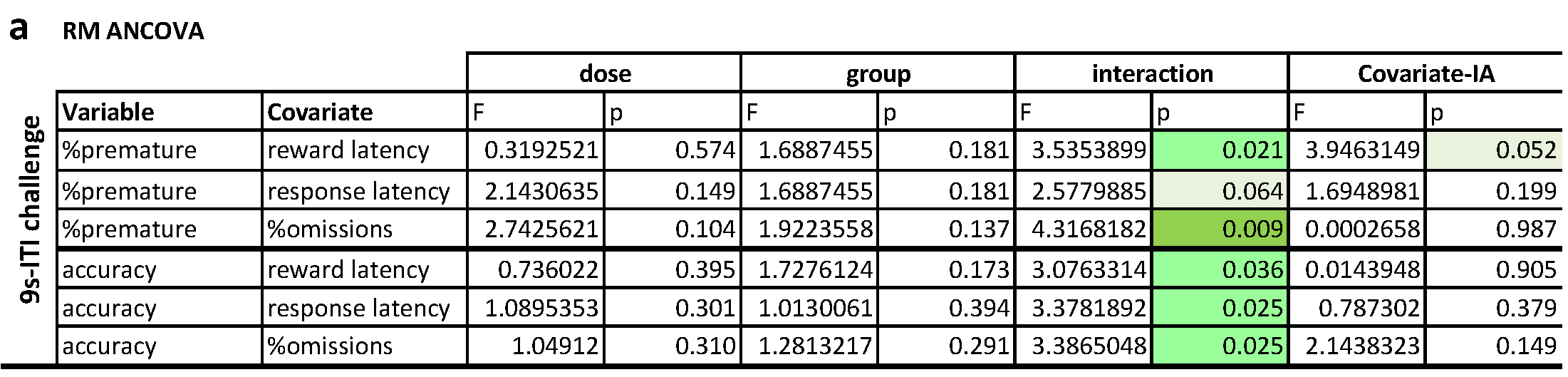
**


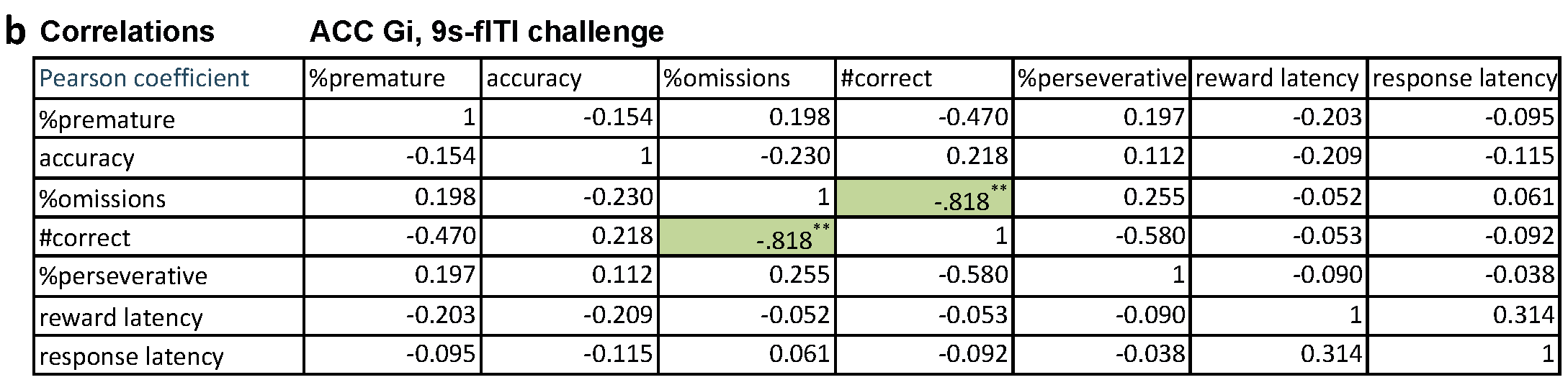


**Supplementary Table 6 | Co-variation between 5-CSRTT variables in CamKIIα-Cre mice in the 9s-fITI challenge.** (**a**) Repeated-measures ANCOVA for %prematures and accuracy in the first 9s-fITI-challenge (as analysed in Supplementary Table 4a with RM-ANOVA), using either relative reward latency, response latency, or %omissions as covariate. Covariates are log-transformed ratios of the value under CNO divided by the value under vehicle. (**b**) Bivariate Pearson correlation coefficients between 5-CSRTT variables within ACC-G_i_ group in the 9s-fITI-challenge. Values used for correlations are log-transformed ratios of the value under CNO divided by the value under vehicle. ** *P*-values < 0.01 highlighted in dark green, * *P*-values < 0.05 in light green.


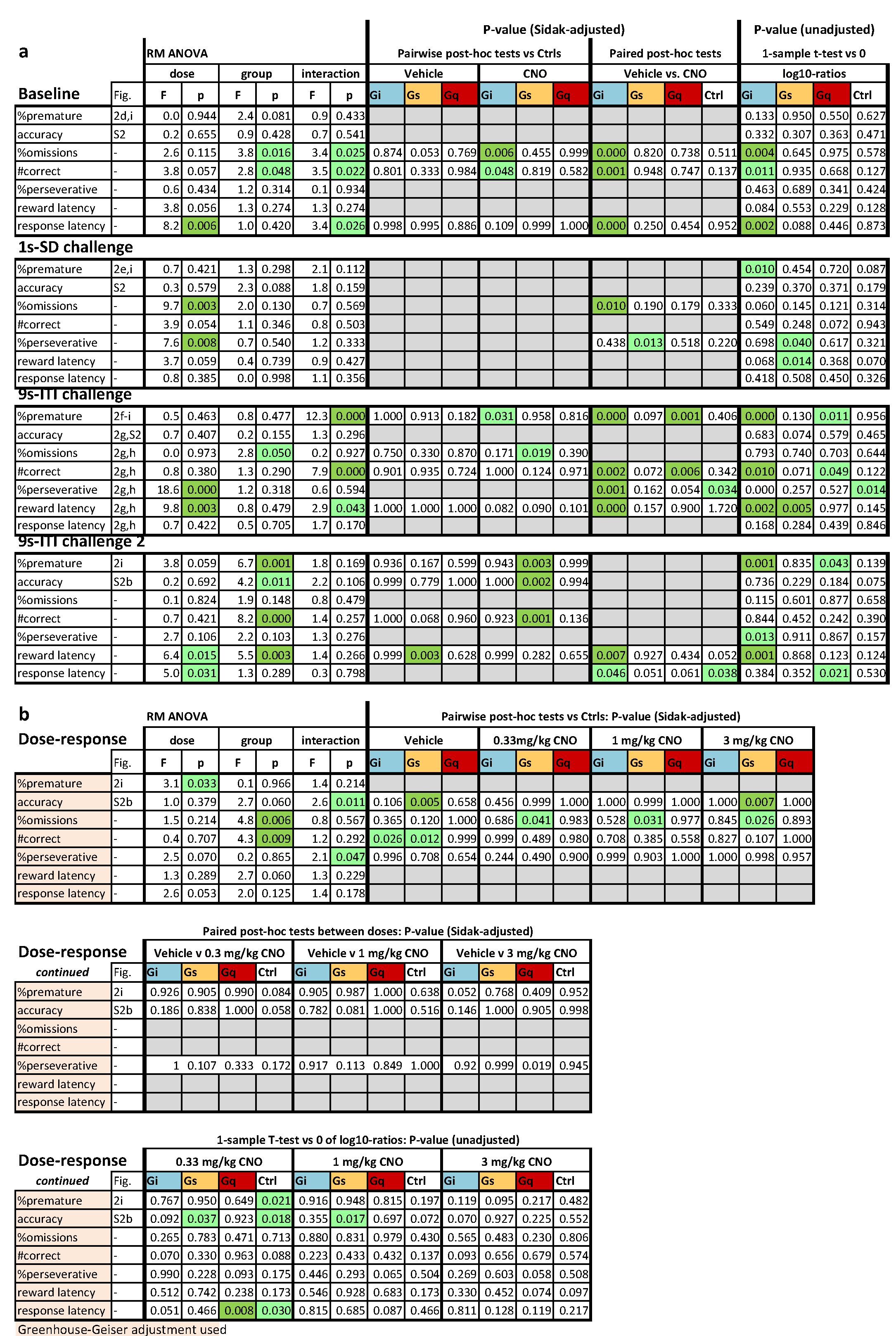


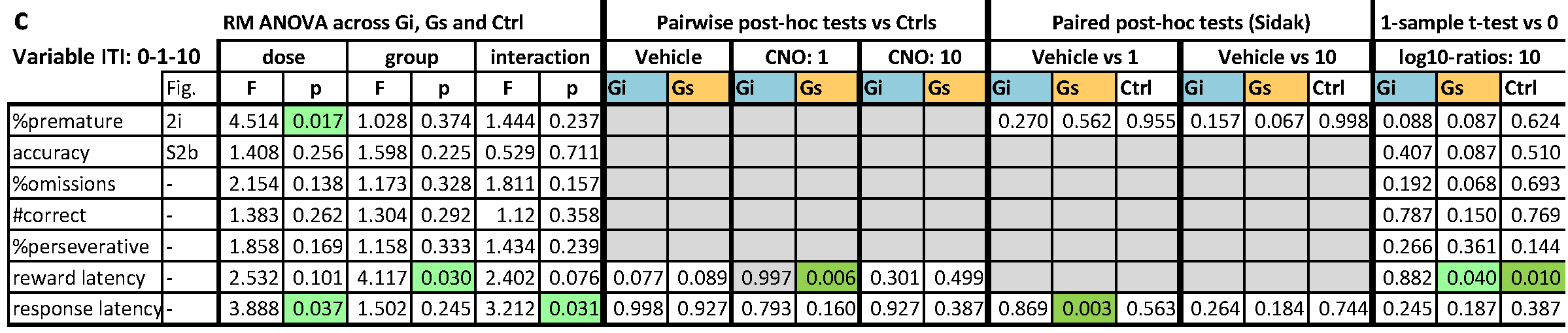


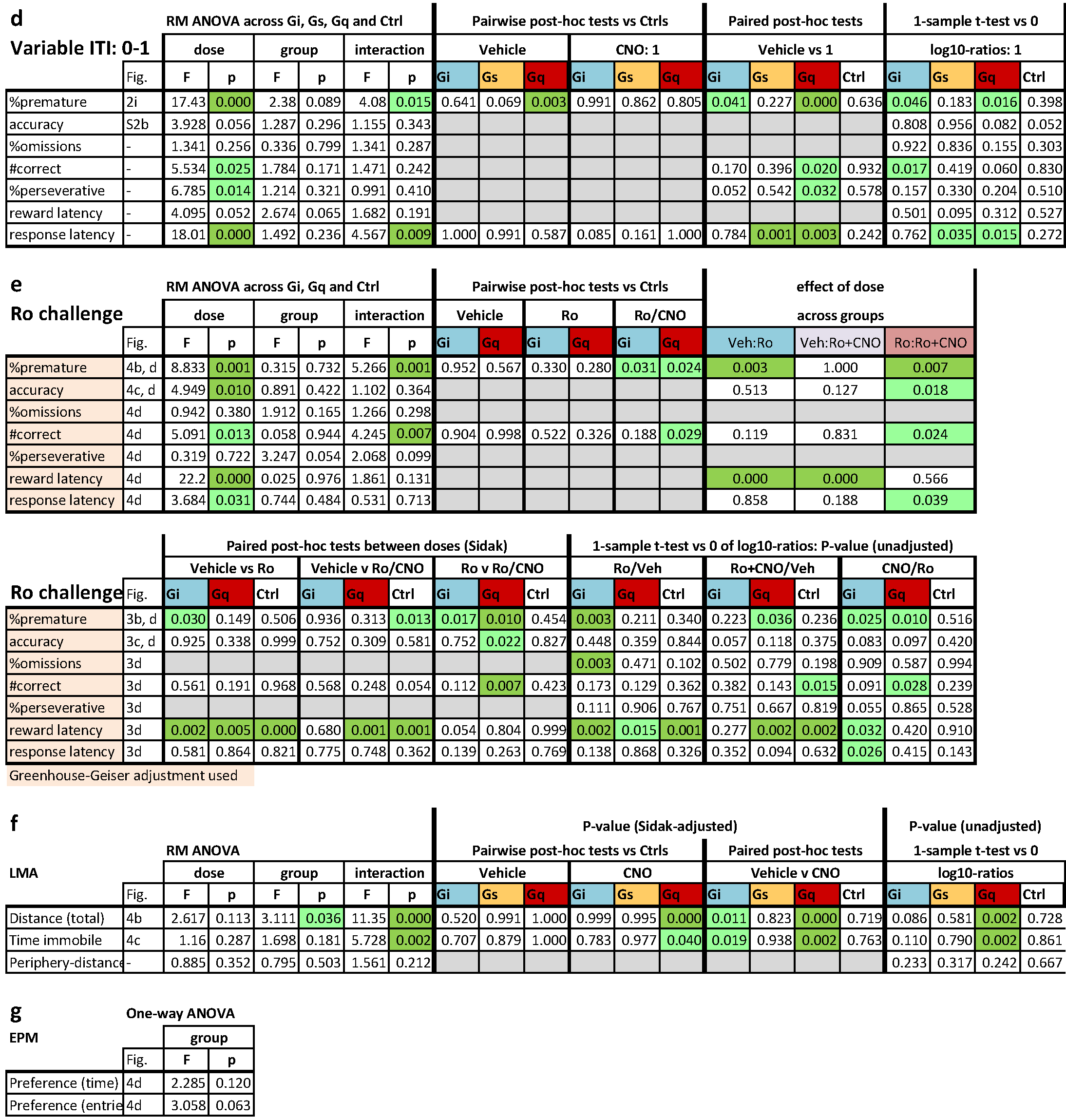


**Supplementary Table 7 | Statistics on behaviour in Rbp4-Cre mice.** Results of repeated-measures ANOVA (left), pairwise between-subject and paired within-subject Sidak-adjusted simple-main effects post-hoc tests (middle) and unadjusted 1-sample *t*-tests on normalized parameter [log_10_(drug-value/vehicle-value), right] for the chemogenetic experiments shown in Fig.2 and Supplementary Fig. 2 conducted in Rbp4-Cre mice. All repeated-measures ANOVAs are two-way ANOVAs involving 1 within-subject parameter (CNO dose) and one between-subject parameter (subgroup, identified in the small tables on the left). Post-hoc tests that were not indicated to be run because of a lack of significant between-subject, within-subject or interaction effect in the overall RM-ANOVA are omitted (grey cells). The figure that displays the statistically tested data is shown in the figure panel indicated in the “figure” column, and the statistically tested behavioural parameter and respective experiment (challenge protocol) are identified in the top-left corner of each section. (**a**) 5-CSRTT tests with one CNO dose, *N*: 13 Gi, 9 Gs, 7 Gq, 21 Ctrl. (**b**) 5-CSRTT dose response test with 3 CNO doses, *N*: 13 Gi, 9 Gs, 7 Gq, 20 Ctrl. (**c**) variable ITI challenge with vehicle and 2 doses of CNO (1 and 10 mg/kg) in the ACC-Gi and Gs groups, *N*: 6 Gi, 4 Gs, 15 Ctrl. (**d**) same data as in (c) but analysed only for vehicle and 1 mg/kg CNO to include the ACC-Gq group that did not receive 10 mg/kg CNO, *N*: 8 Gi, 4 Gs, 8 Gq, 15 Ctrl. (**e**) Ro 63-1903 challenge (data for G_s_-mice is excluded because this group contributes only an *N* = 3 to this test), *N*: 7 Gi, 8 Gq, 16 Ctrl. (**f**) Locomotor activity test, *N*: 15 Gi, 8 Gs, 8 Gq, 18 Ctrl. (**g**) Elevated-plus-maze test, *N*: 8 Gi, 8 Gq, 15 Ctrl. See Supplementary Table 3 for reasons for exclusions and variations of *N*-numbers across experiments. *P*-values < 0.01 are highlighted in dark green, *P*-values < 0.05 in light green.


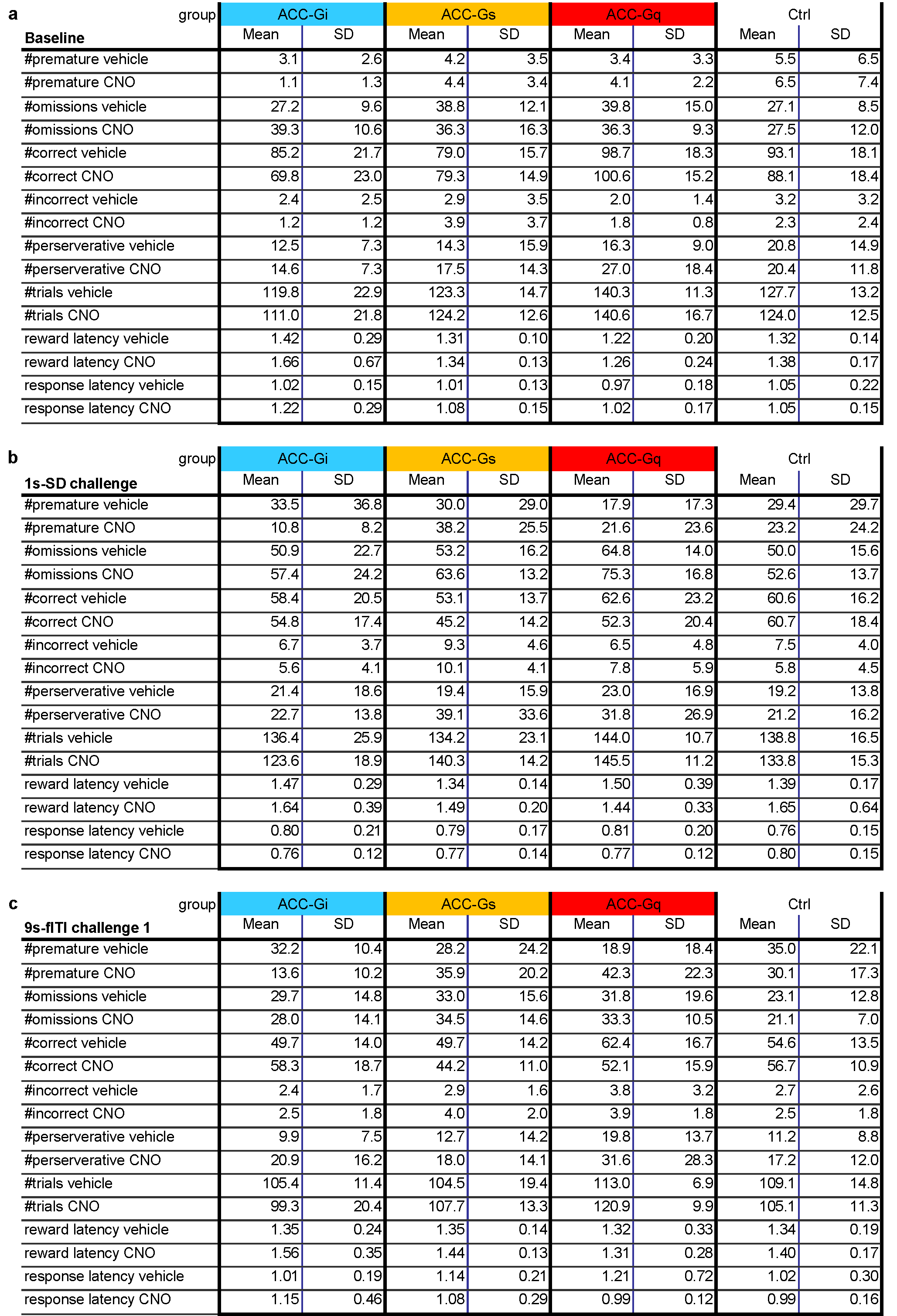


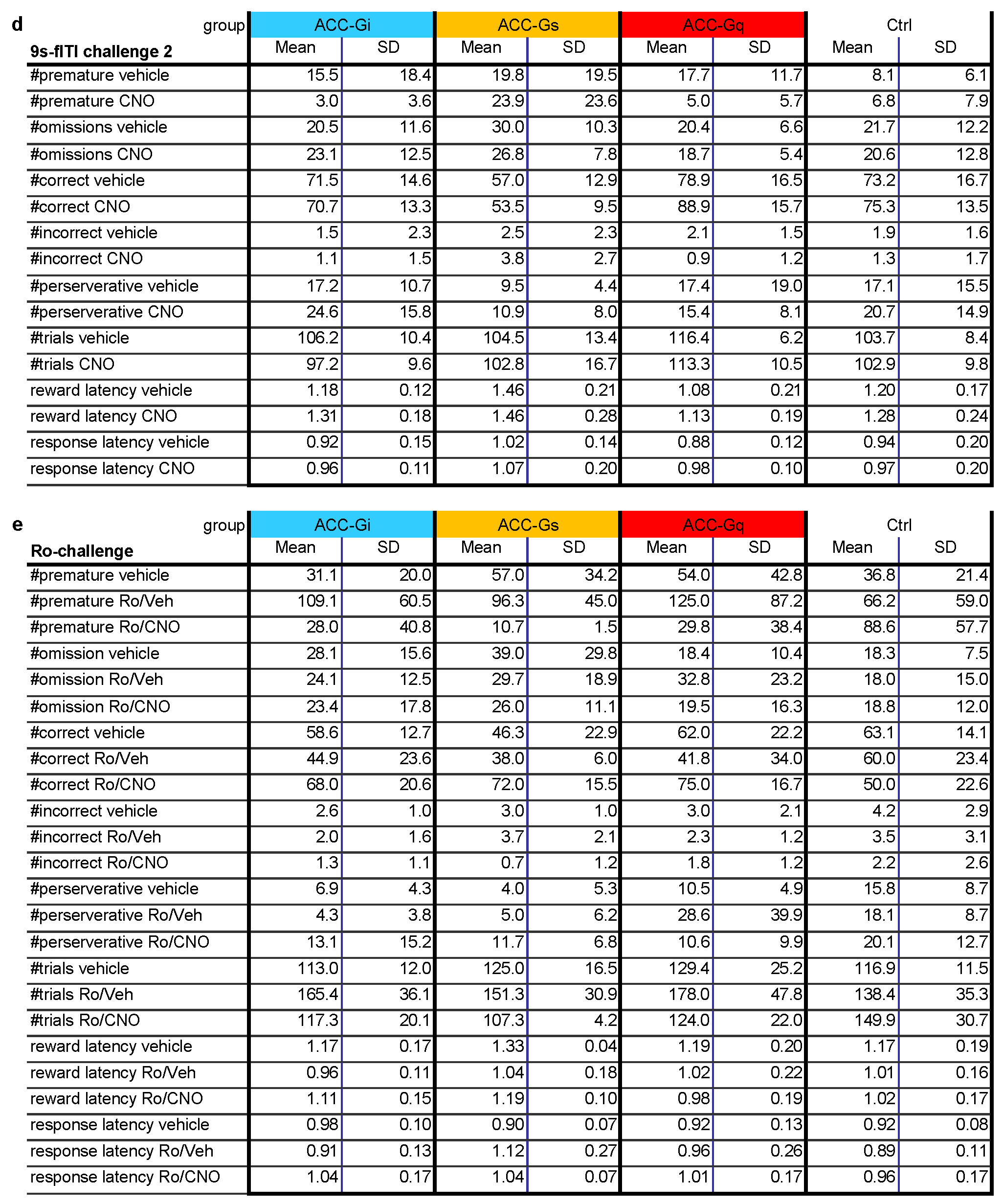


**Supplementary Table 8 | Absolute values in behavioural experiments in Rbp4-Cre mice.** (**a-e**) The absolute number of all types of behavioural responses and latencies are shown for the five main testing conditions done in the Rbp4-Cre cohort as displayed in Fig. 2 and 3, and identified in the top left corner of each panel. SD, standard deviation.


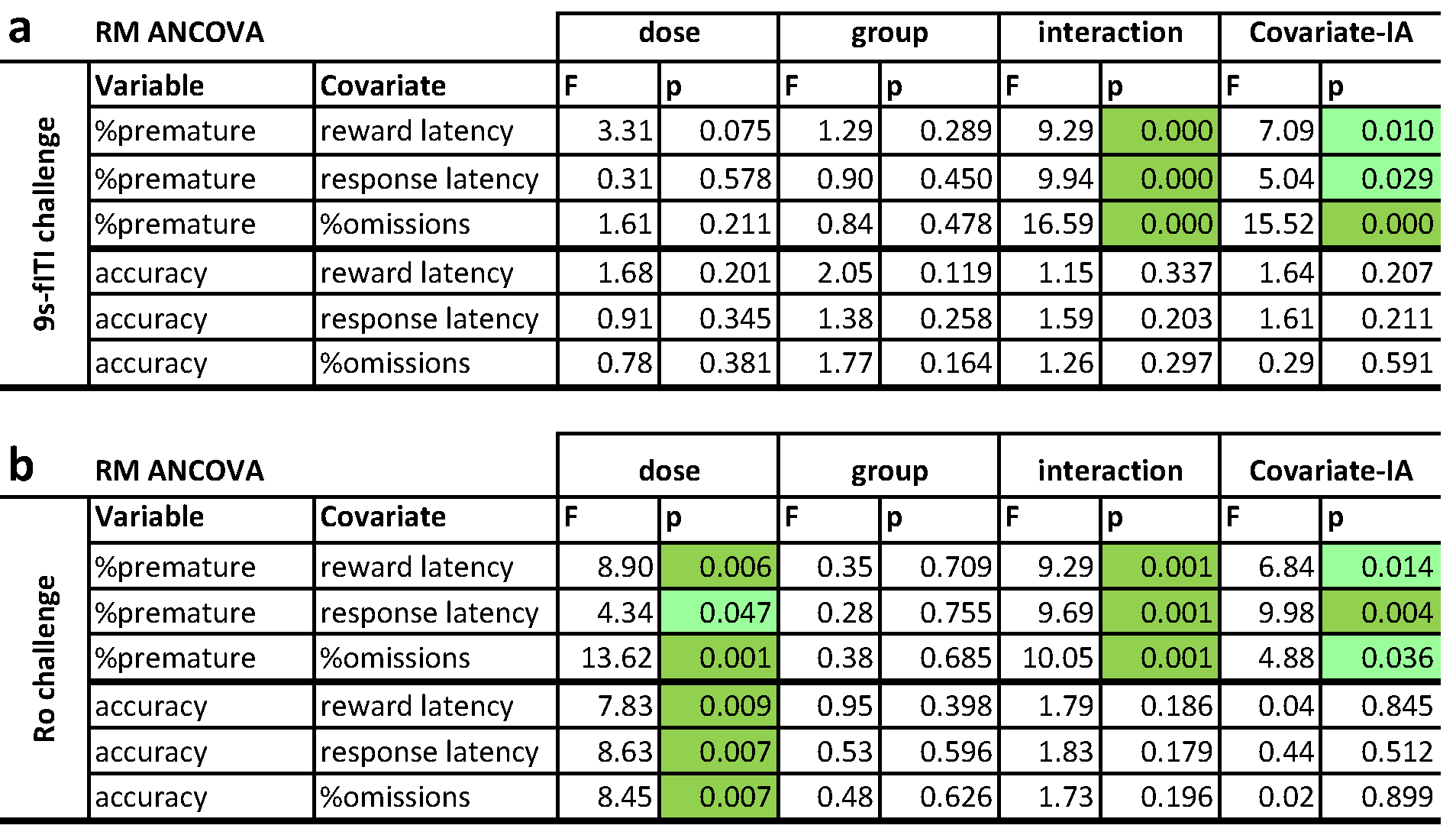


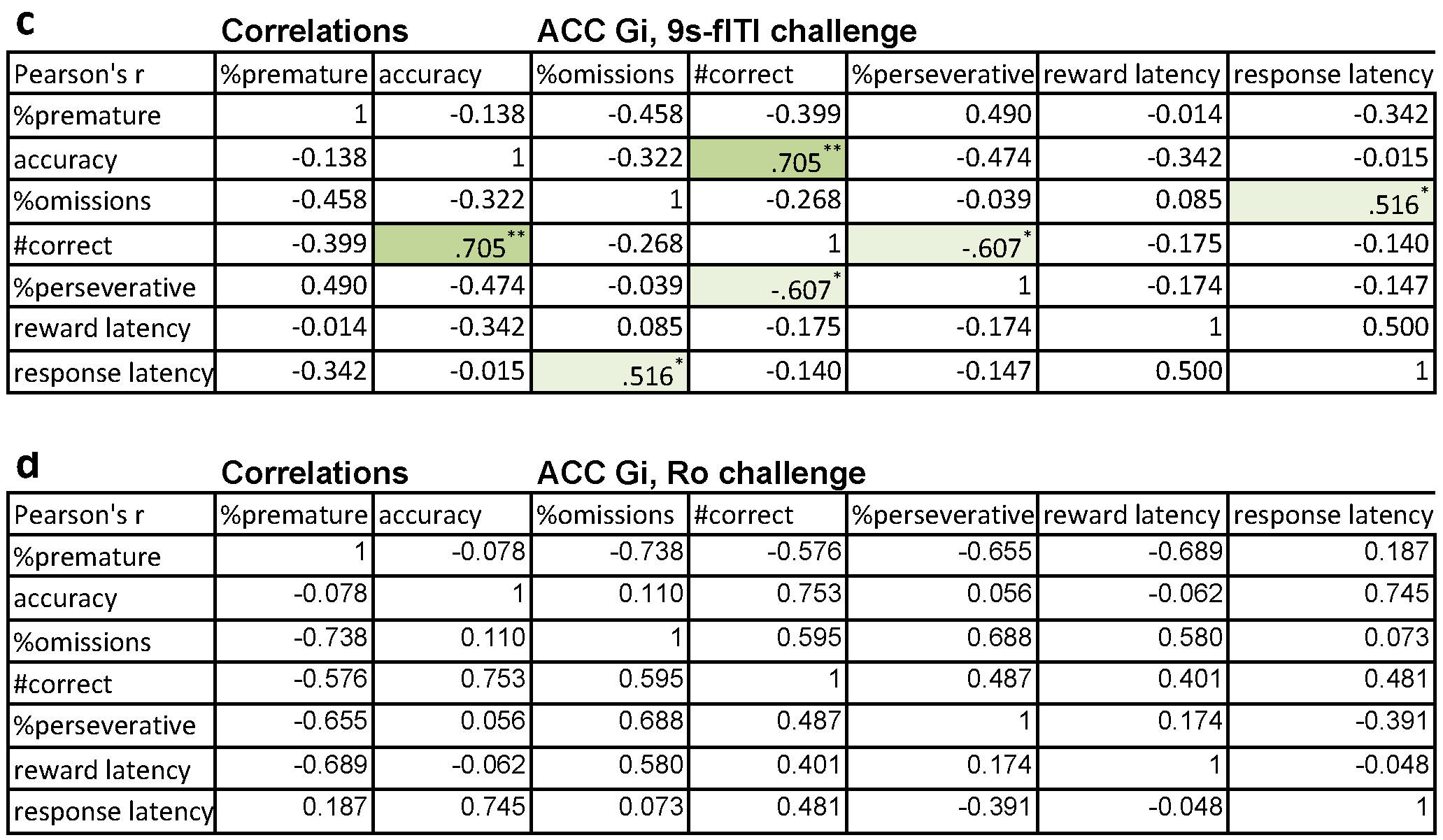


**Supplementary Table 9 | Co-variation between 5-CSRTT variables in Rbp4-Cre mice in the 9s-fITI and Ro challenges.** (**a**) Repeated-measures ANCOVA for %prematures and accuracy in the first 9s-fITI-challenge (as analysed in Supplementary Table 7a with RM-ANOVA), using either relative reward latency, response latency, or %omissions as covariate. Covariates are log-transformed ratios of the value under CNO divided by the value under vehicle. (**b**) Same as (a) but for Ro-challenge (as analysed with ANOVA in Supplementary Table 7e) but including only the values under Ro/vehicle and Ro/CNO as dependent variables. (**c-d**) Bivariate Pearson correlation coefficients between 5-CSRTT variables within ACC-G_i_ group in the 9s-fITI-challenge (c) and the Ro-challenge (d). Values used for correlations are log-transformed ratios of the value under CNO divided by the value under vehicle.


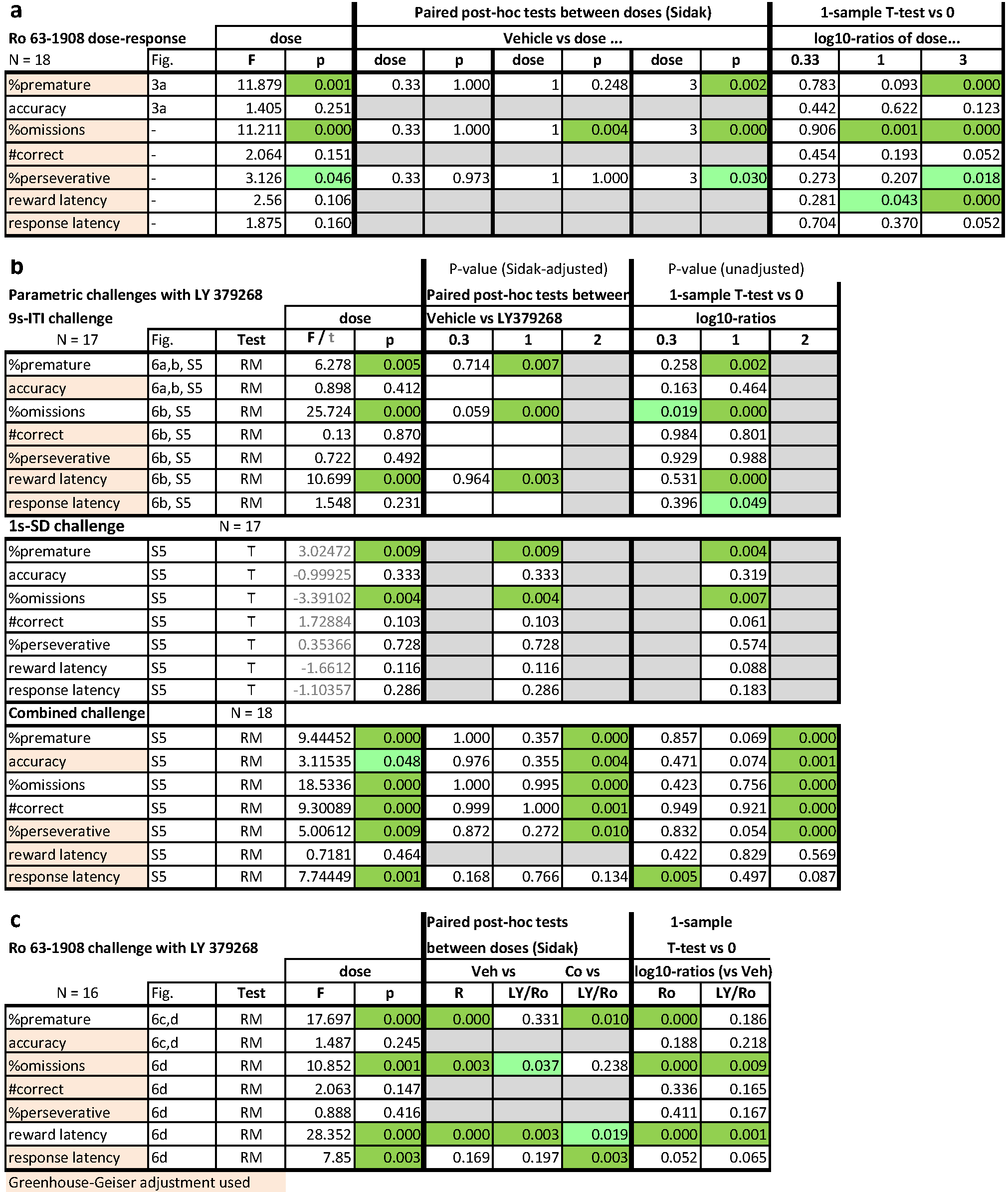


**Supplementary Table 10 | Statistics on LY379268 and Ro 63-1908 experiments in wildtype mice.** Results of repeated-measures ANOVA (left), paired within-subject Sidak-adjusted simple-main effects post-hoc tests (middle) and unadjusted 1-sample *t*-tests on normalized parameter [log_10_(drug-value/vehicle-value), right] for the pharmacological experiments with Ro 63-1908 and LY379268 shown in Fig. 3a and 6a-d and conducted in wildtype mice. All repeated-measures ANOVAs are one-way RM-ANOVAs involving 1 within-subject parameter (drug dose). Post-hoc tests that were not indicated to be run because of a lack of significant between-subject, within-subject or interaction effect in the overall RM-ANOVA are omitted (grey cells). The figure that displays the statistically tested data is shown in the figure panel indicated in the “figure” column, and the statistically tested behavioural parameter and respective experiment (challenge protocol), including *N*-numbers are identified above each table section. (**a**) Dose-response curve analysis for Ro 63-1908. (**b**) Dose-response experiments in parametric 5-CSRTT challenges (note that in the 1 s SD attention challenge only one dose was tested, hence a paired *t*-test is used for analysis). One mouse of the full cohort (*N* = 18) was not run in the 9 s fITI challenge, because it had not met the required criterion on the baseline stage (stage 5) by the time of this experiment; another mouse did not contribute data to the 1 s SD attention challenge because of an electronic problem of the operant box. (**c**) Experiment of LY379268 activity in mice challenged with Ro 63-1908. Two mice were excluded from this dataset – one did not respond to the Ro 63-1908 challenge in the initial dose-response curve (experiment analysed in panel (a) and Fig. 3a), another one had part of the injected i.p. bolus leaking back out on two of the three test days. *P*-values < 0.01 are highlighted in dark green, *P*-values < 0.05 in light green.


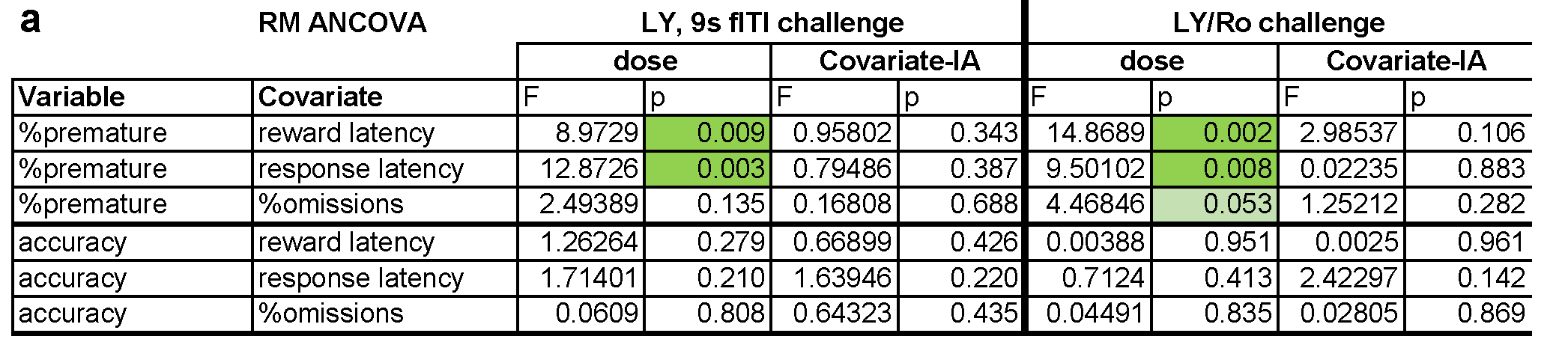


**
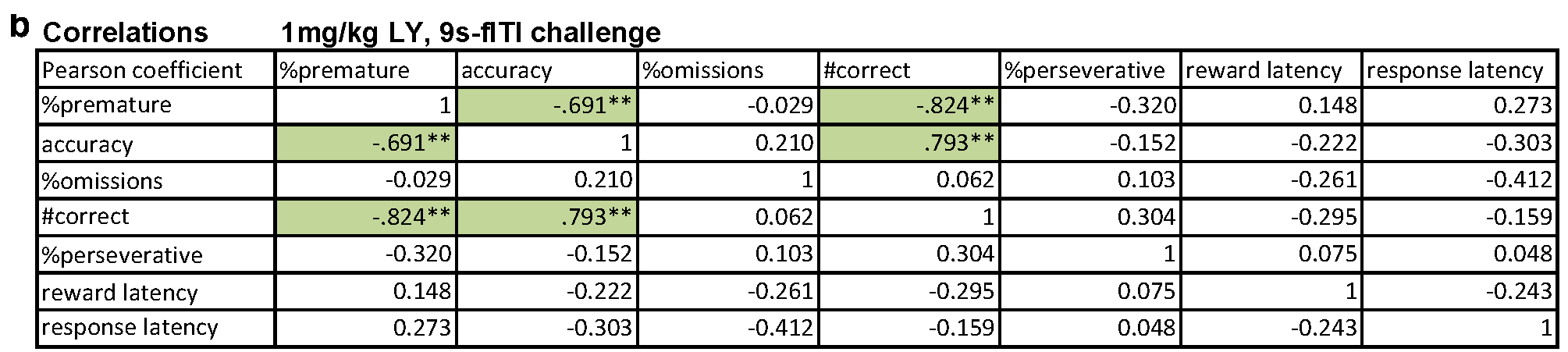
**

**
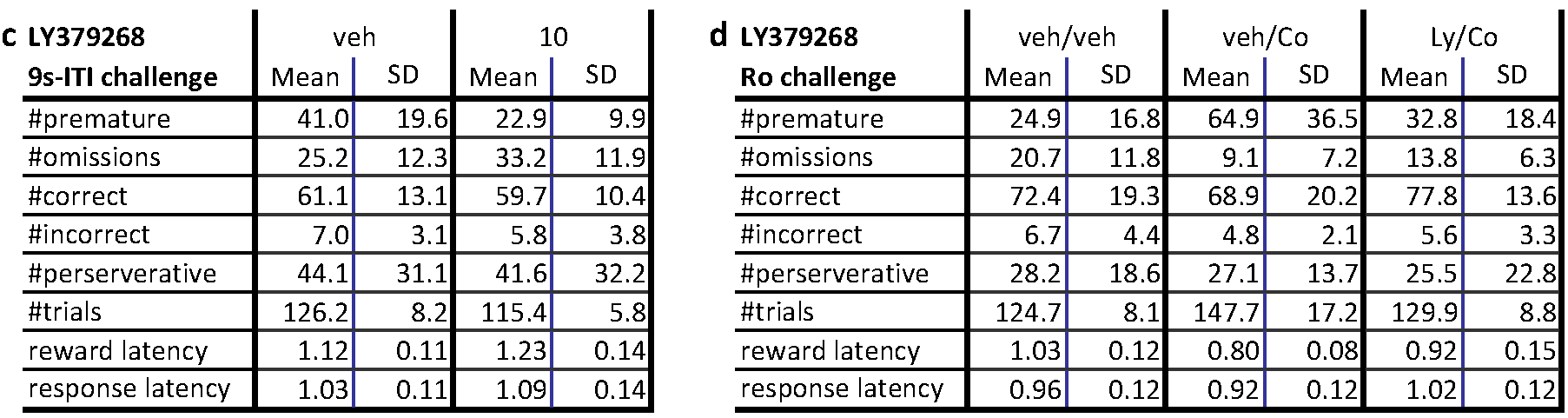
**

**Supplementary Table 11 | Co-variation between 5-CSRTT variables in mice treated with LY379268 and absolute values of response parameters.** (**a**) Repeated-measures ANCOVA for %prematures and accuracy in the 9s-fITI-challenge, left, and Ro-challenge, right, after LY379268 treatment (as analysed in Supplementary Table 10b,c with RM-ANOVA), using either relative reward latency, response latency, or %omissions as covariate. Covariates are log-transformed ratios of the value under LY (or LY/Ro) divided by the value under vehicle (vehicle/Ro). (**b**) Bivariate Pearson correlation coefficients between 5-CSRTT variables in WT mice in the 9s-fITI-challenge. Values used for correlations are log-transformed ratios of the value under 1 mg/kg LY divided by the value under vehicle. ** *P*-values < 0.01 highlighted in dark green, * *P*-values < 0.05 in light green. (**c, d**) Absolute number of all types of behavioural responses and latencies are shown for the 9s-fITI challenge (c) and the Ro-challenge (d) evaluated with LY pre-treatment. SD, standard deviation.


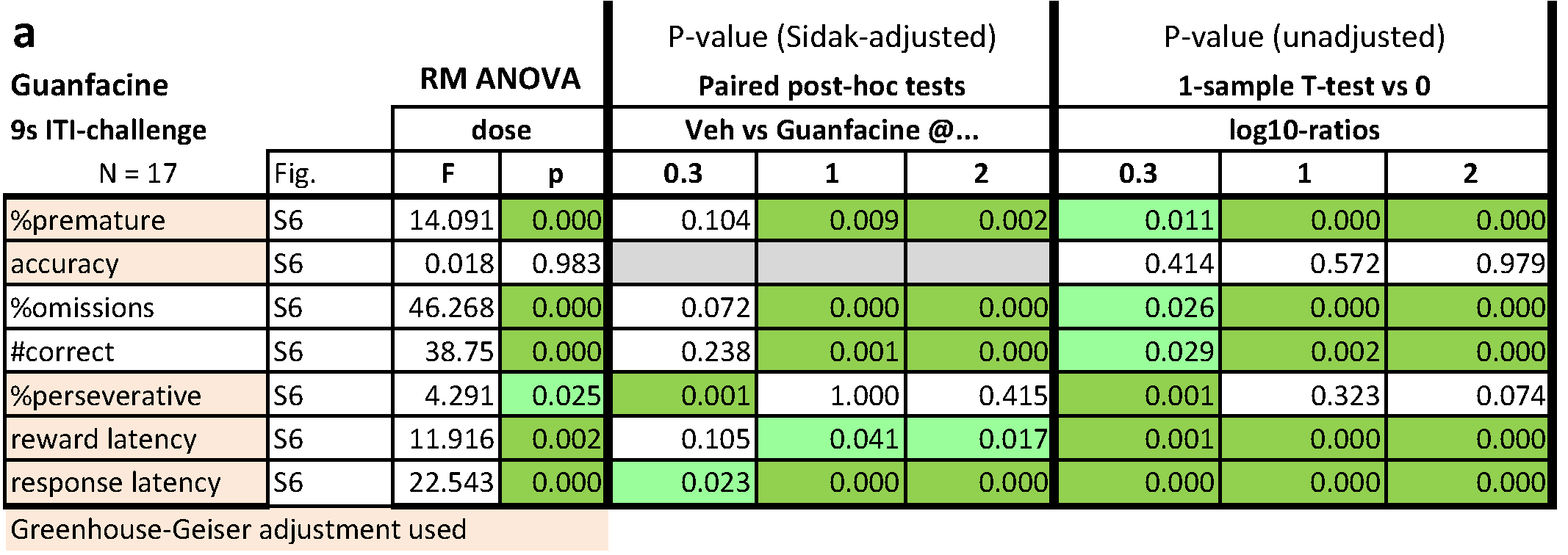


**
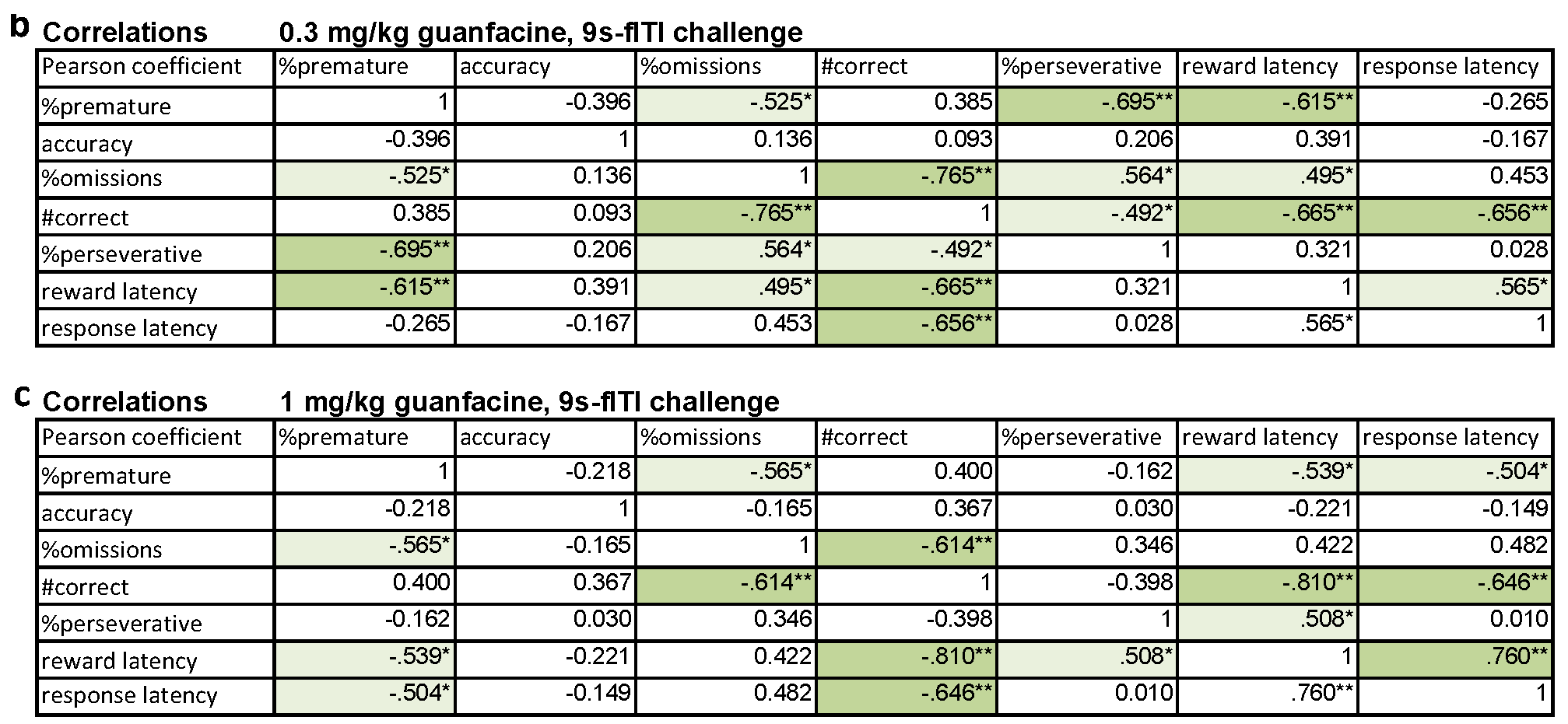
**

**Supplementary Table 12 | Statistics on guanfacine experiments in wildtype mice.** Results of repeated-measures ANOVA (left), paired within-subject Sidak-adjusted simple-main effects post-hoc tests (middle) and unadjusted 1-sample *t*-tests on normalized parameter [log_10_(drug-value/vehicle-value), right] for the pharmacological experiments with guanfacine (gua) shown in Supplementary Fig. 6, and conducted in wildtype mice. All repeated-measures ANOVAs are one-way RM-ANOVAs involving 1 within-subject parameter (drug dose). Post-hoc tests that were not indicated to be run because of a lack of significant between-subject, within-subject or interaction effect in the overall RM-ANOVA are omitted (grey cells). The figure that displays the statistically tested data is shown in the figure panel indicated in the “figure” column, and the statistically tested behavioural parameter and respective experiment (challenge protocol), including *N*-numbers are identified in the two left-most columns. (**a**) Dose-response experiment with guanfacine in 9s-fITI 5-CSRTT challenge. (**b-c**) Bivariate Pearson correlation coefficients between 5-CSRTT variables in WT-mice in the 9s-fITI-challenge. Values used for correlations are log-transformed ratios of the value under 0.3 (b) and 1 mg/kg LY (c) divided by the value under vehicle. ** *P*-values < 0.01 highlighted in dark green, * *P*-values < 0.05 in light green.


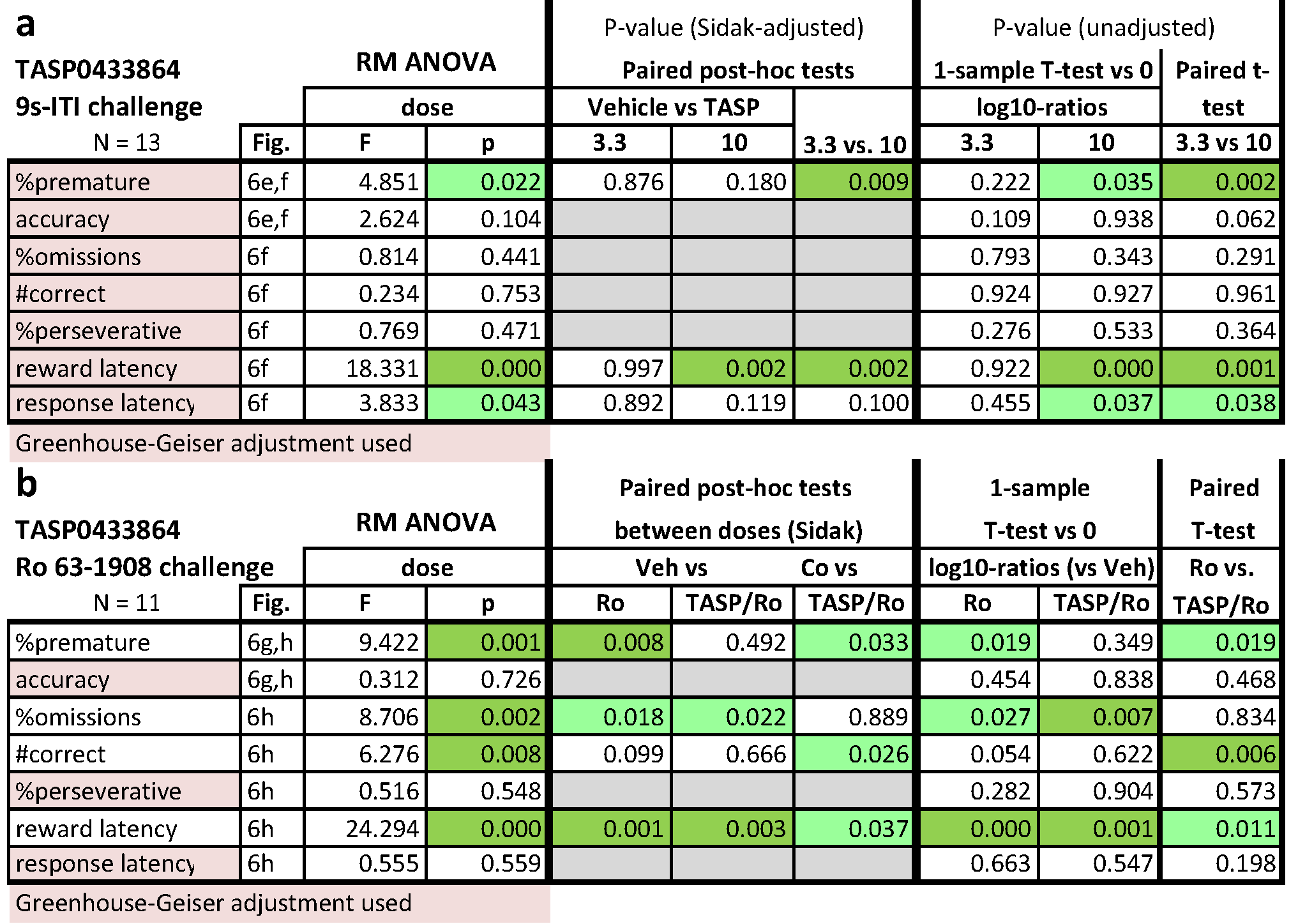


**
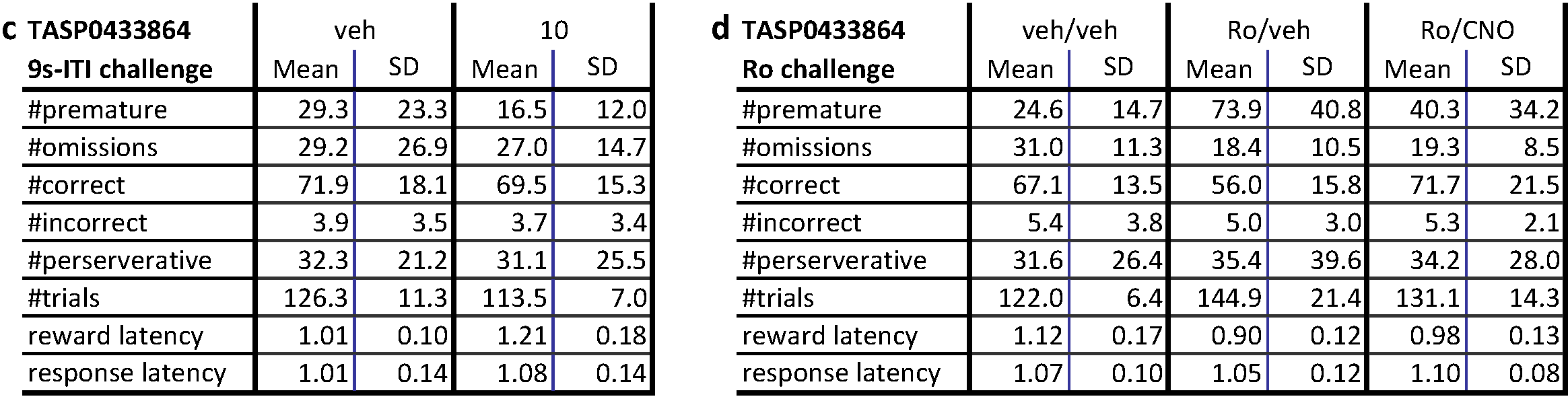
**

**Supplementary Table 13 | Statistics and absolute parameters of TASP experiments in wildtype mice.** Results of repeated-measures ANOVA (left), paired within-subject Sidak-adjusted simple-main effects post-hoc tests (middle) and unadjusted 1-sample *t*-tests on normalized parameter [log_10_(drug-value/vehicle-value), right] for the pharmacological experiments with TASP shown in Fig. 6 (e-h), and conducted in wildtype mice. All repeated-measures ANOVAs are one-way RM-ANOVAs involving 1 within-subject parameter (drug dose). Post-hoc tests that were not indicated to be run because of a lack of significant between-subject, within-subject or interaction effect in the overall RM-ANOVA are omitted (grey cells). The figure that displays the statistically tested data is shown in the figure panel indicated in the “figure” column, and the statistically tested behavioural parameter and respective experiment (challenge protocol), including *N*-numbers are identified in the two left-most columns. (**a**) Dose-response experiment with TASP in parametric 5-CSRTT challenge. (**b**) Experiment of effect of 10 mg/kg TASP in mice challenged with Ro 63-1908. One mouse of the original cohort (*N* = 13) was excluded because Ro came partially back out after the injection, another mouse was excluded because the operant box was malfunctioning. *P*-values < 0.01 are highlighted in dark green, *P*-values < 0.05 in light green. (**c, d**) Absolute number of all types of behavioural responses and latencies are shown for the two testing conditions evaluated statistically in (a, b) as identified in the top left corner of each panel. SD, standard deviation.


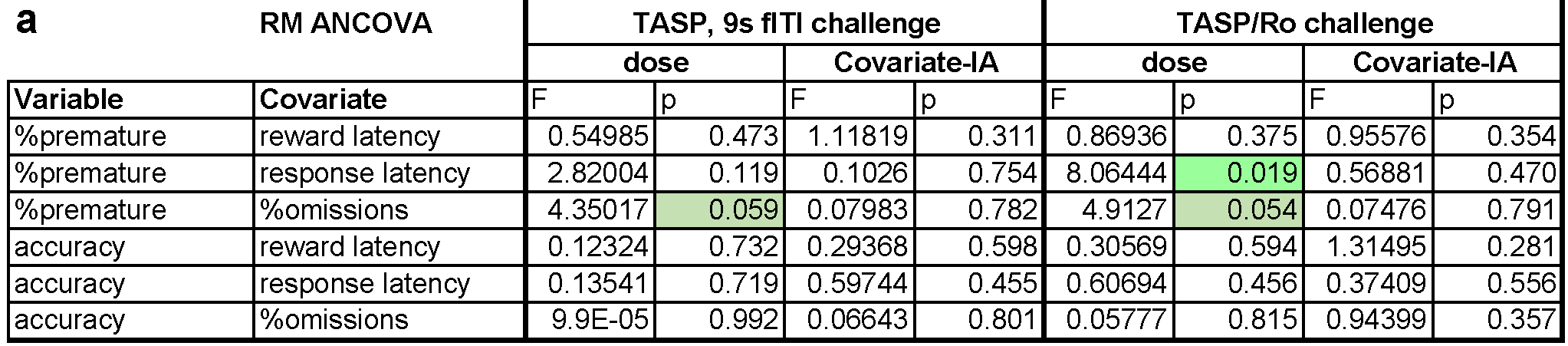


**Supplementary Table 14 | Co-variation between 5-CSRTT variables in mice treated with TASP.** (**a**) Repeated-measures ANCOVA for %prematures and accuracy in the 9s-fITI-challenge, left, and Ro-challenge, right, after TASP treatment (as analysed in Supplementary Table 13a,b with RM-ANOVA), using either relative reward latency, response latency, or %omissions as covariate. Covariates are log-transformed ratios of the value under TASP (or TASP/Ro) divided by the value under vehicle (or vehicle/Ro).


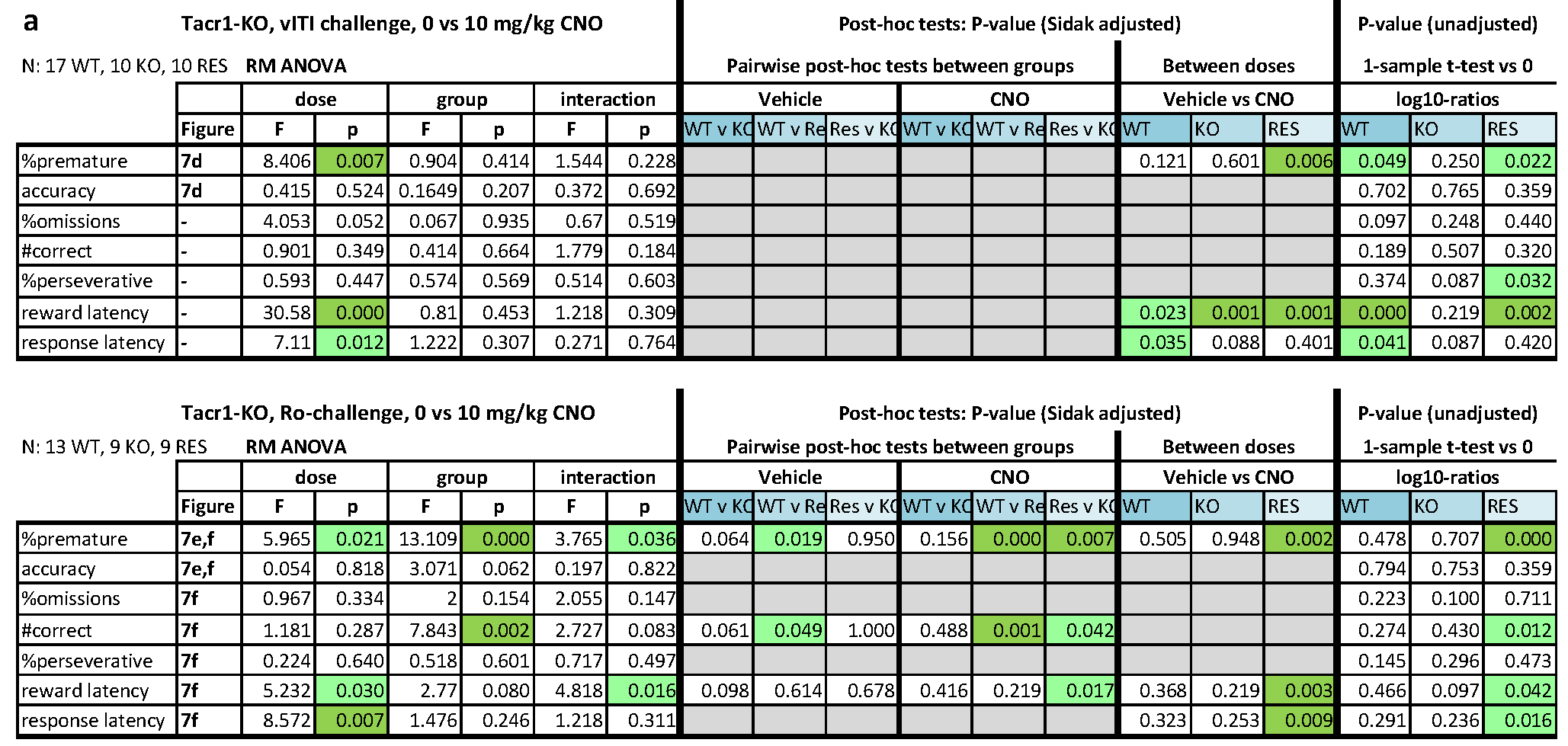

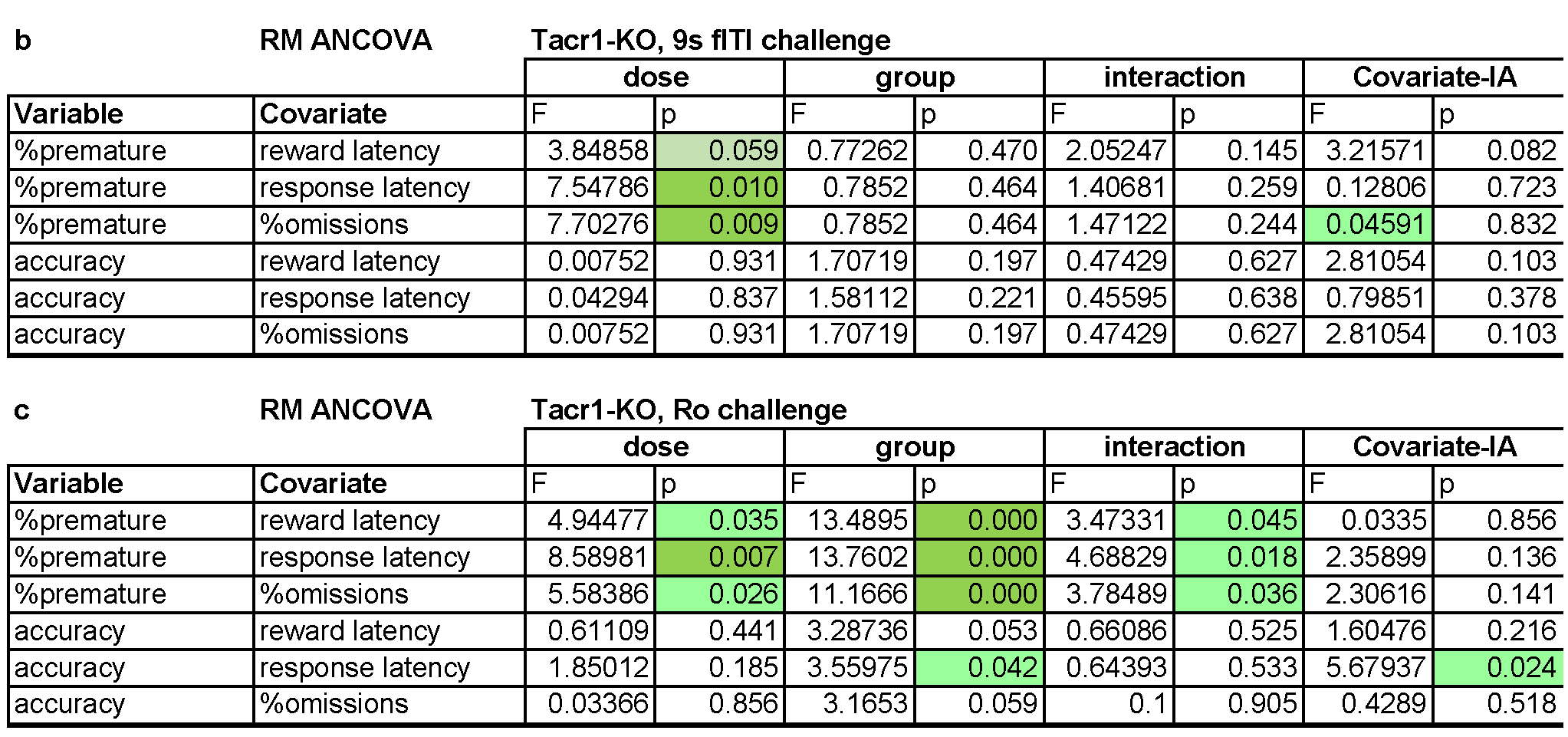


**Supplementary Table 15 | Statistics on behavioural experiments in *Tacr1*-KO mice.** (**a**) Results of repeated-measures ANOVA (left), pairwise between-subject and paired within-subject Sidak-adjusted simple-main effects post-hoc tests (middle) and unadjusted 1-sample *t*-tests on normalized parameter [log_10_(CNO-value/vehicle-value), right] for the chemogenetic experiments shown in Fig. 7 and conducted in *Tacr1*-KO mice. All repeated-measures ANOVAs are two-way ANOVAs involving 1 within-subject parameter (CNO dose) and one between-subject parameter (subgroup, identified in the small tables on the left). Post-hoc tests that were not indicated to be run because of a lack of significant between-subject, within-subject or interaction effect in the overall RM-ANOVA are omitted (grey cells). The figure that displays the statistically tested data is shown in the figure panel indicated in the “figure” column, and the statistically tested behavioural parameter and respective experiment (challenge protocol) are identified in the two left-most columns. The little tables on the left state the *N*-numbers for each subgroup. (**b,c**) Repeated-measures ANCOVA for %prematures and accuracy in the 9s-fITI-challenge (b) and Ro-challenge (c) after CNO treatment (as analysed in Table 15a with RM-ANOVA), using either relative reward latency, response latency, or %omissions as covariate. Covariates are log-transformed ratios of the value under CNO divided by the value under vehicle. See Supplementary Table 3 for reasons for exclusions and variations of *N*-numbers across experiments. *P*-values < 0.01 are highlighted in dark green, *P*-values < 0.05 in light green.


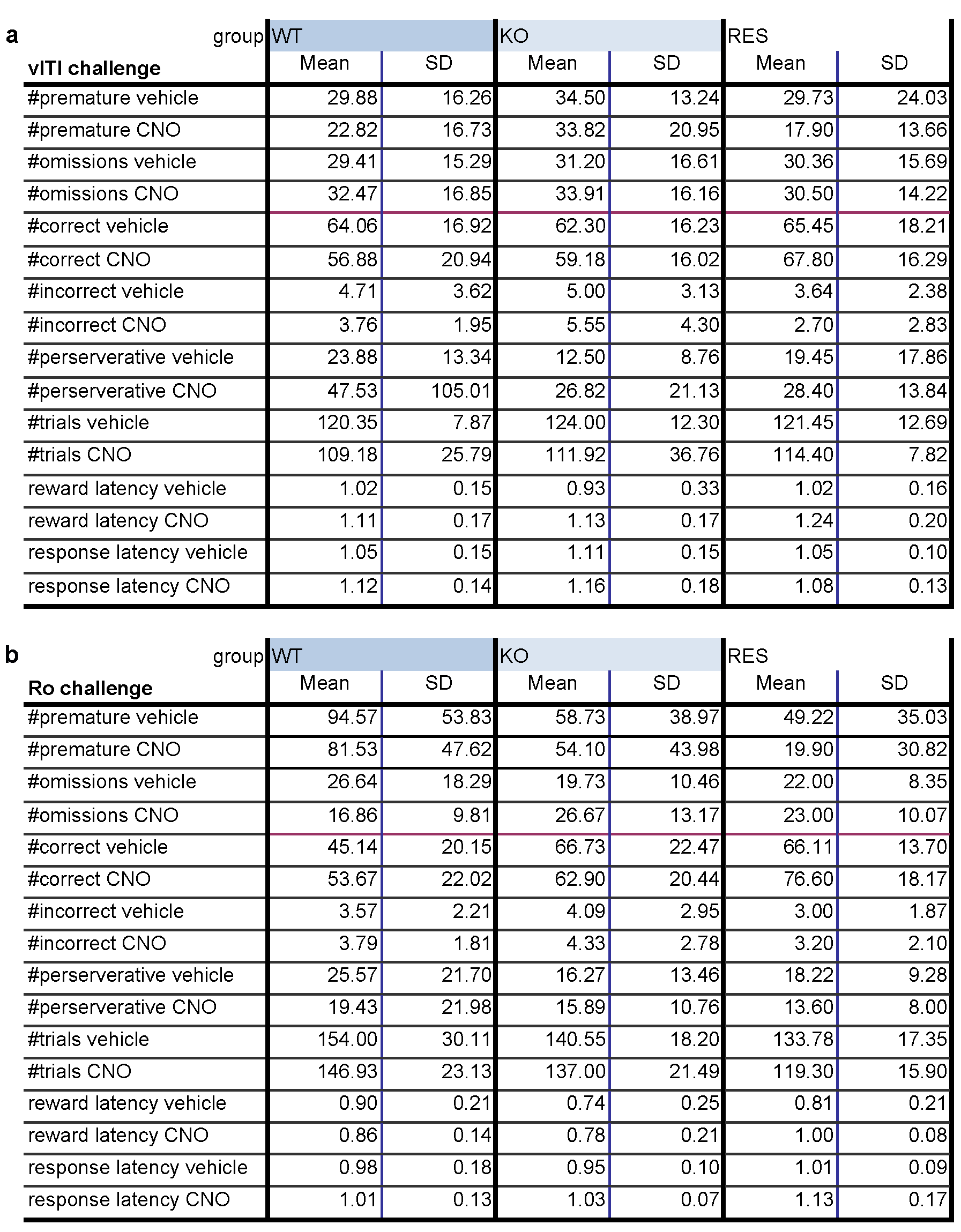


**Supplementary Table 16 | Absolute values in behavioural experiments in Tacr1-cohort.** (**a-e**) The absolute number of all types of behavioural responses and latencies are shown for the two testing conditions done in the Tacr1-KO cohort as displayed in Fig. 7 and identified in the top left corner of each panel. SD, standard deviation


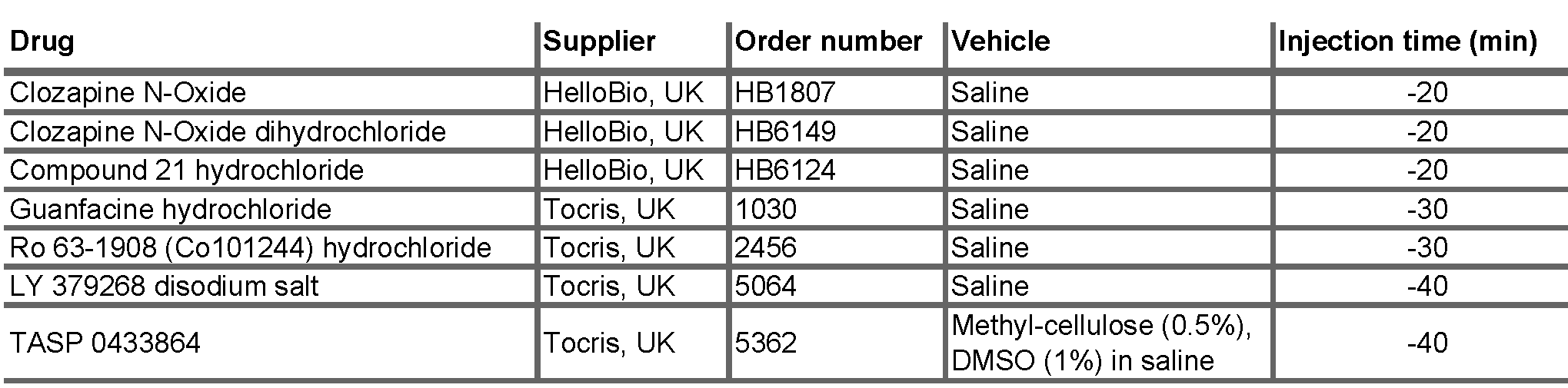


**Supplementary Table 17 | Overview over used compounds.** The injection time refers to the minutes relative to the start of the 30 min 5-CSRTT session (= time point 0 min). For the first two batches of CamKIIα-Cre the free base of CNO was used and dissolved at 1 mg/ml. As concerns were expressed by the supplier and other producers about the maintenance of CNO free base in aqueous solution (which we, however, could not confirm), for all remaining chemogenetic experiments the more water-soluble version CNO dihydrochloride was used and diluted at a concentration of 1 mg compound in 0.826 ml saline in order to maintain the concentration of free CNO at 1 mg/ml that was used with the free-base. All dose information stated for CNO in this manuscript therefore relate to the free base component, to ensure equal concentrations across experiments.

## Supplementary Code

**IgorPro code to extract differentially expressed GPCR genes from results files provided by CytosploreViewer.**

#pragma rtGlobals=3 // Use modern global access method and strict wave access.

// before running the code, ensure that the list with GPCR names was loaded into the Igor expt file and named "GPCR_list"

//the mouse GPCR-list used has 402 entries, the human GPCR list 399 - change the entry of the variable "length" depending on analysis done

function pullGPCRs() // copy-paste this function name into the Command line, press enter to start the function

wave/T GPCR_list, X_Gene

wave Diff_Mean, BetaW, Corrected_P_Value, Selection_1_Mean, Selection_2_Mean

variable length = 399 // mouse data set: 402, human data set: 399

make/o/n=(length) G_Position

make/o/n=(length) G_DiffMean

make/o/n=(length) G_BetaW

make/o/D/n=(length) G_CorrPVal

make/o/n=(length) G_Sel1Mean

make/o/n=(length) G_Sel2Mean

make/o/n=(length) order

make/o/n=(length) thresh

order = p

thresh = 3.32 // log2 of positive 10fold change

make/o/n=(100) S_DiffMean

make/o/n=(100) S_BetaW

make/o/D/n=(100) S_CorrPVal

make/o/n=(100) S_Sel2Mean

make/o/T/n=(100) S_GPCR

edit/k=1 order, GPCR_list, G_Position, G_DiffMean, G_BetaW, G_CorrPVal, G_Sel1Mean, G_Sel2Mean

variable n = 0

variable m = 0

string srchwrd

do

srchwrd = GPCR_list(n)

findvalue/TEXT=srchwrd/TXOP=2 X_Gene

G_Position[n] = V_value

G_DiffMean[n] = Diff_Mean[V_value]

G_BetaW[n] = BetaW[V_value]

G_CorrPVal[n] = Corrected_P_Value[V_value]

G_Sel1Mean[n] = Selection_1_Mean[V_value]

G_Sel2Mean[n] = Selection_2_Mean[V_value]

if((G_DiffMean[n]>thresh) && (G_CorrPVal[n]<0.05)) // 3.32 means that the expression (1+CPM) is 10x higher in Selection 1 compared to Selection 2

print srchwrd, " , logFC: ", G_DiffMean[n], " , CorrPVal: ", G_CorrPVal[n]

endif

if((G_DiffMean[n]>1.58) && (G_CorrPVal[n]<0.05)) // 1.58 means that the expression (1+CPM) is 3x higher in Selection 1 compared to Selection 2, for e-base use 1.09

S_GPCR[m] = srchwrd

S_DiffMean[m] = G_DiffMean[n]

S_BetaW[m] = G_BetaW[n]

S_CorrPVal[m] = G_CorrPVal[n]

S_Sel2Mean[m] = G_Sel2Mean[n]

m = m + 1

endif

n = n+1

while (n < length)

deletepoints m, 100-m, S_GPCR, S_DiffMean, S_BetaW, S_CorrPVal, S_Sel2Mean

duplicate/o S_DiffMean, S_thresh

S_thresh = 3.32 // for base e a 10x change corresponds to a log of 2.30

display/k=1 G_DiffMean

ModifyGraph mode=1,rgb=(0,0,0), zColor(G_DiffMean)={G_CorrPVal,0.0001,1,YellowHot,0}, logZColor(G_DiffMean)=1, nticks(bottom)=10 // zColor(G_logFC)={G_CorrPVal,*,*,Grays,0} //zColor(G_logFC)={G_CorrPVal,*,*,Rainbow,0}

Appendtograph thresh

ModifyGraph rgb(thresh)=(39168,39168,39168), margin(right)=100

SetAxis left -6,10; Label left "log2(fold-change)"

ColorScale/C/N=text0/F=0/A=RT/E=2 trace=G_DiffMean,axisRange={1,0.0001},log=1;DelayUpdate

ColorScale/C/N=text0 lblMargin=0;DelayUpdate

ColorScale/C/N=text0 "Corrected P-value"

ColorScale/C/N=text0/X=0.00/Y=5.00

Sort S_DiffMean S_DiffMean, S_GPCR, S_BetaW, S_CorrPVal, S_Sel2Mean

display/k=1 S_DiffMean vs S_GPCR

ModifyGraph mode=5, hbFill(S_DiffMean)=2, zColor(S_DiffMean)={S_Sel2Mean,0,8,Grays,0}, margin(right)=100, tkLblRot(bottom)=90

ModifyGraph hbFill(S_DiffMean)=2

SetAxis left 0,6; Label left "log2(fold-change)"

ColorScale/C/N=text0/A=RT/X=-1/Y=5/E=2 ctab={0,8,Grays,0}

ColorScale/C/N=text0 "log2(Set2 expression)"

appendtograph/L/NCAT S_thresh; DelayUpdate;

ModifyGraph mode(S_thresh)=0,lstyle(S_thresh)=3,rgb(S_thresh)=(43520,43520,43520)

AppendToGraph/R/B S_BetaW vs S_GPCR

ModifyGraph toMode=0,mode(S_BetaW)=2,lsize(S_BetaW)=5,rgb(S_BetaW)=(16384,28160,65280), lblMargin(right)=60

Label right "Beta"

SetAxis right 0,1

ModifyGraph axRGB(right)=(16384,28160,65280),tlblRGB(right)=(16384,28160,65280),alblRGB(right)=(16384,28160,65280)

edit/k=1 S_GPCR, S_DiffMean, S_BetaW, S_CorrPVal, S_Sel2Mean

END

## Supplementary References

1. Bathgate, R. A., Ivell, R., Sanborn, B. M., Sherwood, O. D. & Summers, R. J. International Union of Pharmacology LVII: recommendations for the nomenclature of receptors for relaxin family peptides. *Pharmacol. Rev.* **58**, 7–31 (2006).

2. Halls, M. L., Bathgate, R. A. D. & Summers, R. J. Comparison of signaling pathways activated by the relaxin family peptide receptors, RXFP1 and RXFP2, using reporter genes. *J. Pharmacol. Exp. Ther.* **320**, 281–290 (2007).

3. Adham, N. *et al.* Cloning of another human serotonin receptor (5-HT1F): a fifth 5-HT1 receptor subtype coupled to the inhibition of adenylate cyclase. *Proc. Natl. Acad. Sci.* **90**, 408–412 (1993).

4. Yudin, Y. & Rohacs, T. Inhibitory Gi/O-coupled receptors in somatosensory neurons: Potential therapeutic targets for novel analgesics. *Mol. Pain* **14**, 1744806918763646 (2018).

5. de Oliveira, P. G., Ramos, M. L. S., Amaro, A. J., Dias, R. A. & Vieira, S. I. Gi/o-Protein Coupled Receptors in the Aging Brain. *Front. Aging Neurosci.* **11**, (2019).

6. Janušonis, S. Functional associations among G protein-coupled neurotransmitter receptors in the human brain. *BMC Neurosci.* **15**, 1–19 (2014).

7. Benn, A. & Robinson, E. S. J. Investigating Glutamatergic Mechanism in Attention and Impulse Control Using Rats in a Modified 5-Choice Serial Reaction Time Task. *PLoS ONE* **9**, e115374 (2014).

8. Chudasama, Y. *et al.* Dissociable aspects of performance on the 5-choice serial reaction time task following lesions of the dorsal anterior cingulate, infralimbic and orbitofrontal cortex in the rat: differential effects on selectivity, impulsivity and compulsivity. *Behav. Brain Res.* **146**, 105–119 (2003).

9. Granon, S. *et al.* Enhanced and Impaired Attentional Performance After Infusion of D1 Dopaminergic Receptor Agents into Rat Prefrontal Cortex. *J. Neurosci.* **20**, 1208–1215 (2000).

10. Jupp, B. *et al.* Diminished Myoinositol in Ventromedial Prefrontal Cortex Modulates the Endophenotype of Impulsivity. *Cereb. Cortex* **bhz317**, (2020).

11. Kim, H., Ährlund-Richter, S., Wang, X., Deisseroth, K. & Carlén, M. Prefrontal Parvalbumin Neurons in Control of Attention. *Cell* **164**, 208–218 (2016).

12. Koike, H. *et al.* Chemogenetic Inactivation of Dorsal Anterior Cingulate Cortex Neurons Disrupts Attentional Behavior in Mouse. *Neuropsychopharmacology* **41**, 1014–1023 (2016).

13. Koskinen, T., Ruotsalainen, S. & Sirviö, J. The 5-HT2 Receptor Activation Enhances Impulsive Responding Without Increasing Motor Activity in Rats. *Pharmacol. Biochem. Behav.* **66**, 729–738 (2000).

14. Loos, M. *et al.* Neuregulin-3 in the Mouse Medial Prefrontal Cortex Regulates Impulsive Action. *Biol. Psychiatry* **76**, 648–655 (2014).

15. Loos, M. *et al.* Prefrontal cortical neuregulin-ErbB modulation of inhibitory control in rats. *Eur. J. Pharmacol.* **781**, 157–163 (2016).

16. Luchicchi, A. *et al.* Sustained Attentional States Require Distinct Temporal Involvement of the Dorsal and Ventral Medial Prefrontal Cortex. *Front. Neural Circuits* **10**, (2016).

17. Muir, J. L., Everitt, B. J. & Robbins, T. W. The Cerebral Cortex of the Rat and Visual Attentional Function: Dissociable Effects of Mediofrontal, Cingulate, Anterior Dorsolateral, and Parietal Cortex Lesions on a Five-Choice Serial Reaction Time Task. *Cereb. Cortex* **6**, 470–481 (1996).

18. Murphy, E. R., Dalley, J. W. & Robbins, T. W. Local glutamate receptor antagonism in the rat prefrontal cortex disrupts response inhibition in a visuospatial attentional task. *Psychopharmacology (Berl.)* **179**, 99–107 (2005).

19. Norman, K. J. *et al.* Post-error recruitment of frontal sensory cortical projections promotes attention in mice. *Neuron* (2021) doi:10.1016/j.neuron.2021.02.001.

20. Paine, T. A., Neve, R. L. & Carlezon, W. A. Attention Deficits and Hyperactivity Following Inhibition of cAMP-Dependent Protein Kinase Within the Medial Prefrontal Cortex of Rats. *Neuropsychopharmacology* **34**, 2143–2155 (2009).

21. Paine, T. A., Slipp, L. E. & Carlezon, W. A. Schizophrenia-Like Attentional Deficits Following Blockade of Prefrontal Cortex GABA A Receptors. *Neuropsychopharmacology* **36**, 1703–1713 (2011).

22. Paine, T. A., Cooke, E. K. & Lowes, D. C. Effects of chronic inhibition of GABA synthesis on attention and impulse control. *Pharmacol. Biochem. Behav.* **135**, 97–104 (2015).

23. Passetti, F., Chudasama, Y. & Robbins, T. W. The Frontal Cortex of the Rat and Visual Attentional Performance: Dissociable Functions of Distinct Medial Prefrontal Subregions. *Cereb. Cortex* **12**, 1254–1268 (2002).

24. Robinson, E. S. J. *et al.* Opposing Roles for 5-HT2A and 5-HT2C Receptors in the Nucleus Accumbens on Inhibitory Response Control in the 5-Choice Serial Reaction Time Task. *Neuropsychopharmacology* **33**, 2398–2406 (2007).

25. Warthen, D. M. *et al.* Activation of Pyramidal Neurons in Mouse Medial Prefrontal Cortex Enhances Food-Seeking Behavior While Reducing Impulsivity in the Absence of an Effect on Food Intake. *Front. Behav. Neurosci.* **10**, (2016).

26. Winstanley, C. A. *et al.* Intra-prefrontal 8-OH-DPAT and M100907 improve visuospatial attention and decrease impulsivity on the five-choice serial reaction time task in rats. *Psychopharmacology (Berl.)* **167**, 304–314 (2003).

27. Zhang, W. *et al.* Downregulation of 5-hydroxytryptamine7 receptor in the medial prefrontal cortex ameliorates impulsive actions in animal models of schizophrenia. *Behav. Brain Res.* **341**, 212–223 (2018).

28. Hodge, R. D. *et al.* Conserved cell types with divergent features in human versus mouse cortex. *Nature* **573**, 61–68 (2019).
