# Supplementary material for: Control of impulsivity by Gi-protein signalling in layer-5 pyramidal neurons of the anterior cingulate cortex
Source: Commun Biol. 2021 Jun 2;4:662. doi: 10.1038/s42003-021-02188-w (PMC8172539; doi:10.1038/s42003-021-02188-w)
Supplement: Supplementary file 4 — Description of Supplementary Files [file 42003_2021_2188_MOESM4_ESM.pdf]

Description of Additional Supplementary Files

File name: Supplementary Data 1

Description: *Source data for main figures.* Numeric source data for all main figures is provided as a separate Excel file. Each sheet is named by the respective panels to which the source data is relevant. Where data for other variables assessed by the 5-CSRTT is also stated (as analysed in Supplementary Tables 4, 6, 7, 9-15), the variables plotted in the actual figure are identified by green highlighting and the statement of the figure panel where the data appears at the top of the column. Abbreviated variable names are explained in the legend below the data in each sheet.

File name: Supplementary Data 2

Description: *Differential gene expression analysis.* Supplementary Data 2 is which contains the following information in the named sheets (in left-to-right order):

Sheet mACA contrasts
Names of all individual ACC cells (black) that are included in the original dataset from Hodge et al. [28] sorted according to the clusters that they were assigned to in the metadata (coloured column headings), with the total number of cells in the given cluster stated above the cluster name. Cell names highlighted orange were pulled down as Rbp4-Cre::tdTomato+ and were included in the equally named Set-T for the analysis presented in Fig. 5a-b and Supplementary Fig. 3a-d, given that they were also glutamatergic. All clusters in red font were part of the Set-T L4/5-IT in the same analyses, and clusters in blue font were part of some or all of the three versions of Set-C (see Methods). Clusters in green were not included in either set, unless they were also Rbp4-Cre::tdTomato+, in which case they were part of that Set-T (see above).
The lists of cell names for the target and non-target (contrast) sets of clusters were assembled based on the metadata of the mouse *anterior cingulate area* (ACA) gene expression dataset [28] (available at https://portal.brain-map.org/ , see Supplementary Table 10). Firstly, only cells from the ACA region (5190 cells with the *ACA* entry in the column region_label) were selected, and then further separated based on the columns class_label, subclass_label, and – for the Rbp-Cre::tdTomato-positive cells - full_genotype_label. Cells with the class_label entry *Exclude* or the cell_type_designation_label *Low Quality* were excluded from the further analysis because of suspected insufficient quality (*N* = 68). A total of two target sets (Set-T) and three non-target (or contrast) sets (Set-C) of cells were assembled. The first target set -

capturing 676 cells pulled down based on Cre-activity in the Rbp4-Cre driver line - included all cells that were assigned the class_label *Glutamatergic* and the full_genotype_label *(Rbp4-Cre_KL100/wt;Ai14(RCL-tdT)/wt)*, which were also all designated as *RFP-positive* (column: facs_population_plan_label). The second target set – capturing 1238 inter-telencephalic-projecting (IT) cells of layer 5 – were identified from the subclass labels *L4/5 IT* (*N* = 839), *L5 IT* (*N* = 168), and *RSP/ACA L4/5 IT* (*N* = 231). Note that this set is substantially larger than the first target set because not all layer 5 cells in the dataset were isolated in Rbp4-Cre::tdTomato mice; a considerable fraction was isolated using other markers, especially the pan-glutamatergic marker *Slc17a7* [28]. The three non-target sets all involved 691 GABAergic cells (class_label *GABAergic*), two of them additionally involved 2285 layer 6 cells (subclass_label *L6 CT*, *L6 IT*, *L6b*) and 14 non-neuronal cells (class_label *Non-neuronal*), and one of them additionally 524 excitatory layer 2/3 cells (subclass_label *L2/3 IT Cxcl14*, *L2/3 IT Otof*); the latter, most conservative, Set-C was used for the main analysis displayed in Fig. 5.

Sheet Cell_lists_Fig.5, Cell_lists_FigS4

The three lists of cells (Set-T: Rbp4-Cre::tdTomato+; Set-T: L4/5 IT; Set-C: Negative Contrast Set) that were included in the analysis for Fig. 5a and 5b (Cell_lists_Fig.5) and the three lists of cells (Set-T: L5 IT RORB; Set-T: all L5: Set-C: Negative Contrast Set) that are included in the analysis shown in Supplementary Fig. S4 (Cell_lists_FigS4). The names of individual clusters that contributed to each set (identical to the names in Sheet *mACA contrasts* or *hCgC contrasts,* respectively) are listed in the corresponding cell above, in row 3.

Sheet GPCRs

List of all 402 mouse and 399 human non-sensory GPCR-encoding genes that were included in the analysis.

The list of non-sensory GPCR genes was assembled by comparing various sources, including primarily the 380 GPCRs analysed in the TaqMan Mouse GPCR Array (part# 4378718, Applied Biosystems / ThermoFisher Scientific, US) and the GPCRs listed in the HGNC human genome database (https://www.genenames.org/data/genegroup/#!/group/139). The lists of mouse and human GPCRs from these two sources were cross-checked manually against each other and missing GPCR genes were filled in using the NCBI (https://www.ncbi.nlm.nih.gov/) *Gene* database, a dedicated curated GPCR database (https://gpcrdb.org), and Uniprot (https://www.uniprot.org/). In the case of multiple common names for the same gene, synonyms were cross-checked against the names of GPCR genes in the list of analysed transcripts from the Allen Institute for Brain Science mouse gene expression database (https://portal.brain-map.org/).

Sheets Rbp4vGABA_L23_L6, L45ITvGABA_L23_L6, Rbp4vGABA_L6, L45ITvGABA_L6 Rbp4vGABA, L45ITvGABA, hL45ITvSetC, hL5vSetC

Results of the differential gene expression analyses across 402 GPCR-encoding genes (in rows, named in column B in alphabetical order), for which the significantly differentially expressed genes with a fold-change (FC) > 3 (i.e. $\log_2$(FC) > 1.58) are displayed in Fig.s 5a (Rbp4vGABA_L23_L6), 5b (L45ITvGABA_L23_L6), or Supplementary Fig.s 3a (Rbp4vGABA_L6), 3b (L45ITvGABA_L6), 3c (Rbp4vGABA), 3d (L45ITvGABA), and 4 (hL45ITvSetC, hL5vSetC) respectively. Column A contains the order number for reference to Supplementary Fig. 3e. Column C (DiffMean) contains the $\log_2$(FC) (which is equal to the difference of the $\log_2$-transformed mean expression values within each set (measured as CPM+1)). Column D contains the Beta values to measure the separation of both sets according to the expression of the given gene in each set (see Methods). Column E (CorrPVal) is the Bonferroni-corrected *P*-value. For the confirmatory analysis of the human dataset (hL45ITvSetC, hL5vSetC) the P-value is uncorrected. Columns F (Set-T) and G (Set-C) contain the mean expression value (as $\log_2$(CPM+1) in the given set of cells. Additional columns provide (somewhat redundant) information on the cluster names that are part of each set (Set-T or Set-C, as indicated) and the *N* for each of them, as well as short-lists for significantly differentially expressed GPCRs with a FC > 3 and FC > 10, as indicated, and a replication of the corresponding graphs from Fig. 5a-b and Supplementary Fig. 3a-d.
