# Supplementary material for: Control of impulsivity by Gi-protein signalling in layer-5 pyramidal neurons of the anterior cingulate cortex
Source: Commun Biol. 2021 Jun 2;4:662. doi: 10.1038/s42003-021-02188-w (PMC8172539; doi:10.1038/s42003-021-02188-w)
Supplement: Supplementary file 5 — Reporting Summary [file 42003_2021_2188_MOESM5_ESM.pdf]

nature research

Corresponding author(s): Dennis Kätzel

Last updated by author(s): 23/4/2021

Reporting Summary

Nature Research wishes to improve the reproducibility of the work that we publish. This form provides structure for consistency and transparency in reporting. For further information on Nature Research policies, see our Editorial Policies and the Editorial Policy Checklist.

Statistics

For all statistical analyses, confirm that the following items are present in the figure legend, table legend, main text, or Methods section.

n/a	Confirmed	
☐	☒	The exact sample size (n) for each experimental group/condition, given as a discrete number and unit of measurement
☐	☒	A statement on whether measurements were taken from distinct samples or whether the same sample was measured repeatedly
☐	☒	The statistical test(s) used AND whether they are one- or two-sided<br>*Only common tests should be described solely by name; describe more complex techniques in the Methods section.*
☐	☒	A description of all covariates tested
☐	☒	A description of any assumptions or corrections, such as tests of normality and adjustment for multiple comparisons
☐	☒	A full description of the statistical parameters including central tendency (e.g. means) or other basic estimates (e.g. regression coefficient) AND variation (e.g. standard deviation) or associated estimates of uncertainty (e.g. confidence intervals)
☐	☒	For null hypothesis testing, the test statistic (e.g. F, t, r) with confidence intervals, effect sizes, degrees of freedom and P value noted<br>*Give P values as exact values whenever suitable.*
☒	☐	For Bayesian analysis, information on the choice of priors and Markov chain Monte Carlo settings
☒	☐	For hierarchical and complex designs, identification of the appropriate level for tests and full reporting of outcomes
☒	☐	Estimates of effect sizes (e.g. Cohen's d, Pearson's r), indicating how they were calculated

Our web collection on statistics for biologists contains articles on many of the points above.

Software and code

Policy information about availability of computer code

Data collection	Scripts of behavioural tasks done in custom-designed operant boxes are available from https://github.com/KaetzelLab/Operant-Box-Code
Data analysis	Behavioural data was analysed using SPSS 26.0 (IBM, NY, US). Custom-Code used in IgorPro 6 (Wavemetrics, Inc) are included in Supplementary Information as Supplementary Code

For manuscripts utilizing custom algorithms or software that are central to the research but not yet described in published literature, software must be made available to editors and reviewers. We strongly encourage code deposition in a community repository (e.g. GitHub). See the Nature Research guidelines for submitting code & software for further information.

Data

Policy information about availability of data

All manuscripts must include a data availability statement. This statement should provide the following information, where applicable:

- Accession codes, unique identifiers, or web links for publicly available datasets
- A list of figures that have associated raw data
- A description of any restrictions on data availability

All raw data for behavioural experiments can be obtained from the corresponding author upon reasonable request and all source data for main figures is supplied as Supplementary Source Data 2. Gene-expression data is available from the Allen Institute for Brain Sciences (https://portal.brain-map.org/) and through (CytosploreViewer https://viewer.cytosplore.org); source data for the differential gene expression analysis of GPCRs is available as Supplementary Source Data 1.

Field-specific reporting

Please select the one below that is the best fit for your research. If you are not sure, read the appropriate sections before making your selection.

- [x] Life sciences
- [ ] Behavioural & social sciences
- [ ] Ecological, evolutionary & environmental sciences

For a reference copy of the document with all sections, see nature.com/documents/nr-reporting-summary-flat.pdf

Life sciences study design

All studies must disclose on these points even when the disclosure is negative.

Sample size	Sample-size calculation was conducted as power-calculation prior to the experiment based on prior 5-choice-serial-reaction-time task data from wildtype animals, resulting in a recommended sample size of n = 16 per group (including 25% reserve animals for potential exclusions). These calculations do not capture or represent chemogenetic effects, however, and therefore only served as approximate guidance.
Data exclusions	Some mice were excluded from all datasets based on the specificity of viral transfection: Animals were only included in the datasets if they showed bilateral expression in the majority of the volume of the target structure but not in any other brain region. While minor and unilateral off-site expression in a neighbouring PFC subregion was tolerated, unilateral expression in M2 or M1 also led to exclusion of the animal given that this could potentially affect motor activity rather directly. mCherry-transduced control animals were not excluded based on expression patterns.<br>In addition, some mice were excluded from specific individual experiments due to one of the following reasons: Not meeting criteria before the experiment, hardware/software failure of the operant task, ill health during the experiment, the mouse being overweight, post-ip injection behaviour or the application of the wrong drug-dose.<br>Such reasons for data exclusion are stated in Supplementary Table 3 for each experiment, listing the number of excluded mice for each group.
Replication	Key findings of chemogenetic and pharmacological manipulations were replicated within-subject (Suppl. Fig. 1 for Fig. 1; Fig. 2i for 2f-g, Suppl. Fig. 5 for Fig. 6a-b); effects in independent ACC-hM4Di cohorts confirm each other (Fig. 1/3/7).
Randomization	Group-identity (transfection) was determined by counter-balancing according to 5-CSRTT performance before surgery (Fig. 1-2, 7); all drug-applications for within-subject studies were determined by a cross-over (latin-square) design counter-balanced within each group
Blinding	The experimenter was blind to group-identity during the experiments and data processing.

Reporting for specific materials, systems and methods

We require information from authors about some types of materials, experimental systems and methods used in many studies. Here, indicate whether each material, system or method listed is relevant to your study. If you are not sure if a list item applies to your research, read the appropriate section before selecting a response.

Materials & experimental systems

n/a	Involved in the study	
x		Antibodies
x		Eukaryotic cell lines
x		Palaeontology and archaeology
	x	Animals and other organisms
x		Human research participants
x		Clinical data
x		Dual use research of concern

Methods

n/a	Involved in the study	
x		ChIP-seq
x		Flow cytometry
x		MRI-based neuroimaging

Animals and other organisms

Policy information about studies involving animals; ARRIVE guidelines recommended for reporting animal research

Laboratory animals	In total, 67 male B6.Cg-Tg(Camk2a-cre)T29-1Stl/J (CamKIIa-Cre, obtained from The Jackson Laboratory, Maine, US), 79 male B6.FVB (Cg)-Tg(Rbp4-cre)KL100Gsat/Mmucd (Rbp4-Cre, obtained originally from GENSAT, US), 18 male C57BL/6J wildtype mice, as well as 27 Tacr1tm1Sph mice (Tacr1-/-) and 17 wildtype littermates of both sexes - all obtained from breeding of heterozygous Tacr1+/- parents 62 - were used for this study. The mice were of 2-3 months of age at the start of the experiments, which took up to 11 months to complete.
Wild animals	none
Field-collected samples	none

Ethics oversight	All experiments were performed in accordance to the German Animal Rights Law (Tierschutzgesetz) 2013 and were approved by the Federal Ethical Review Committee (Regierungsprädsidium Tübingen) of Baden-Württemberg, Germany (licence number TV1344).

Note that full information on the approval of the study protocol must also be provided in the manuscript.
